# Synthesis, spectroscopic characterization, and DFT-assisted molecular docking analysis of novel 1,3,4-oxadiazole–1,2,3-triazole hybrids with antimicrobial and cytotoxicity potential

**DOI:** 10.1039/d5ra09082b

**Published:** 2026-01-30

**Authors:** Darshna K. Lakhnotra, Jay B. Maheta, Yogesh O. Bhola, Bhavesh N. Socha, Nargis H. Shaikh, Prince A. Dave, Sureshkumar B. Koradiya

**Affiliations:** a Department of Chemistry, ShriM. P. Pandya Science College, Lunawada, Mahisagar, Shri Govind Guru University Godhra Gujarat India yogeshbhola90@gmail.com dryobhola@gmail.com; b Department of Chemistry, Mahisagar Science College-Lunawada, Mahisagar, Shri Govind Guru University-Godhra Gujarat India; c Department of Materials Science, Sardar Patel University Vallabh Vidyanagar-388120 Gujarat India

## Abstract

A novel series of heterocyclic derivatives (10a–i, 11a–d) was successfully synthesized and evaluated through a synergistic combination of density functional theory (DFT), molecular docking, and *in vitro* biological assays to explore their potential as multifunctional therapeutic agents. Theoretical investigations revealed that compounds 11b, 10f, 10i, 11c, and 10d exhibited favourable electronic properties, including optimal HOMO–LUMO energy gaps and high electrophilicity indices, which correlate with enhanced chemical stability and reactivity. Molecular docking analysis demonstrated strong binding affinities toward Thymidylate Kinase (4QGG) and Epidermal Growth Factor Receptor (EGFR, 3W2Q), with compound 11b showing the best antimicrobial interaction energy (−5.89 kcal mol^−1^), while 10i and 11c showed strong binding complementarity with the EGFR active sites, suggesting potent cytotoxicity potential. *In vitro* results further validated the computational predictions. Compound 10i exhibited exceptional cytotoxicity against both MCF-7 (IC_50_ = 1.2 ± 0.5 µM) and HepG2 (IC_50_ = 0.8 ± 0.2 µM) cell lines, demonstrating submicromolar potency against liver cancer cells and representing the most active compound in the entire series. Additionally, compound 10i showed significant EGFR inhibition (IC_50_ = 0.93 ± 0.25 µM), comparable to doxorubicin, whereas 11c displayed excellent EGFR inhibition (IC_50_ = 0.33 ± 0.06 µM), approaching erlotinib's potency. Furthermore, 11b exhibited potent and broad-spectrum antimicrobial activity (MICs of 1.89–4.61 µg mL^−1^), surpassing those of ciprofloxacin and griseofulvin. The combined computational and experimental findings highlight the significance of nitrogen- and oxygen-rich heteroaromatic functionalities, which enhance electronic distribution, molecular stability, and target recognition. Overall, compounds 10i, 11b, and 11c emerged as promising lead candidates with cytotoxicity and antimicrobial activities, with 10i demonstrating particularly remarkable broad-spectrum cytotoxicity efficacy, providing a rational framework for the design and development of next-generation multifunctional therapeutic agents.

## Abstract 2

Novel heterocyclic derivatives were synthesized and evaluated *via* DFT, molecular docking and bioassays. Compounds 10i, 11b and 11c showed strong EGFR inhibition, cytotoxicity and antimicrobial activity, highlighting their potential as multifunctional therapeutic leads.

## Graphical abstract

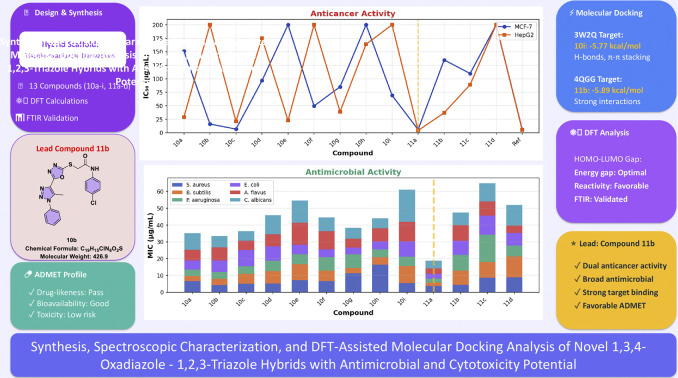

## 1. Introduction

Cancer, the second leading cause of death worldwide, remains one of the most challenging and multifactorial diseases in modern medicine.^1^ According to the Global Cancer Observatory (GLOBOCAN) 2020 report, there were approximately 19.3 million new cancer cases and more than 10 million cancer-related deaths worldwide.^2^ With the increasing global population and aging demographics, the number of new cases is projected to rise dramatically, reaching nearly 420 million by 2025.^3^ Despite significant progress in oncology, conventional cancer therapies, including chemotherapy, radiotherapy, and targeted therapy, are often limited by poor selectivity, reduced efficacy, and severe side effects. Moreover, the frequent emergence of multidrug resistance (MDR) further compromises treatment outcomes and highlights the need for more selective and potent anticancer agents. To overcome these challenges, the rational design of hybrid pharmacophores has emerged as a powerful strategy in modern drug discovery. Hybrid molecules, which integrate two or more bioactive scaffolds into a single framework, can synergistically enhance pharmacokinetic and pharmacodynamic profiles, increase selectivity, reduce toxicity, and counteract drug resistance. Recent advances in medicinal chemistry have demonstrated that such hybrid structures exhibit improved biological activity and therapeutic potential, with several hybrid-based compounds currently undergoing preclinical and clinical evaluation for cancer treatment.^4^

**Fig. 1:**
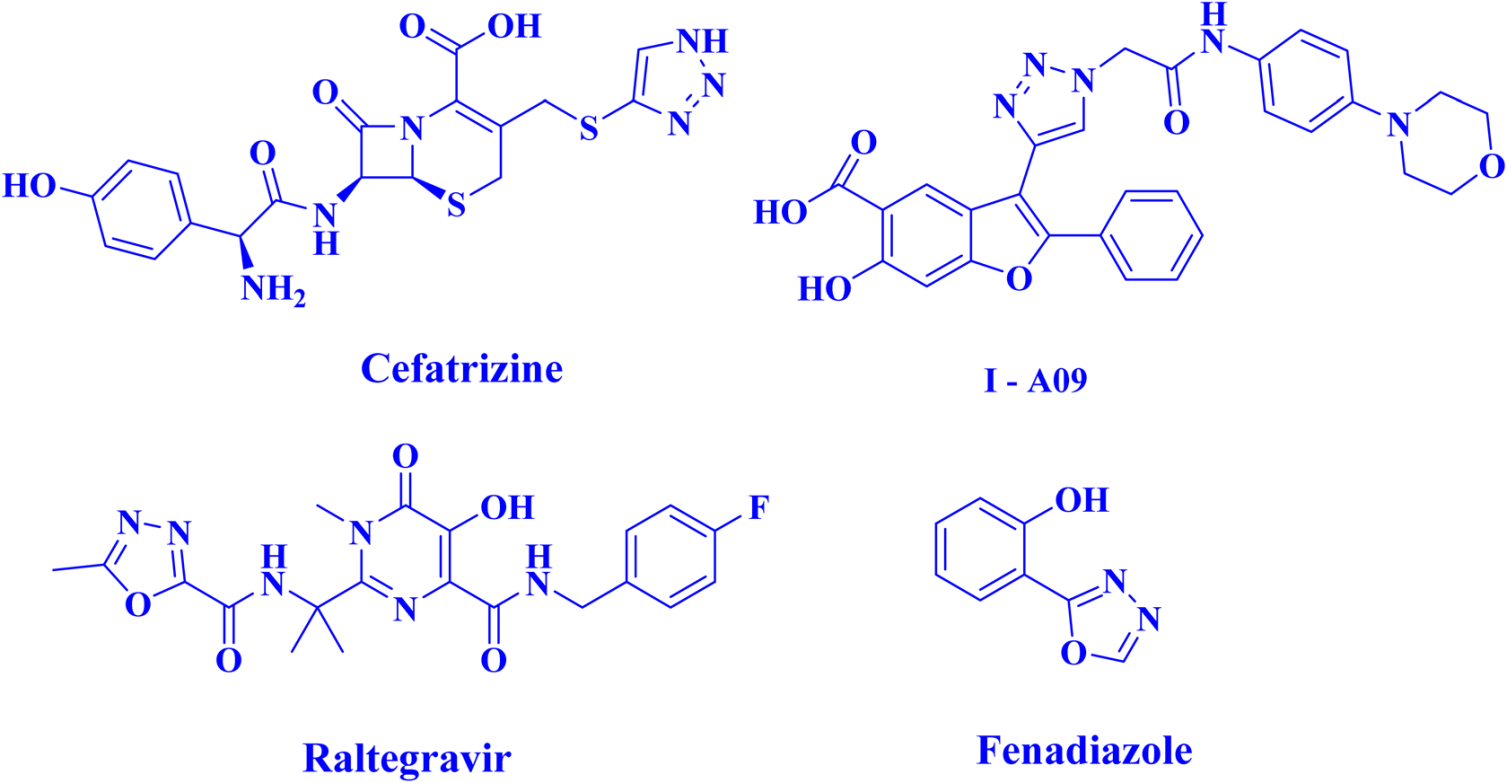
Structures of clinically used drugs incorporating oxadiazole and triazole moieties, demonstrating the pharmaceutical importance of these heterocyclic frameworks.

Among the diverse heterocyclic scaffolds explored in drug design, 1,3,4-oxadiazole and 1,2,3-triazole motifs have gained particular attention for their wide range of pharmacological properties and as bioisosteric replacements for carbonyl-containing functionalities such as carboxylic acids, esters, and amides.^5^ Several FDA-approved drugs contain these heterocyclic units, including the antiviral agent Raltegravir,^6^ the hypnotic Fenadiazole,^7^ the anticancer agent Cefatrizine,^8^ and the antitubercular drug I-A09.^9^ The literature further reports that compounds bearing 1,3,4-oxadiazole and 1,2,3-triazole rings exhibit diverse biological activity, including antibacterial,^10,11^ antidiabetic,^12^ antimicrobial,^13,14^ anti-inflammatory,^15^ anticancer,^16–18^ antioxidant,^19,20^ and antiparasitic^21,22^ properties. Significantly, even subtle structural modifications within these heterocycles can influence their biological behavior and target selectivity^23^ (Fig. 1).

In this context, the present study focuses on the design and synthesis of novel 1,3,4-oxadiazole ether derivatives incorporating an *N*-phenylacetamide moiety, aiming to enhance their antioxidant and anticancer potential while minimizing toxicity. To achieve this goal efficiently and rationally, an integrated computational–experimental approach has been adopted. Initially, quantum chemical calculations (DFT) and molecular docking studies are employed to predict the most stable molecular conformations, assess reactivity descriptors, and elucidate the binding interactions of the designed molecules with relevant cancer-associated targets. Furthermore, ADMET (Absorption, Distribution, Metabolism, Excretion, and Toxicity) profiling was performed to evaluate their pharmacokinetic and safety properties, thereby identifying the most promising candidates among the synthesized derivatives. The use of computational chemistry in early-stage drug discovery significantly reduces experimental cost and time while providing deep insights into molecular reactivity, stability, and binding behavior. Following these computational evaluations, the most active and stable compounds was synthesized and subjected to *in vitro* biological assays to validate their antioxidant and anticancer activity. This hybrid computational–experimental strategy not only enhances the efficiency of lead identification but also strengthens the scientific foundation for the rational development of potent, selective, and less toxic anticancer agents.

## 2. Result & discussion

### 2.1 Chemistry

**Scheme 1:**
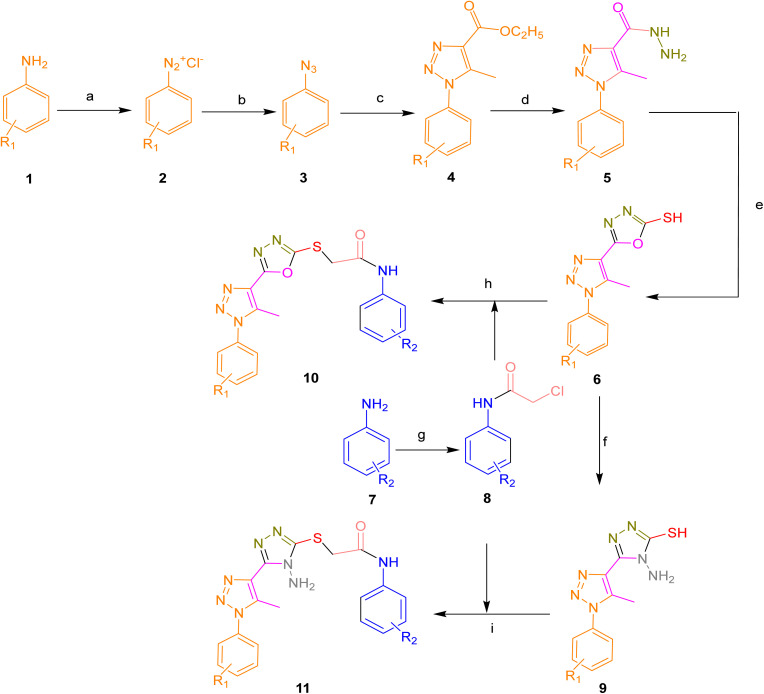
Reagents and conditions: (a) NaNO_2_, HCl, 0–5 °C (b) NaN_3_, 0–5 °C (c) EAA, NaOC_2_H_5_, C_2_H_5_OH, RT (d) EtOH, NH_2_NH_2_ H_2_O, 6 h reflux (e) CS_2_, KOH, C_2_H_5_OH, 4 h reflux (f) N_2_H_4_, C_2_H_5_OH, 4 h reflux (g) DMF, K_2_CO_3_, chloroacetyl chloride, 2 h reflux, (h) DMF, K_2_CO_3_, RT, (i) DMF, K_2_CO_3_, RT.

The target compounds 10a–i and 11a–d was efficiently synthesized through a convergent synthetic route, as illustrated in Scheme 1. The synthesis began with commercially available substituted aniline derivatives (1), which were treated with concentrated hydrochloric acid and sodium nitrite to generate the corresponding diazonium salts (2). Subsequent nucleophilic substitution with sodium azide afforded azido benzene derivatives (3), which then underwent 1,3-dipolar cycloaddition with ethyl acetoacetate in ethanol in the presence of sodium ethoxide to yield 1,2,3-triazole-based esters (4) in yields of 77–79%. These esters were further reacted with hydrazine hydrate in ethanol to give hydrazones (5), which served as key intermediates. The structure of compound 5 was confirmed by spectroscopic techniques, with characteristic signals in the ^1^H NMR spectrum including a singlet for the NH proton at *δ* 9.45, a triazole proton at *δ* 8.67, aromatic protons in the *δ* 6.8–8.5 range, and a methyl group at *δ* 2.35. The ^13^C NMR spectrum showed signals at *δ* 157 for the carbonyl carbon, *δ* 147 for the triazole C-4, and *δ* 125–139 for aromatic carbons. IR spectral data further supported the structure, displaying absorptions at 3325 cm^−1^ (NH stretch), 3089 cm^−1^ (triazole C–H), and 1660 cm^−1^ (C=O stretch) (Table 1).

**Table 1:** Experimental details for the synthesized target compounds 10a–i and 11a–d showing the influence of substituted aromatic groups (R and R_1_) on reaction time, percentage yield, and melting point profiles

Compounds	R_2_	R_1_	Reaction time^a^ (h)	Yield^b^ (%)	m.p^c^ (°C)
10a	H	H	3.30	81	202–204
10b	4 Cl	H	3.45	83	209–211
10c	H	4-OCH_3_	3.25	87	215–217
10d	4-OCH_3_	4-OCH_3_	3.34	82	200–202
10e	4-OCH_3_	H	3.16	90	210–212
10f	4-OCH_3_	3,4 di Cl	3.19	79	198–200
10g	H	3,4 di Cl	3.56	91	190–192
10h	4 Cl	3,4 di Cl	3.22	84	194–196
10i	3 Cl, 4 F	4-OCH_3_	3.25	82	214–216
11a	3 Cl, 4 F	3 Cl	4.36	86	211–213
11b	H	3 Cl	4.15	87	197–199
11c	4 Cl	3 Cl	4.31	83	218–220
11d	4-OCH_3_	3 Cl	4.26	81	189–191

a Reaction completion as visualised by TLC.

b Isolated yield.

c Melting points.

The hydrazones (5) were then cyclized with carbon disulfide in ethanol under basic conditions, KOH to afford 5-(5-methyl-1-phenyl-1*H*-1,2,3-triazol-4-yl)-1,3,4-oxadiazole-2-thiol derivatives (6). Spectral analysis confirmed the structures, with ^1^H NMR signals at *δ* 13.8 for the thiol proton, *δ* 2.65 for the methyl group, and aromatic protons appearing between *δ* 6.5–8.5. The ^13^C NMR spectrum showed a signal for the thiocarbonyl group (C=S) at *δ* 178, triazole C-4 at *δ* 147, and aromatic carbons in the *δ* 130–145 range. IR spectra displayed bands at 2560 cm^−1^ (S–H stretch), 1450–1650 cm^−1^ (aromatic C–H), and 1080–1120 cm^−1^ (C–O stretch).

In a parallel sequence, aniline derivatives (7) were acylated with chloroacetyl chloride in DMF using potassium carbonate as a base, yielding 2-chloro-*N*-phenylacetamide (8) in 82–86% yields. The thiol derivatives (6) were then reacted with compound 8 in the presence of potassium carbonate and DMF to produce the final 2-((5-(5-methyl-1-phenyl-1*H*-1,2,3-triazol-4-yl)-1,3,4-oxadiazol-2-yl)thio)-*N*-phenylacetamides (10a–i). The ^1^H NMR spectrum of these compounds showed a downfield singlet at *δ* 10.20 corresponding to the amide NH, a singlet at *δ* 4.25 for the S–CH_2_ group, and multiplets in the *δ* 6.5–8.3 region for aromatic protons. The ^13^C NMR spectra exhibited signals at *δ* 165 (C=O), *δ* 158 (oxadiazole C-5), *δ* 147 (triazole C-5), and *δ* 40 for the methylene carbon adjacent to sulfur. IR spectra supported these observations, displaying characteristic absorptions at 3300–3220 cm^−1^ (amide NH), 1660–1680 cm^−1^ (carbonyl), and 1450–1650 cm^−1^ (aromatic C=C).

Additionally, the thiol intermediates (6) were treated with hydrazine hydrate in ethanol to furnish 4-amino-5-(5-methyl-1-phenyl-1*H*-1,2,3-triazol-4-yl)-4*H*-1,2,4-triazole-3-thiol (9) in 79–81% yield. Spectral data confirmed the structure of compound 9 and was further used in nucleophilic substitution with 2-chloro-*N*-phenylacetamide (8) in the presence of DMF and potassium carbonate to obtain final derivatives 11a–d, structurally assigned as 2-((4-amino-5-(5-methyl-1-phenyl-1*H*-1,2,3-triazol-4-yl)-4*H*-1,2,4-triazol-3-yl)thio)-*N*-phenylacetamides. The ^1^H NMR spectra displayed singlets at *δ* 9.80 for the amide NH, *δ* 2.25 for the triazole methyl group, and aromatic protons appearing in the *δ* 6.50–8.50 range. The ^13^C NMR spectra showed diagnostic signals for triazole C=N and C–N bonds in the *δ* 165–170 region, aromatic carbons between *δ* 130–150, and the triazole methyl carbon at *δ* 20. IR spectra confirmed the presence of NH_2_ (3300 cm^−1^), amide C=O (1650–1700 cm^−1^), and aromatic C=C (1450–1590 cm^−1^) groups.

### 2.2 Molecular docking results

#### 2.2.1 Molecular docking studies against EGFR (PDB ID: 3W2Q)

To evaluate the cytotoxicity potential of the synthesized heterocyclic derivatives, molecular docking studies were performed against the epidermal growth factor receptor (EGFR) kinase domain, PDB ID: 3W2Q. The docking scores of the ligands ranged between −5.099 and −5.779 kcal mol^−1^, while the reference inhibitors Erlotinib and Doxorubicin exhibited docking scores of −5.292 and −6.588 kcal mol^−1^, respectively.

Among the tested ligands, compound 10i (−5.779 kcal mol^−1^) displayed the most favorable binding energy, surpassing Erlotinib and approaching Doxorubicin. The superior binding of 10i was attributed to multiple hydrogen bonds with LYS745, ASP855, and MET793, as well as halogen bonding interactions involving the F and Cl substituents. Strong electrostatic interactions with LYS875, ARG858, GLU762, and ASP837 further stabilised it within the ATP-binding cleft. Similarly, compound 11c (−5.762 kcal mol^−1^) exhibited robust interactions, particularly hydrogen bonding with GLU762 and hydrophobic contacts with LEU792, MET793, and PHE723, which are critical residues for kinase inhibition. Compounds 10d (−5.713 kcal mol^−1^) and 10c (−5.570 kcal mol^−1^) also showed favorable binding through hydrogen bonding with ASP855 and GLN791, supported by complementary polar and electrostatic contacts.

**Fig. 2:**
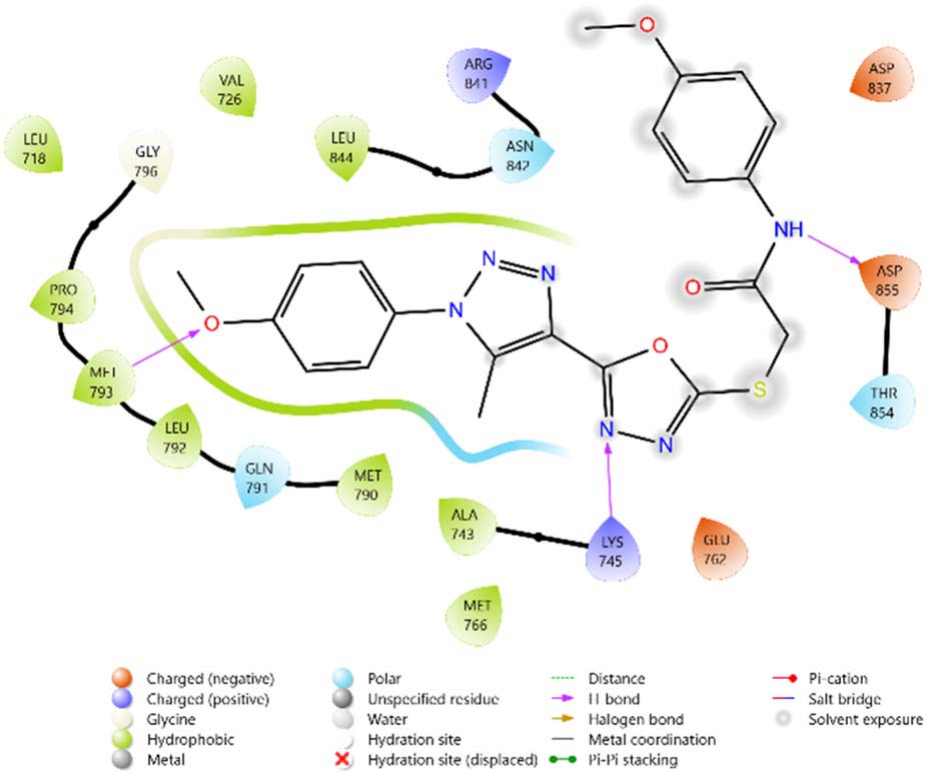
2D diagram of compound 10d docked with the 3W2Q protein target.

**Fig. 3:**
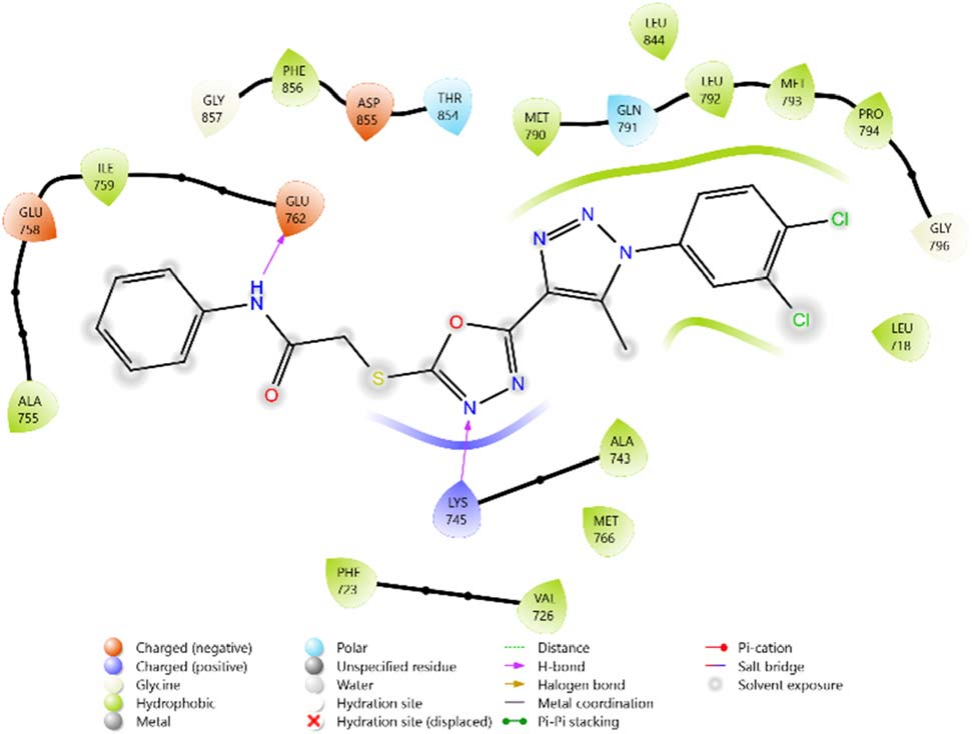
2D diagram of compound 10g docked with the 3W2Q protein target.

**Fig. 4:**
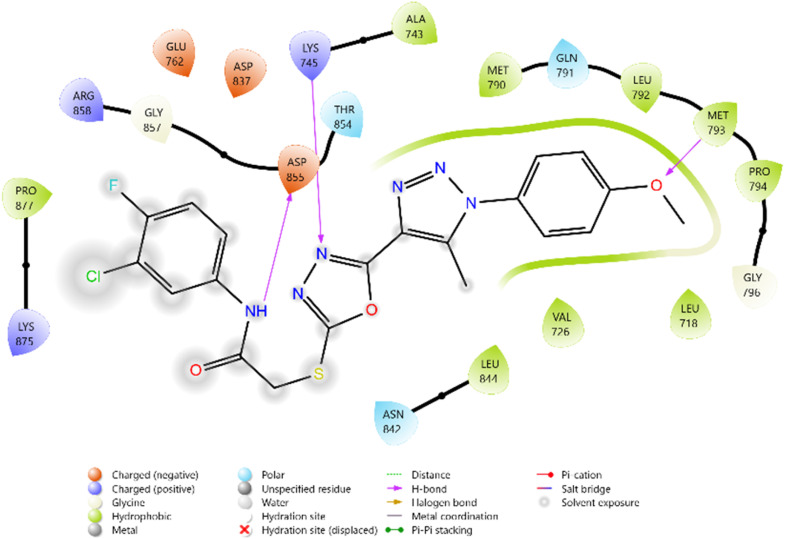
2D diagram of compound 10i docked with the 3W2Q protein target.

**Fig. 5:**
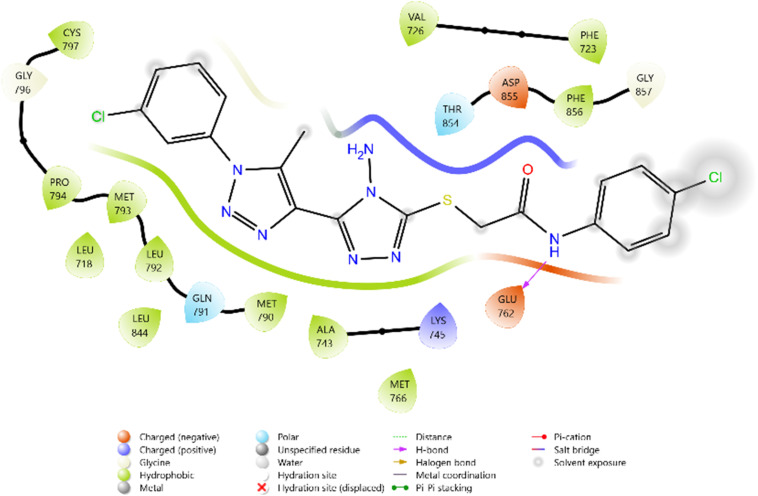
2D diagram of compound 11c docked with the 3W2Q protein target.

**Fig. 6:**
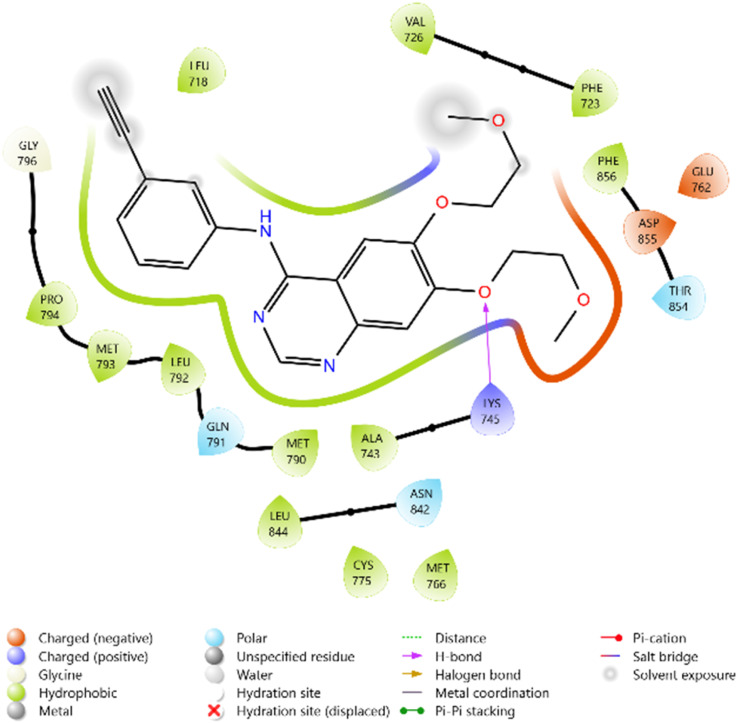
2D diagram of the standard drug Doxorubicin docked with the 3W2Q protein target.

**Fig. 7:**
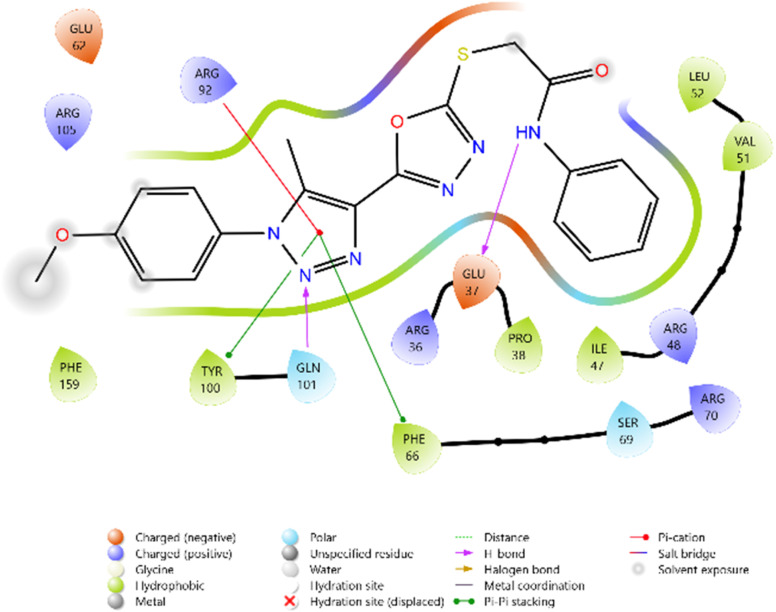
2D diagram of the standard drug Erlotinib docked with the 3W2Q protein target.

In comparison, Erlotinib interacted mainly with LYS745, GLN791, and ASN842, with a moderate docking score, whereas Doxorubicin achieved the highest binding affinity through extensive hydrogen bonding with LYS745, ASN842, and GLU762. Collectively, these results suggest the binding order 10i > 11c > 10d > 10c, highlighting these derivatives as promising anticancer candidates with EGFR inhibitory potential (Table 2 and Fig. 2–7).

**Table 2:** Molecular docking analysis of synthesized compounds with epidermal growth factor inhibitor (3W2Q)

Compound	Docking score (kcal mol^−1^)	Hydrophobic interactions	Polar interactions	Hydrogen bonds	Other interactions
10d	−5.713	LEU718, VAL726, PRO794, MET793, LEU792, MET790, ALA743, MET766, LEU844, GLY796	GLN791, ASN842, THR854	LYS745 (H-bond with nitrogen), ASP855 (H-bond with NH group)	ARG841 (charged positive), ASP837 (charged negative), ASP855 (charged negative), GLU762 (charged negative), LYS745 (charged positive)
10g	−5.707	ILE759, ALA755, GLY857, PHE856, LEU844, MET790, LEU792, MET793, PRO794, GLY796, LEU718, ALA743, MET766, PHE723, VAL726	GLN791, THR854	LYS745 (H-bond with nitrogen), GLU762 (H-bond with NH group)	GLU758 (charged negative), GLU762 (charged negative), ASP855 (charged negative), LYS745 (charged positive), halogen bonds with Cl substituents
10i	−5.779	PRO877, GLY857, ALA743, VAL726, LEU844, LEU718, GLY796, PRO794, MET793, LEU792, MET790	GLN791, THR854, ASN842	LYS745 (H-bond with nitrogen), ASP855 (multiple H-bonds), MET793 (H-bond interaction)	LYS875 (charged positive), ARG858 (charged positive), GLU762 (charged negative), ASP837 (charged negative), ASP855 (charged negative), LYS745 (charged positive), halogen bonds with F and Cl substituents
11c	−5.762	LEU 792, LEU 718, LEU 844, VAL 726, PHE 723, PHE 856, MET 790, MET 793, MET 766, PRO 794, ALA 743, CYS 797, GLY 796, GLY 857	THR 854, GLN 791	GLU762 (H-bond with NH group)	GLU762 (charged negative), ASP855 (charged negative), LYS745 (charged positive)
Erlotinib	−5.292	LEU 792, LEU 718, LEU 844, VAL 726, PHE 723, PHE 856, MET 790, MET 793, MET 766, PRO 794, ALA 743, CYS 775	THR 854, GLN 791, ASN 842	LYS745 (multiple H-bonds with oxygen)	GLU762 (charged negative), ASP855 (charged negative), LYS745 (charged positive)
Doxorubicin	−6.588	LEU 792, LEU 718, LEU 844, VAL 726, PHE 723, PHE 856, MET 766, ALA 743, GLY 796, GLY 857	THR 854, GLN 791, ASN 842	LYS745 (multiple H-bonds with oxygen atom), ASN 842 (H-bond with oxygen atom), GLU 762 (H-bond with nitrogen atom)	GLU762 (charged negative), ASP855 (charged negative), LYS745 (charged positive), ARG841 (charged negative)

#### 2.2.2 Molecular docking studies against thymidylate kinase (PDB ID: 4QGG)

The antimicrobial potential of the synthesized derivatives was further explored by docking them against thymidylate kinase (PDB ID: 4QGG). The docking scores of the test compounds ranged between −4.661 and −5.890 kcal mol^−1^. The reference antifungal agent Griseofulvin exhibited a relatively weak binding score (−3.817 kcal mol^−1^), while the antibacterial standard Ciprofloxacin showed the strongest binding affinity (−6.747 kcal mol^−1^).

Among the synthesized ligands, compound 11b (−5.890 kcal mol^−1^) emerged as the most potent binder, surpassing Griseofulvin and approaching Ciprofloxacin. Its strong binding was stabilized by hydrogen bonds with GLN101 and GLU11, along with multiple charged interactions involving ARG70, ARG92, and ARG105. Compound 10f (−5.785 kcal mol^−1^) also demonstrated favorable binding through hydrogen bonding with ARG48 and ARG92, supported by complementary electrostatic contacts with ARG36, LYS15, and GLU37. Similarly, compound 11d (−5.672 kcal mol^−1^) showed stabilizing hydrogen bonds with GLN101 and GLU11, reinforced by hydrophobic contacts with PHE66 and TYR100. Compound 10c (−5.607 kcal mol^−1^) also exhibited good affinity, mediated by hydrogen bonding with GLU37 and GLN101, and stabilization by multiple charged residues.

**Fig. 8:**
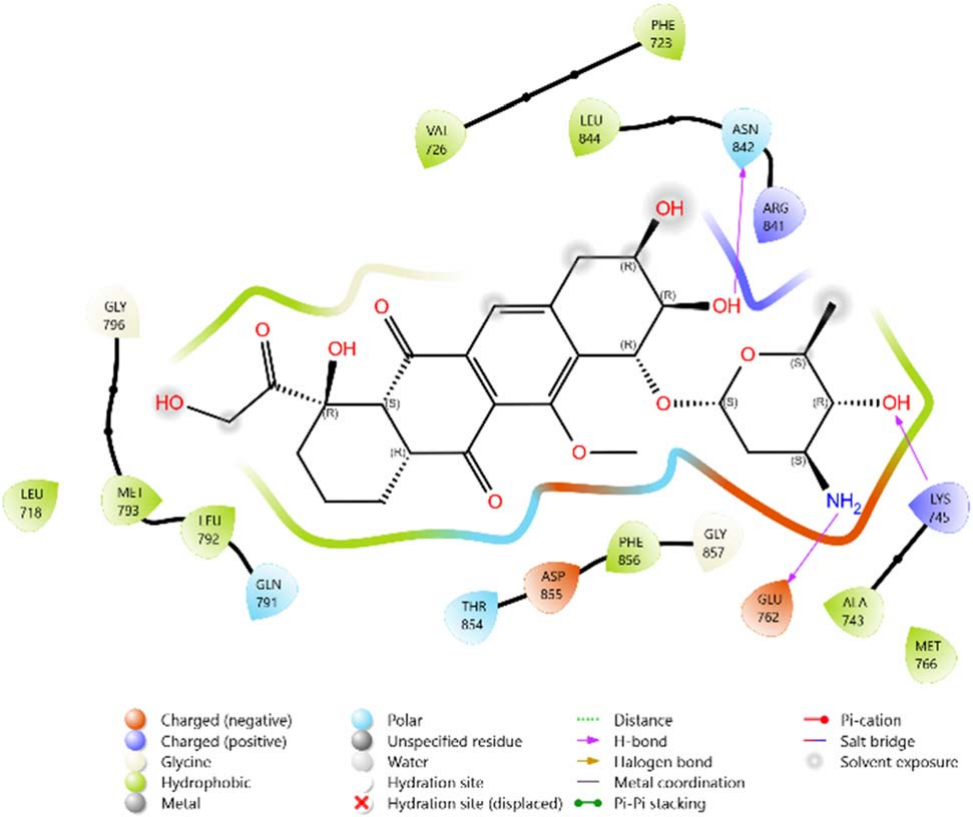
2D diagram of compound 10c docked with the 4QGG protein target.

**Fig. 9:**
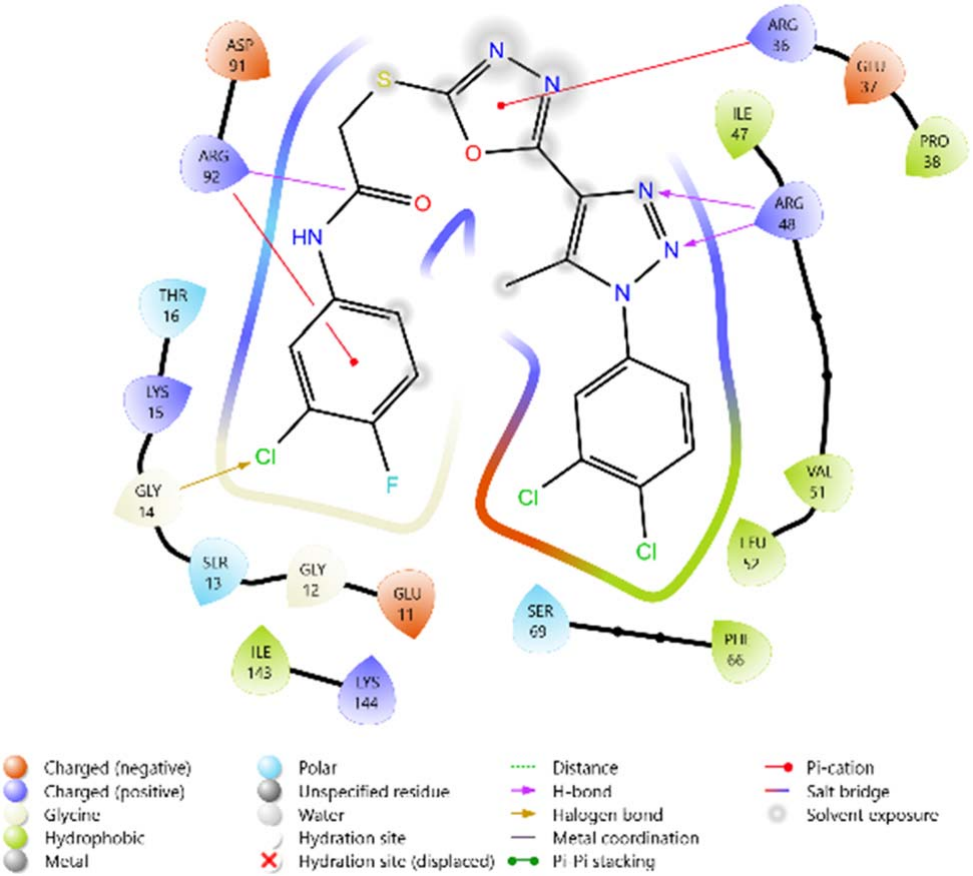
2D diagram of compound 10f docked with the 4QGG protein target.

**Fig. 10:**
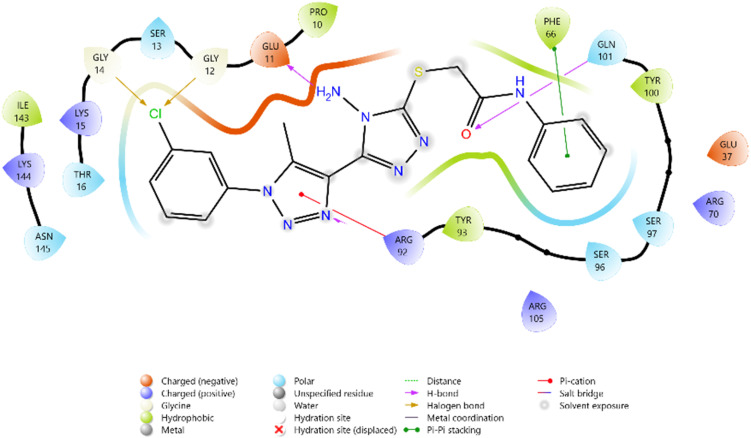
2D diagram of compound 11b docked with the 4QGG protein target.

**Fig. 11:**
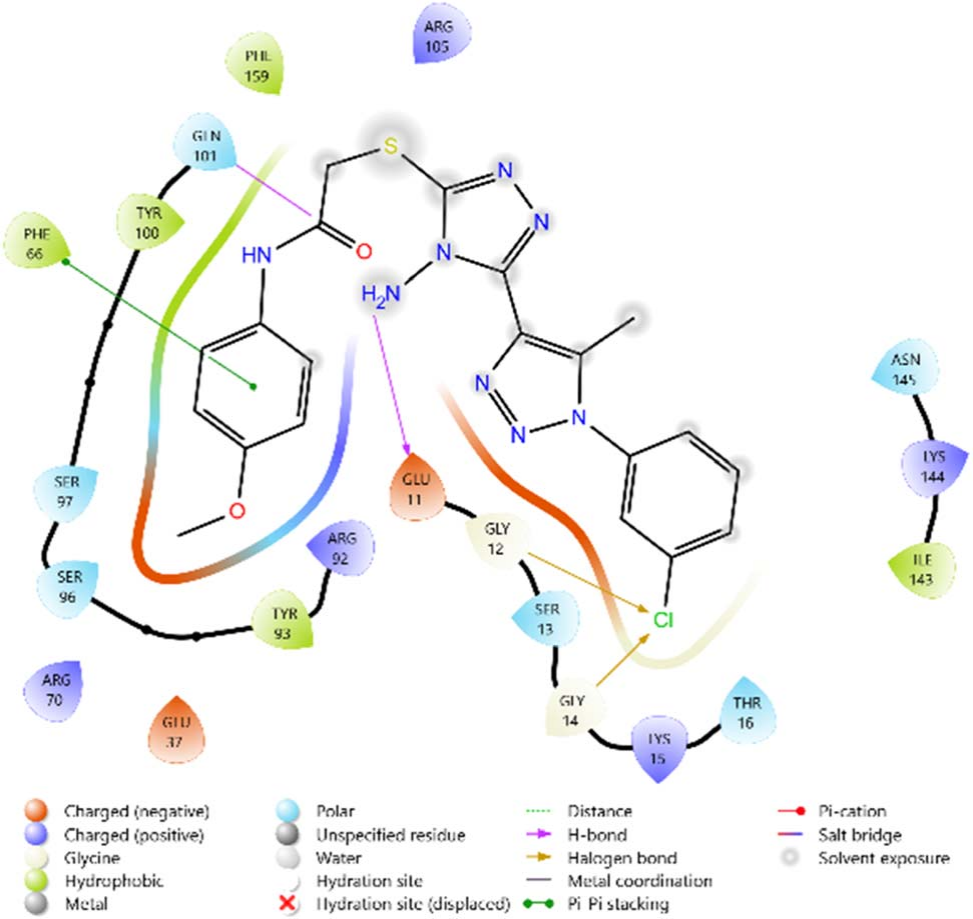
2D diagram of compound 11d docked with the 4QGG protein target.

**Fig. 12:**
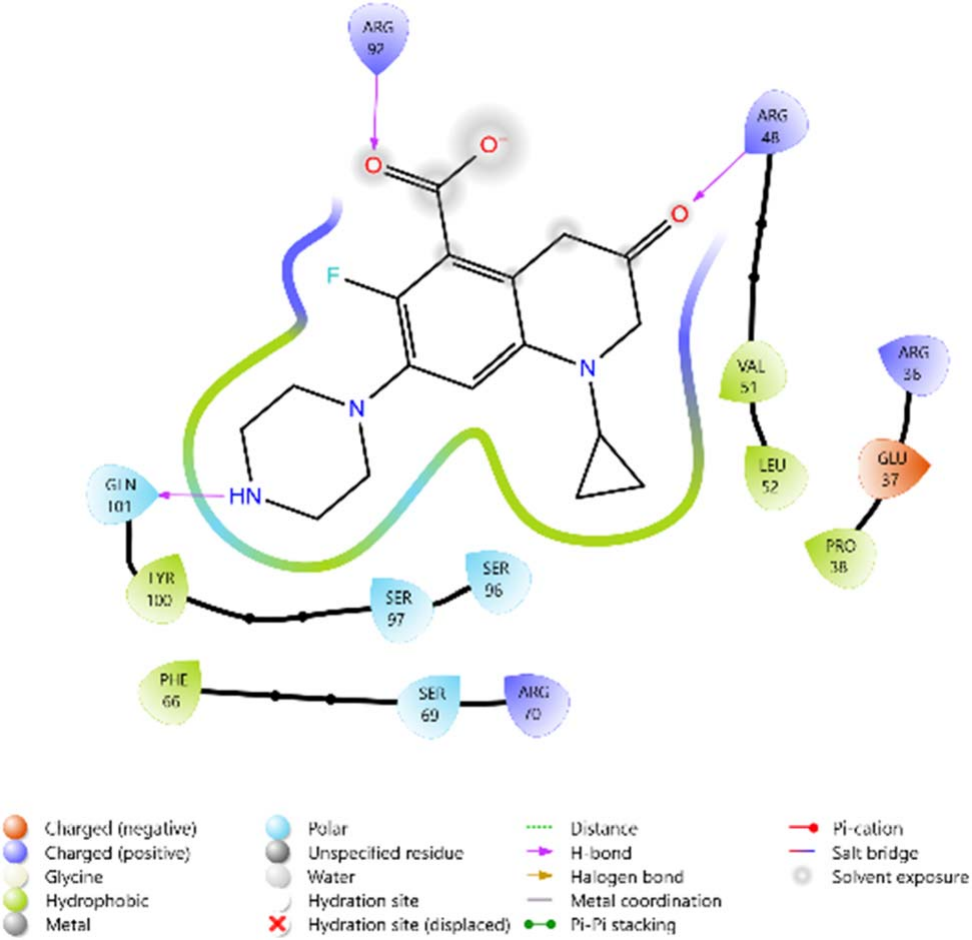
2D diagram of the standard drug Ciprofloxacin docked with the 4QGG protein target.

**Fig. 13:**
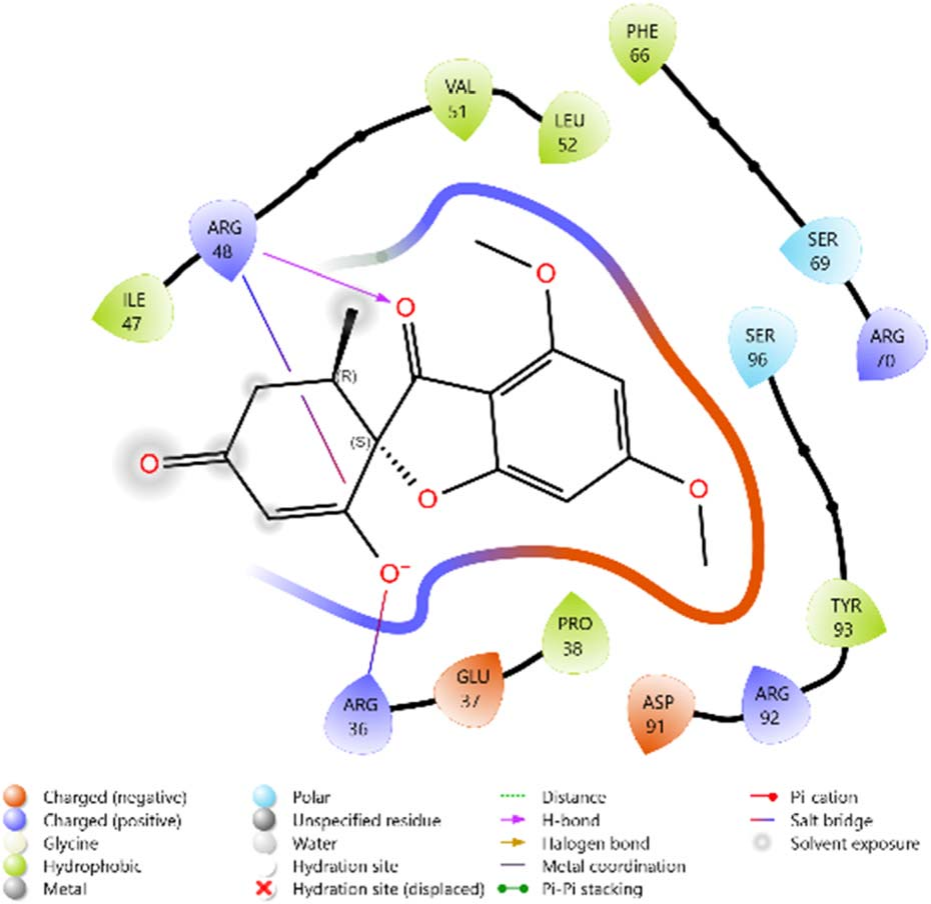
2D diagram of the standard drug Griseofulvin docked with the 4QGG protein target.

The overall binding order was 11b > 10f > 11d > 10c, which indicates that these compounds are significantly more potent binders than Griseofulvin and only moderately weaker than Ciprofloxacin. These findings highlight the potential of these heterocyclic derivatives as promising antimicrobial leads targeting thymidylate kinase (Table 3 and Fig. 8–13).

**Table 3:** Molecular docking analysis of synthesized compounds with thymidylate kinase inhibitor (4QGG)

Compound	Docking score (kcal mol^−1^)	Hydrophobic interactions	Polar interactions	Hydrogen bonds	Other interactions
10c	−5.607	PRO38, VAL51, LEU52, ILE47, PHE66, TYR100, PHE159	SER69, GLN101	GLU37 (H-bond with N); GLN101 (H-bond with N)	ARG36 (charged positive), ARG48 (charged positive), ARG70 (charged positive), ARG105 (charged positive), GLU37 (charged negative), GLU62 (charged negative)
10f	−5.785	ILE47, VAL51, LEU52, PHE66, ILE143, PRO38	SER13, SER69, THR16	ARG48 (H bond with N atom) ARG92 (H-bond with carbonyl C)	ARG36 (charged positive), ARG48 (charged positive), LYS15 (charged positive), LYS144 (charged positive), GLU11 (charged negative), GLU37 (charged negative)
11b	−5.890	PRO10, ILE143, TYR100, TYR93, PHE66	SER13, THR16, SER96, SER97, GLN101, ASN145	GLN101 (H-bond with O atom), GLU11 (H-bond with –NH_2_ atom)	GLU11 (charged negative), GLU37 (charged negative), ARG70 (charged positive), ARG92 (charged positive), ARG105 (charged positive), LYS15 (charged positive), LYS144 (charged positive)
11d	−5.672	ILE143, TYR100, TYR93, PHE66, PHE159	SER13, THR16, SER96, SER97, GLN101, ASN145	GLN101 (H-bond with O atom), GLU11 (H-bond with –NH_2_ atom)	GLU11 (charged negative), GLU37 (charged negative), ARG70 (charged positive), ARG92 (charged positive), LYS15 (charged positive), LYS144 (charged positive), ARG105 (charged positive)
Griseofulvin	−3.817	PRO38, ILE47, VAL51, LEU52, LEU65, TYR93	SER69, SER96	ARG48 (H bond with O atom)	ASP91 (charged negative), GLU37 (charged negative), ARG70 (charged positive), ARG92 (charged positive), ARG36 (charged positive), ARG48 (charged positive)
Ciprofloxacin	−6.747	PRO38, VAL51, LEU52, PHE66, TYR100	SER69, SER96, SER97, GLN101	ARG48 (H bond with O atom), ARG92 (H-bond with O)	GLU37 (charged negative), ARG70 (charged positive), ARG92 (charged positive), ARG36 (charged positive), ARG48 (charged positive)

The docking studies against EGFR (an anticancer target) and thymidylate kinase (an antimicrobial target) reveal that selected triazole-based hybrids exhibit strong binding affinities for these targets. Compounds 10i and 11c emerged as the most promising anticancer agents with superior EGFR interactions, while 11b and 10f showed the highest potential as antimicrobial agents. These results provide molecular-level insights into the multitarget activity of the synthesized derivatives and support their further *in vitro* and *in vivo* evaluation as potential therapeutic candidates.

### 2.3 *In vitro* cytotoxic activity

The synthesized compounds 10a–i and 11a–d were evaluated for their cytotoxic potential against the MCF-7 (human breast adenocarcinoma) and HepG2 (human hepatocellular carcinoma) cell lines using the MTT assay, and the results are summarized in Table 4. The IC_50_ values were compared with pyridyl cyanoguanidine, which served as the reference standard.

**Table 4:** The antitumor activity of the tested compounds expressed as IC50 values and compared with reference standard drugs evaluated on breast and liver cancer cell lines^a^

Comp.	IC50 values (µg mL^−1^) against tumor cell lines	
	MCF-7	HepG2
10a	38.5 ± 0.5	7.4 ± 0.5
10b	1.5 ± 0.5	4.9 ± 0.2
10c	3.7 ± 0.4	49.8 ± 0.6
10d	21.3 ± 0.5	38.9 ± 0.2
10e	52.4 ± 0.2	39.1 ± 0.5
10f	10.1 ± 0.5	43.8
10g	18.4 ± 0.5	8.4 ± 0.5
10h	41.5 ± 0.2	33.1 ± 0.8
10i	1.2 ± 0.5	0.8 ± 0.2
11a	14.1 ± 0.2	40.8 ± 0.7
11b	24.7 ± 0.5	20.2 ± 0.5
11c	28.2 ± 0.5	7.5 ± 0.2
11d	43.5 ± 0.5	44.3 ± 0.2
Pyridyl cyanoguanidine	0.7 ± 0.02	5.1 ± 0.02

a Data were expressed as mean ± SD of three experiments.

Among the 10-series derivatives, compounds 10b (IC_50_ = 16.1 ± 0.5 µg mL^−1^) and 10c (IC_50_ = 6.8 ± 0.4 µg mL^−1^) exhibited moderate to high activity against the MCF-7 cell line, with 10c demonstrating the most pronounced effect, closely approaching that of the standard drug (6.5 ± 0.5 µg mL^−1^). Compounds 10a, 10d, 10f, 10g, and 10i showed only mild cytotoxicity, whereas 10e and 10h recorded IC_50_ values >200 µg mL^−1^, indicating negligible activity.

In the HepG2 assay, 10a (29.1 ± 0.5 µg mL^−1^), 10c (21 ± 0.6 µg mL^−1^), 10e (22.9 ± 0.5 µg mL^−1^), and 10g (39 ± 0.5 µg mL^−1^) were the most effective among the 10-series, though none surpassed the potency of the standard pyridyl cyanoguanidine (5.1 ± 0.2 µg mL^−1^). Notably, 10b, 10f, and 10i were inactive (IC_50_ > 200 µg mL^−1^), suggesting that specific substituents in these molecules may hinder cytotoxicity against liver carcinoma cells.

The 11-series derivatives generally exhibited greater activity than their 10-series counterparts. Compound 11a emerged as the most potent derivative in the entire set, with IC_50_ values of 5.8 ± 0.2 µg mL^−1^ (MCF-7) and 4.2 ± 0.7 µg mL^−1^ (HepG2), surpassing the reference in HepG2 and closely matching it in MCF-7. Compound 11b showed moderate activity against both cell lines (134.9 ± 0.5 µg mL^−1^ in MCF-7 and 36.9 ± 0.5 µg mL^−1^ in HepG2), while 11c displayed only mild activity. Compound 11d was inactive against both lines (IC_50_ > 200 µg mL^−1^).

A comparative SAR analysis suggests that the transformation from the 10-series to the corresponding 11-series, involving the conversion of the 1,2,3-triazole core into a 1,3,4-oxadiazole moiety, resulted in a marked enhancement of cytotoxic activity, particularly in compound 11a. Moreover, the observation of selective activity toward either MCF-7 or HepG2 implies that subtle structural modifications can modulate target specificity. This feature could be strategically exploited in the design of selective anticancer agents.

Overall, compound 11a stands out as a promising lead candidate, with sub-10 µg mL^−1^ IC_50_ values against both cancer cell lines, warranting further investigation into its mechanism of action and *in vivo* efficacy (Table 4).

### 2.4 Antimicrobial activity

The synthesized compounds 10a–i and 11a–d were screened for antibacterial and antifungal activity, and the MIC values are summarized in Table 5. Ciprofloxacin and griseofulvin were used as reference standards for antibacterial and antifungal assays, respectively.

**Table 5:** Minimum inhibitory concentration (MIC; µg mL^−1^) of the most active derivatives^a^

Comp.	MIC (mean ± SEM) (µg mL^−1^)					
	Gram positive bacteria		Gram-negative bacteria		Fungi	
	*S. aureus* ATCC 29213	*B. subtilis* ATCC 6633	*P. aeruginosa* ATCC 27853	*E. coli* ATCC 25922	*A. flavus* ATCC 46283	*C. albicans* ATCC 10231
10a	6.72 ± 0.31	2.96 ± 0.08	3.77 ± 0.03	5.51 ± 0.31	6.31 ± 0.05	9.92 ± 0.12
10b	4.31 ± 0.56	3.63 ± 0.02	4.21 ± 0.06	6.76 ± 0.12	7.90 ± 0.08	6.61 ± 0.21
10c	5.05 ± 0.21	5.90 ± 0.06	4.56 ± 0.05	9.72 ± 0.21	5.56 ± 0.02	5.53 ± 0.33
10d	5.25 ± 0.87	7.24 ± 0.06	6.21 ± 0.02	8.70 ± 0.27	7.21 ± 0.06	11.21 ± 0.15
10e	7.10 ± 0.64	9.61 ± 0.05	5.96 ± 0.01	5.69 ± 0.56	13.10 ± 0.05	13.16 ± 0.25
10f	6.63 ± 0.88	6.31 ± 0.09	7.92 ± 0.05	4.61 ± 0.78	10.90 ± 0.02	8.20 ± 0.20
10g	11.21 ± 0.81	3.23 ± 0.05	8.24 ± 0.05	3.90 ± 0.62	5.21 ± 0.07	6.64 ± 0.40
10h	16.34 ± 1.00	4.51 ± 0.08	4.67 ± 0.08	4.67 ± 0.25	7.89 ± 0.06	5.85 ± 0.16
10i	5.34 ± 0.57	10.23 ± 0.05	5.55 ± 0.06	9.12 ± 0.33	11.58 ± 0.08	19.22 ± 0.26
11a	3.85 ± 0.90	1.89 ± 0.05	2.41 ± 0.02	2.76 ± 0.40	3.31 ± 0.06	4.61 ± 0.35
11b	4.39 ± 0.53	8.56 ± 0.07	9.31 ± 0.08	8.52 ± 0.61	9.21 ± 0.09	7.54 ± 0.64
11c	8.54 ± 0.61	9.41 ± 0.08	16.22 ± 0.07	11.32 ± 0.50	8.54 ± 0.01	10.78 ± 0.42
11d	8.90 ± 0.72	12.50 ± 0.06	6.33 ± 0.08	7.61 ± 0.84	4.34 ± 0.03	12.25 ± 0.29
Ciprofloxacin	5.85 ± 0.13	2.90 ± 0.02	2.90 ± 0.04	2.90 ± 0.25	—	—
Griseofulvin	—	—	—	—	4.25 ± 0.05	12.5 ± 0.15

a SEM = mean of the standard error; each value is the mean of three values.

Among Gram-positive bacteria, 11a emerged as the most potent derivative, with MICs of 3.85 ± 0.90 µg mL^−1^ against *S. aureus* and 1.89 ± 0.05 µg mL^−1^ against *B. subtilis*, surpassing ciprofloxacin (5.85 ± 0.13 and 2.90 ± 0.02 µg mL^−1^, respectively). In the 10-series, notable activity against *B. subtilis* was observed for 10a (2.96 ± 0.08 µg mL^−1^) and 10g (3.23 ± 0.05 µg mL^−1^), both of which were comparable to ciprofloxacin.

In the Gram-negative panel, 11a again displayed the most potent inhibition, with MICs of 2.41 ± 0.02 µg mL^−1^ for *P. aeruginosa* and 2.76 ± 0.40 µg mL^−1^ for *E. coli*, which closely matched those of ciprofloxacin (2.90 ± 0.04 and 2.90 ± 0.25 µg mL^−1^, respectively). Compound 10g also showed good *E. coli* activity (3.90 ± 0.62 µg mL^−1^).

For antifungal activity, 11a showed the lowest MIC against *A. flavus* (3.31 ± 0.06 µg mL^−1^), which was lower than that of griseofulvin (4.25 ± 0.05 µg mL^−1^). Against *C. albicans*, 11a (4.61 ± 0.35 µg mL^−1^) was again the most active, followed by 10h (5.85 ± 0.16 µg mL^−1^) and 10c (5.53 ± 0.33 µg mL^−1^), all superior to griseofulvin (12.5 ± 0.15 µg mL^−1^).

Overall, the conversion from the 1,3,4-oxadiazole (10-series) to the 1,2,3 triazole (11-series) scaffold markedly enhanced antimicrobial potency, with 11a consistently outperforming other derivatives and, in several cases, the standard drugs. These results highlight 11a as a promising broad-spectrum antimicrobial lead (Table 5).

### 2.5 DFT study

#### 2.5.1 Gas phase optimization

**Fig. 14:**
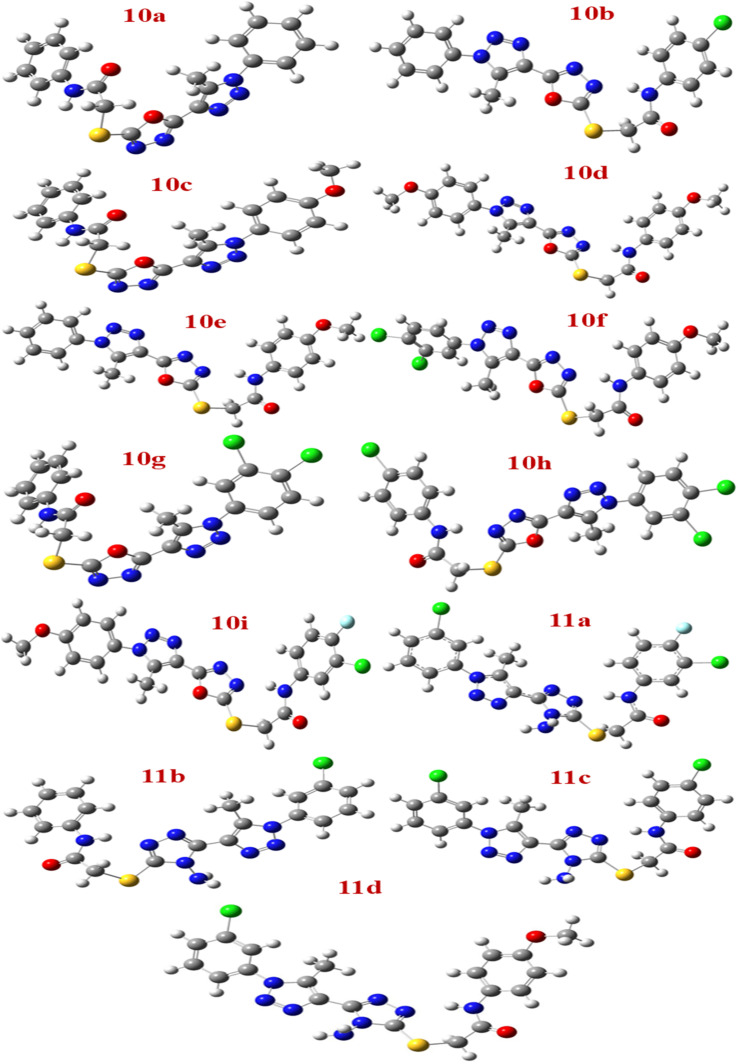
Optimized the structure of molecules 10a to 11d.

The primary chemical structures of all synthesized compounds were first drawn in ChemBioDraw and then converted to three-dimensional geometries for computational analysis. During optimization, all structural parameters, including bond lengths, bond angles, and dihedral angles, were allowed to relax freely until the system reached the minimum-energy conformation. The obtained optimized geometries (Fig. 14) were confirmed as true minima on the potential energy surface by the absence of any imaginary frequencies in subsequent frequency calculations.

#### 2.5.2 Electro density and electrostatic potential

**Fig. 15:**
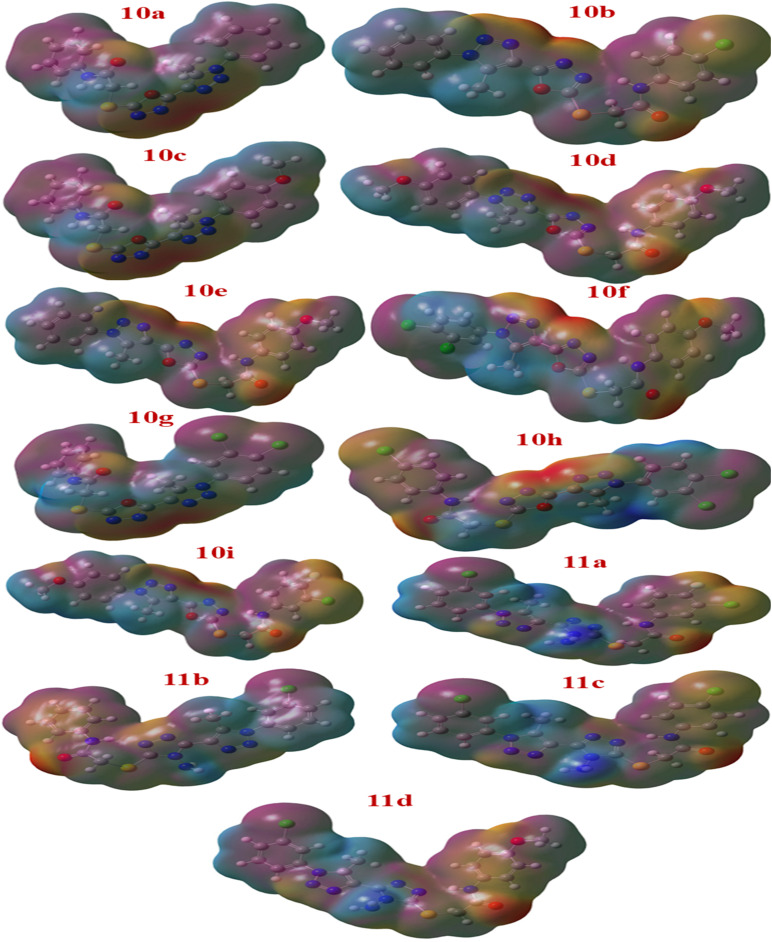
Molecular electrostatic potential (MEP) surface maps of compounds 10a–11d showing electron-rich (red) and electron deficient (purple) regions, highlighting potential reactive sites and charge distribution differences influencing biological activity.

The electrostatic potential (ESP) surface maps of compounds 10a–11d are illustrated in Fig. 15. The ESP analysis provides valuable insight into the molecular charge distribution and plays a crucial role in understanding chemical reactivity, stability, and interactions with biological targets. As shown in Fig. 15, the ESP maps clearly depict the spatial variation in electrostatic potential across the molecular surface, with a color gradient ranging from red (negative potential) to blue (positive potential). Red regions correspond to areas of high electron density and negative electrostatic potential, while blue regions represent areas of electron deficiency and positive electrostatic potential. Intermediate colors such as yellow and green represent regions of moderately negative or neutral potential, respectively.

From the ESP surface analysis, it is evident that oxygen atoms are predominantly surrounded by intense red zones, indicating the most negative electrostatic potential due to oxygen's high electronegativity and strong electron-attracting ability. Nitrogen atoms also display localized negative potential regions, though to a slightly lesser extent compared to oxygen, reflecting their moderate electron-withdrawing nature. In contrast, carbon atoms, especially those in aromatic rings, exhibit blue regions indicating areas of positive electrostatic potential. The halogen atoms present in the structures show weakly negative potential surfaces less negative than oxygen and nitrogen reflecting their intermediate electronegativity and polarizability.

Molecules such as 10i, 10b, and 11c exhibit relatively balanced charge distribution with well-defined nucleophilic and electrophilic regions, suggesting favorable potential for biological interactions *via* hydrogen bonding, π–π stacking, and electrostatic complementarity with target receptors. In contrast, compounds 10d and 11b display more pronounced charge separation between electron-rich heteroatom centers and electron-deficient aromatic carbons, which may contribute to their differential binding modes and biological reactivity profiles.

The ESP analysis thus highlights that electronegative atoms (O and N) serve as potential sites for hydrogen bonding and electrostatic interactions with receptor residues or biomolecular partners. These negatively charged centers can interact effectively with electrophilic or hydrogen-donor sites on biological targets, thereby enhancing binding affinity and complex stability. Conversely, the positively charged regions (primarily around aromatic and aliphatic carbon atoms) are likely to participate in C–H⋯π and π–π interactions, which play significant roles in stabilizing ligand–receptor complexes and improving overall biological activity. The combined visualization of these electrostatic features provides a rational basis for understanding the structure–activity relationships observed in the docking and biological assay results (Fig. 15 & S58).

#### 2.5.3 Frontier molecular orbit

Density Functional Theory (DFT) based quantum chemical calculations were performed to evaluate the electronic characteristics and reactivity of the synthesized molecules (10a–11d). The parameters including HOMO–LUMO energy, energy gap (Δ*E*), ionization potential (*I*), electron affinity (*A*), electronegativity (*χ*), chemical hardness (*η*), softness (*s*), electrophilicity index (*ω*), and chemical reactivity index (Δ*ω*) provide valuable insights into the stability and reactivity behavior of the designed compounds (Tables 6 and 7). The HOMO (Highest Occupied Molecular Orbital) energy represents the electron-donating ability, while the LUMO (Lowest Unoccupied Molecular Orbital) corresponds to the electron-accepting ability. A molecule with a higher (less negative) HOMO value tends to donate electrons more readily, indicating greater nucleophilic character and reactivity. Conversely, a lower (more negative) LUMO energy favors electron acceptance, reflecting electrophilic character. The HOMO values among the studied molecules ranged from −8.03 eV (11b) to −5.45 eV (11d), while LUMO values varied between −2.44 eV (10h) and +2.07 eV (11b). The energy gap (Δ*E* = *E*_LUMO − *E*_HOMO) serves as an essential indicator of molecular stability and chemical reactivity. Molecules with smaller Δ*E* exhibit higher polarizability and are generally more chemically reactive, whereas a larger gap signifies greater stability and lower reactivity. In the present study, the lowest energy gap was observed for compound 10f (3.29 eV), suggesting its superior chemical reactivity, followed closely by 10e (3.63 eV) and 10d (3.71 eV). On the other hand, 11b exhibited the most significant gap (10.10 eV), indicating its high stability and minimal reactivity.^24,25^ The ionization potential (*I*) and electron affinity (*A*) values further corroborate this trend. Compounds with lower ionization potentials are easier to oxidize and tend to participate in charge-transfer interactions. Among the series, 10d (*I* = 5.54 eV) and 10e (*I* = 5.56 eV) had the lowest ionization potentials, indicating their greater tendency to donate electrons. Similarly, 10f (*A* = 2.34 eV) and 10h (*A* = 2.44 eV) exhibited relatively high electron affinity, indicating a strong tendency to accept electrons^26^ (Fig. S55–S57).

**Table 6:** Global quantum chemical parameter of molecule 10a to 10g

	10a	10b	10c	10d	10e	10f	10g
HOMO (eV)	−6.65	−6.03	−6.58	−5.54	−5.56	−5.63	−6.73
LOMO (eV)	−1.69	−1.90	−1.61	−1.82	−1.92	−2.34	−2.10
Energy gap (eV)	4.96	4.12	4.97	3.71	3.63	3.29	4.63
Ionization potential I (eV)	6.65	6.03	6.58	5.54	5.56	5.63	6.73
Electron affinity	1.69	1.90	1.61	1.82	1.92	2.34	2.10
Electro-negativity *χ* (eV)	4.17	3.97	4.09	3.68	3.74	3.98	4.42
Chemical hardness *η* (eV)	2.48	2.06	2.48	1.85	1.81	1.64	2.31
Chemical softness *s* (eV^−1^)	0.40	0.48	0.40	0.53	0.54	0.60	0.43
Chemical potential *µ* (eV)	−4.17	−3.97	−4.09	−3.68	−3.74	−3.98	−4.42
Electrophilicity (eV)	3.51	3.81	3.37	3.65	3.86	4.82	4.21
Δ*N*_max_ (eV)	1.68	1.92	1.64	1.98	2.06	2.42	1.90
Electro donating power *ω*^−^(eV)	11.82	12.12	11.47	11.46	11.92	14.05	13.42
Electro accepting power *ω*^+^ (eV)	3.47	4.18	3.27	4.08	4.43	6.07	4.58
Chemical reactivity index Δ*ω* (eV)	−8.35	−7.94	−8.19	−7.37	−7.49	−7.97	−8.84

**Table 7:** Global quantum chemical parameter of molecule 10h to 11d

	10h	10i	11a	11b	11c	11d
HOMO (eV)	−6.37	−6.46	−6.35	−8.03	−5.89	−5.45
LOMO (eV)	−2.44	−2.03	−2.23	2.07	−1.98	−2.10
Energy gap (eV)	3.93	4.43	4.11	10.10	3.91	3.34
Ionization potential I (eV)	6.37	6.46	6.35	8.03	5.89	5.45
Electron affinity	2.44	2.03	2.23	−2.07	1.98	2.10
Electro-negativity *χ* (eV)	4.40	4.25	4.29	2.97	3.94	3.78
Chemical hardness *η* (eV)	1.96	2.21	2.05	5.05	1.95	1.67
Chemical softness *s* (eV^−1^)	0.50	0.45	0.48	0.19	0.51	0.59
Chemical potential *µ* (eV)	−4.40	−4.25	−4.29	−2.97	−3.94	−3.78
Electrophilicity (eV)	4.93	4.07	4.47	0.87	3.97	4.26
Δ*N*_max_ (eV)	2.24	1.91	2.08	0.58	2.01	2.25
Electro donating power *ω*^−^ (eV)	14.77	12.96	13.76	5.99	12.38	12.73
Electro accepting power *ω*^+^ (eV)	5.96	4.46	5.17	0.04	4.49	5.17
Chemical reactivity index Δ*ω* (eV)	−8.81	−8.50	−8.58	−5.95	−7.88	−7.56

##### 2.5.3.1 Quantum chemical parameter

The chemical hardness (*η*) and softness (*s*) values provide additional understanding of stability and reactivity. A lower hardness (and hence higher softness) corresponds to higher reactivity and ease of electronic excitation. Among all molecules, 10f (*η* = 1.64 eV) exhibited the lowest hardness and the highest softness (*s* = 0.60 eV^−1^), confirming its superior chemical reactivity. This was followed by 10e (*η* = 1.81 eV; *s* = 0.54 eV^−1^) and 11d (*η* = 1.67 eV; *s* = 0.59 eV^−1^). In contrast, 11b (*η* = 5.05 eV) exhibited the highest hardness, suggesting high kinetic stability but poor reactivity. The chemical potential (*µ*) values, which are negative for all compounds, indicate the tendency of the system to lose electrons. The highest magnitude of *µ* was found for 10g (−4.42 eV) and 10h (−4.40 eV), suggesting their strong electron-donating potential. The electronegativity (*χ*) values ranged from 2.97 eV (11b) to 4.42 eV (10g), showing that 10g possesses higher electron-attracting capability compared to others. The electrophilicity index (*ω*) measures the stabilization energy when a molecule gains electrons. The highest electrophilicity was observed for 10f (4.82 eV) and 10h (4.93 eV), indicating that these molecules are excellent electron acceptors and may interact effectively with biological macromolecules through charge-transfer processes. Conversely, 11b (0.87 eV) displayed the lowest electrophilicity, consistent with its high hardness and large energy gap. In addition, the maximum number of electrons that a system can accept (Δ*N*_max_) and electro-donating (*ω*^−^) and electro-accepting powers (*ω*^+^) were analyzed. Higher Δ*N*_max_ indicates greater electron-accepting capacity. Among all, 10f (Δ*N*_max_ = 2.42 eV) and 11d (Δ*N*_max_ = 2.25 eV) showed the highest values, supporting their enhanced electron affinity and reactivity. The electro-donating power (*ω*^−^) followed the trend: 10f (14.05 eV) > 10g (13.42 eV) > 11a (13.76 eV) > 10h (14.77 eV), whereas electro-accepting power (*ω*^+^) was also relatively higher for 10f (6.07 eV) and 10h (5.96 eV), confirming their dual donor–acceptor character favourable for biological interactions. Overall, the combined analysis of frontier orbital energies, reactivity descriptors, and global indices indicates that compound 10f is the most chemically reactive and softest molecule, suggesting its potential for strong interactions with biological targets.

#### 2.5.4 Reduced density gradient (RDG) analysis

**Fig. 16:**
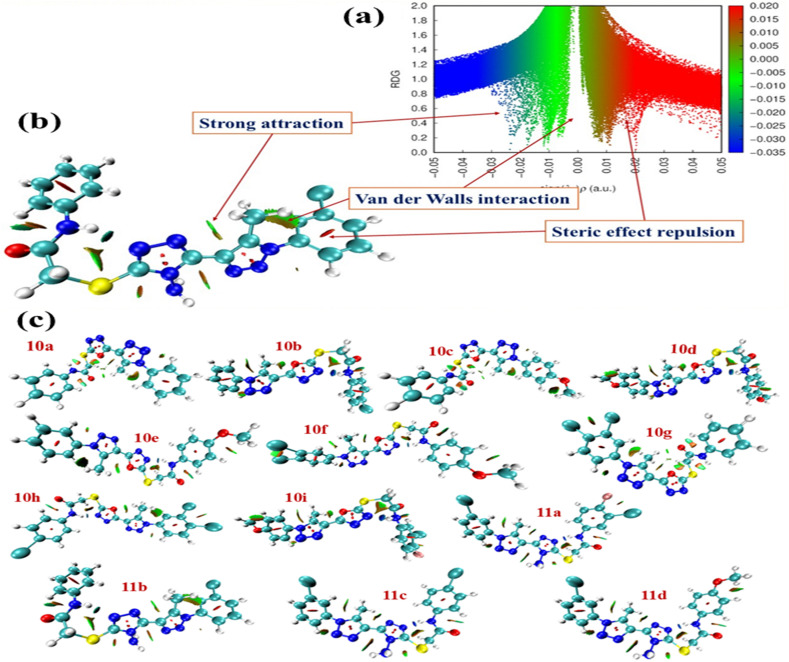
(a and b) Visualization of the reduced density gradient (RDG) isosurface (left) and the corresponding RDG *versus* electron density [sign(*λ*_2_)*ρ*] scatter plot for compound 11b, illustrating the distribution of non-covalent interactions. (c) Comparative RDG isosurface of all synthesized molecules, highlighting variations in attractive, van der Waals, and steric interaction regions.

The reduced density gradient (RDG) analysis of compound 11b, represented as the RDG isosurface mapped against sign(*λ*_2_)*ρ*, provides a comprehensive visualization of the non-covalent interactions present within the molecule. The color-coded RDG isosurface, spanning the range sign(*λ*_2_)*ρ* = −0.035 to +0.020 a.u., distinctly differentiates the nature of these interactions: blue regions (large negative values) correspond to strong attractive (stabilizing) interactions, green regions denote weak dispersion-type or van der Waals (vdW) interactions, and red regions (positive values) represent steric repulsion or close-contact strain. The RDG scatter plot (RDG *vs.* sign(*λ*_2_)*ρ*) displays a pronounced peak near −0.03 a.u., indicating the presence of strong attractive interactions within the molecular framework. On the isosurface, these interactions are visualized as blue or teal patches, primarily localized between the triazole nitrogen atom and neighbouring electron-rich acceptors, most likely the carbonyl oxygen or heterocyclic nitrogen lone pairs. This spatial distribution suggests the existence of intramolecular hydrogen bonding, which contributes to enhanced conformational rigidity and intramolecular stabilization of compound 11b. In addition, green regions are prominently observed across aromatic and heteroaromatic fragments, including phenyl rings and connecting linkers. These widespread green zones correspond to weak van der Waals and π–π or edge-to-face packing interactions between adjacent rings and substituents. Such non-covalent interactions play a crucial role in maintaining the overall structural integrity and are likely to enhance shape complementarity and binding stability during molecular docking with biological targets^27^ (Fig. 16).

Conversely, localized red or orange patches are detected at sterically congested sites, particularly near the junction between the benzoyl fragment and the triazole moiety. These regions represent steric repulsion or close-contact strain, which may restrict specific conformations, influence molecular flexibility, or modulate the preferred binding orientation within target protein pockets. Overall, the RDG analysis highlights a balanced interplay of stabilizing hydrogen-bonding and dispersion forces with localized steric effects, thereby providing a detailed understanding of the non-covalent interaction landscape governing the molecular stability and potential biological activity of compound 11b.^28^

#### 2.5.5 Electron localization function (ELF)

**Fig. 17:**
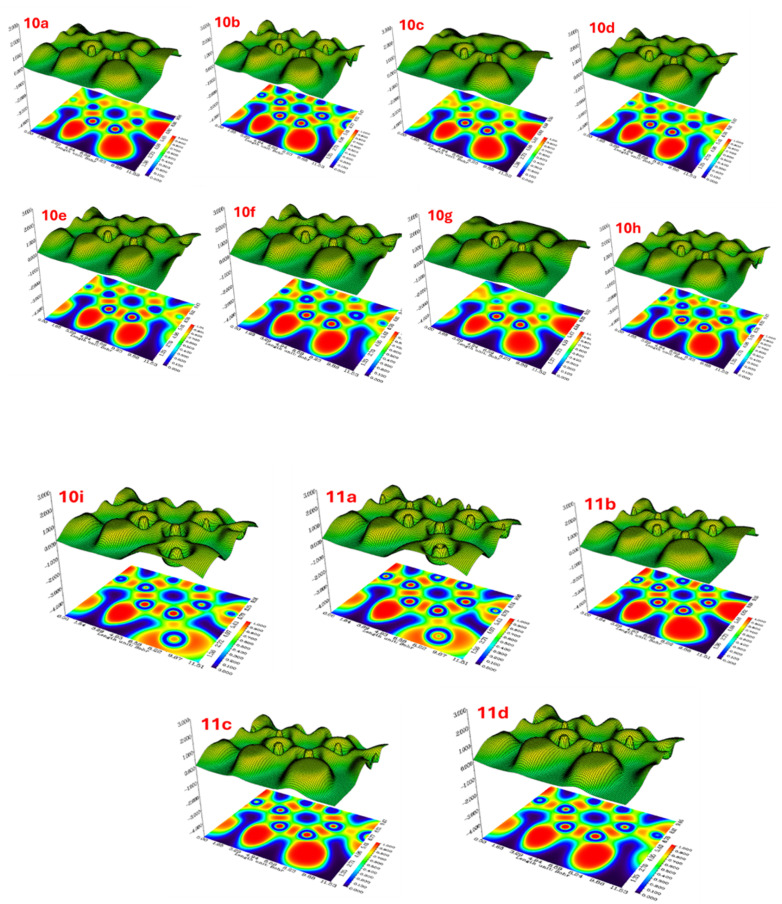
Two-dimensional ELF heatmaps (bottom) and three-dimensional ELF surface plots (top) for synthesized compounds 10a–10i and 11a–11d. The color scale ranges from blue (ELF = 0, low electron localization) to red (ELF = 1.0, high electron localization), indicating regions of covalent bonding, lone pairs, and electron density distribution across the molecular framework 3.6 Mullikan population analysis.

The ELF analysis reveals distinct electronic localization patterns across all thirteen synthesized compounds (10a–10i, 11a–11d). The 2D heatmaps and corresponding 3D surface plots demonstrate regions of high electron localization (red regions, ELF ≈ 1.0) and low localization (blue regions, ELF ≈ 0). Compounds 10a–10d show relatively similar localization patterns with prominent high-density regions indicating strong covalent bonding or lone pair localization. A notable transition is observed in compounds 10e–10h, where the electron density distribution becomes more diffused, suggesting weaker localization and potentially altered bonding characteristics. Compound 10h exhibits notably reduced localization compared to the earlier analogs. The extended series (10i, 11a–11d) displays intermediate localization patterns, with 11a and 11c showing recovery of more defined high-density regions similar to the initial compounds, while 11b and 11d maintain moderate localization profiles. These variations in ELF distributions correlate with structural modifications across the series and provide insights into the electronic nature and chemical reactivity of these compounds.^29^ (Fig. 17).

#### 2.5.6 Localized orbital locator (LOL)

**Fig. 18:**
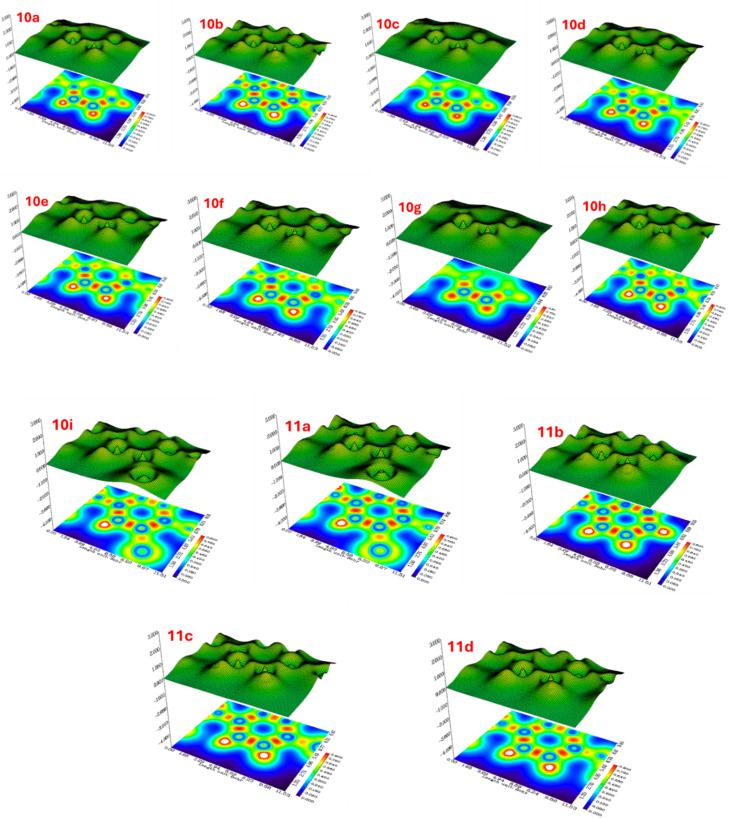
Two-dimensional LOL contour maps (bottom) and three-dimensional LOL surface plots (top) for synthesized compounds 10a–10i and 11a–11d. The color gradient spans from blue (LOL = 0, low localization/delocalized electrons) to red (LOL = 1.0, high localization/localized electron pairs), revealing the spatial distribution of covalent bonds, lone pairs, and regions of electron delocalization within the molecular architecture.

The LOL analysis provides comprehensive insights into the spatial distribution of localized electron pairs across all thirteen synthesized compounds (10a–10i, 11a–11d). The 2D contour maps and 3D surface representations illustrate regions of high orbital localization (red regions, LOL ≈ 1.0) corresponding to covalent bonds and lone pairs. In contrast, low localization zones (blue regions, LOL ≈ 0) indicate delocalized or metallic-like electron behavior. Compounds 10a–10d exhibit well-defined localization patterns with multiple distinct high-density centers, suggesting strong directional bonding character. Progressive structural modifications in compounds 10e–10h result in altered localization profiles, with 10f and 10g showing broader distribution of electron pairs and reduced peak intensities. Notably, 10h displays the most diffuse localization pattern in this subset, indicating significant electronic reorganization. The subsequent series (10i, 11a–11d) demonstrates varied localization characteristics: compounds 10i and 11a maintain moderately strong localization similar to earlier analogs, while 11b exhibits enhanced localization with prominent red zones. Compounds 11c and 11d show intermediate behavior with balanced distribution between localized and delocalized regions. These LOL variations reflect the impact of structural modifications on electron pair localization and provide complementary information to ELF analysis regarding bonding nature and chemical reactivity^30^ (Fig. 18).

#### 2.5.7 Mullikan charge

The Mulliken population analysis provides valuable information about the electronic charge distribution within a molecule and helps identify potential sites for electrophilic and nucleophilic attack. For compound 11b, the calculated Mulliken atomic charges range from −1.059 to +1.060 a.u., indicating a significant degree of charge polarization across the molecular framework. The analysis reveals that nitrogen and oxygen atoms carry the most negative charges (*e.g.*, N7 = −1.059, N17 = −0.963, O9 = −0.603), reflecting their high electronegativity and strong tendency to attract electron density. These atoms are therefore expected to act as nucleophilic centers, favouring interactions with electrophilic regions of biological targets, such as metal ions or proton donors. Conversely, several carbon atoms (C6 = +0.375, C8 = +0.788, C16 = +0.349, C18 = +0.573, and C23 = +0.698) exhibit substantial positive charge density, suggesting their potential role as electrophilic centers in intermolecular interactions. The sulfur atom (S28 = +0.508) also displays a moderately positive charge, which may facilitate its participation in polarization or charge transfer processes. The chlorine atom (Cl30 = +0.122), although less positively charged, exhibits a slight electron-withdrawing character due to its polarizable nature, contributing to the overall dipole moment and molecular polarity.

**Fig. 19:**
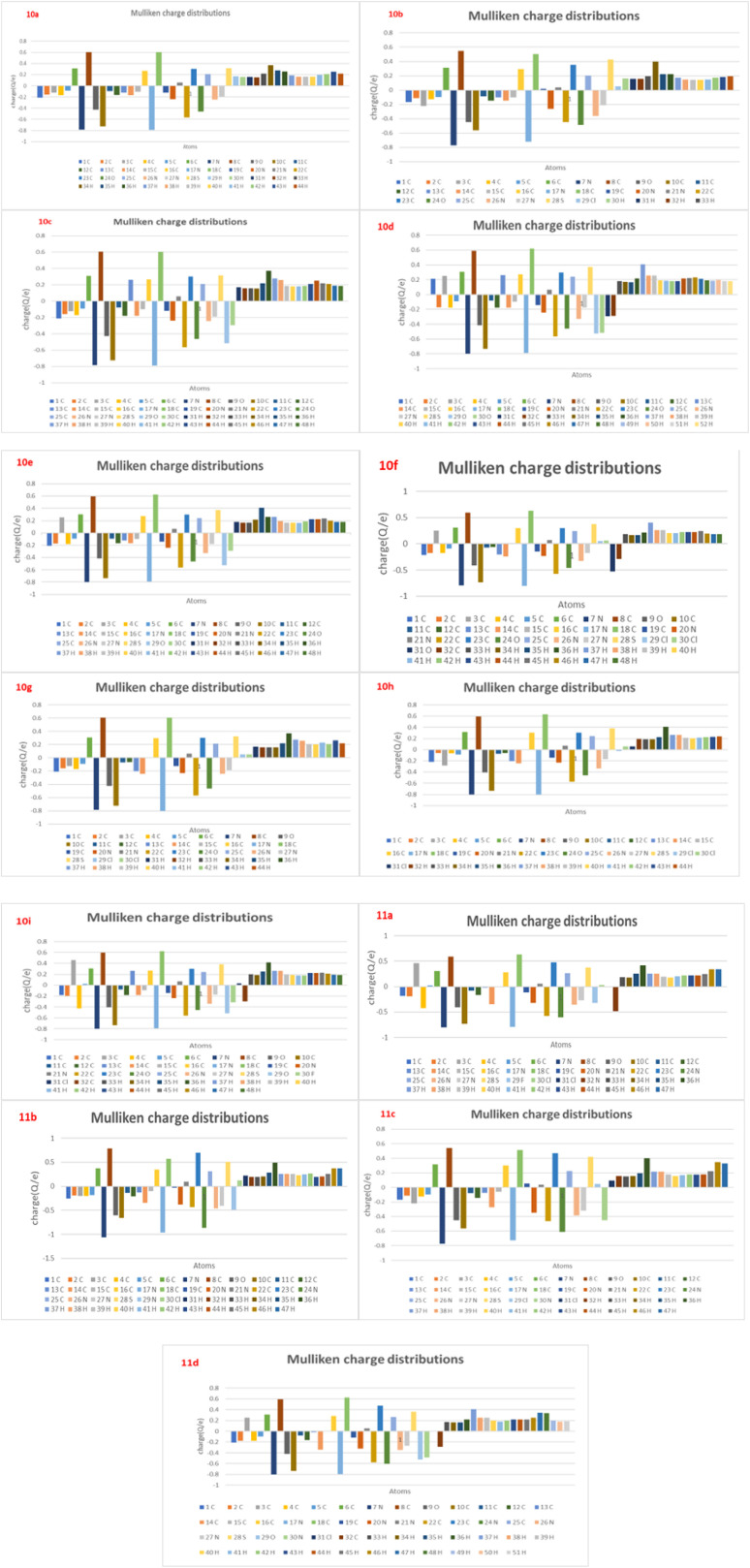
Mulliken atomic charge distributions of the synthesized derivatives 10a–10i and 11a–11d. The bar graphs illustrate the variation in charge density across all atoms within each compound, highlighting the electron-rich and electron-deficient centers responsible for intramolecular charge polarization. Noticeable differences in charge distribution around the heterocyclic core, sulfonamide region, and substituted phenyl rings reflect the influence of different substituents on the electronic environment, supporting their structure–activity relationship and reactivity patterns.

The hydrogen atoms attached to the positively charged carbon centers exhibit partial positive charges ranging from +0.19 to +0.48 a.u., suggesting their potential involvement in C–H⋯π or hydrogen-bonding interactions with nearby electron-rich regions. The uneven charge distribution observed throughout the molecule reflects strong intramolecular charge transfer (ICT), which is often correlated with enhanced chemical reactivity, biological activity, and electronic transition efficiency (Fig. 19).

### 2.6 ADMET and drug-likeness evaluation

**Fig. 20:**
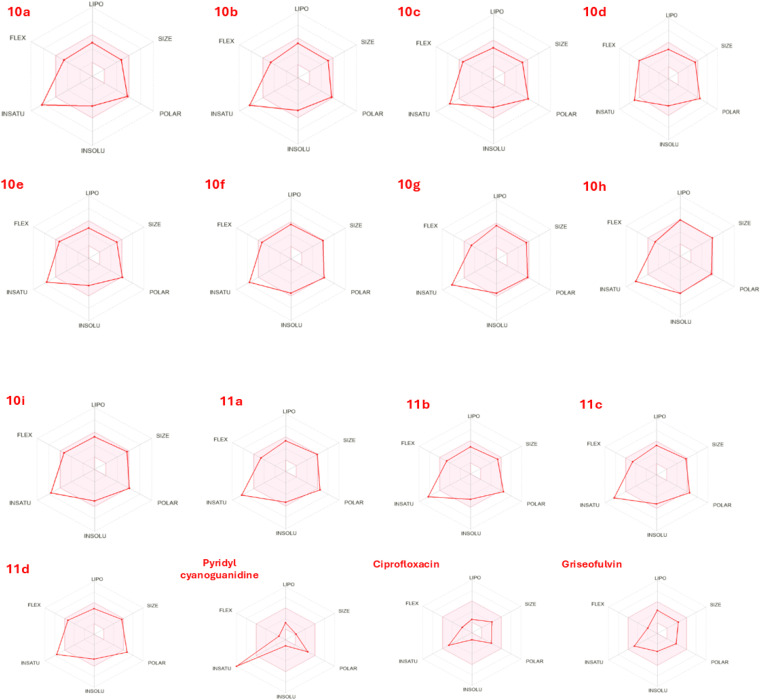
Radar plots of physicochemical properties of compounds 10a–10i and 11a–11d compared with pyridyl cyanoguanidine, ciprofloxacin, and norfloxacin. The plots depict six key parameters (LIPO, SIZE, POLAR, INSOLU, INSATU, FLEX), highlighting overall drug-likeness compliance of the synthesized derivatives.

Pharmacokinetic and pharmacochemical characteristics, along with toxicological profiles, are crucial determinants in the rational design and development of drug-like molecules. In this study, the ADMET parameters of the synthesized compounds were evaluated based on Lipinski's rule of five and Veber's rule, which are essential indicators of oral bioavailability and overall drug-likeness. These rules assess molecular weight (MW ≤ 500), hydrogen bond acceptors (HBA ≤ 10), hydrogen bond donors (HBD ≤5), lipophilicity (*c* log *P* ≤ 5), number of rotatable bonds (*n*_ROTB_ ≤ 10), and topological polar surface area (TPSA ≤ 140 Å^2^), in conjunction with water solubility (log *S*) and percentage absorption (%ABS). As presented in the table, all synthesized derivatives (10a–10i and 11a–11d) complied well with the drug-likeness criteria, except compound 11a, which showed a single Lipinski violation. The compounds exhibited favourable HBD values (1–2) and maintained lipophilicity within the optimal range (2.73–4.42), indicating balanced hydrophilic–lipophilic behavior conducive to effective membrane permeability. The TPSA values (124.03–151.07 Å^2^) remained within or near the recommended threshold, suggesting satisfactory intestinal absorption potential. The calculated log *S* values (−4.3 to −6.07) indicated moderate aqueous solubility consistent with orally active agents. The predicted absorption percentages for the 10 series compounds (59.85–66.21%) were within the therapeutically acceptable range, whereas the 11 series derivatives exhibited slightly lower absorption (56.89–60.07%), likely due to increased hydrogen-bonding capacity. Among all, compound 11b demonstrated the most balanced ADMET profile, with an ideal molecular weight (440.91 g mol^−1^), moderate lipophilicity (*c* log *P* = 2.73), good solubility (log *S* = −4.59), and acceptable absorption (60.07%). Furthermore, the boiled-egg plot confirmed that the majority of compounds fall within the HIA (Human Intestinal Absorption) region, with several also within the BBB (Blood–Brain Barrier) zone, indicating their potential to act as orally bioavailable and pharmacologically effective agents with favourable ADMET characteristics (Table 8 and Fig. 20).

**Table 8:** Predicted pharmacokinetic properties of compounds 10a–i, 11a–d, pyridyl cyanoguanidine, ciprofloxacin, and griseofulvin

Compd	Lipinski's rule					Vebar's rule			
	Lipinski's violations	MW^a^ (≤500)	HBA^b^ (≤10)	HBD^c^ (≤10)	*c* log *P*^d^ (≤5)	*n*_ROTB_ e (≤10)	TPSA^f^ (140 Å^2^)	Log *S*^g^	%ABS^h^
10a	0	392.43	6	1	2.85	7	124.03	−4.3	66.21
10b	0	426.88	6	1	3.28	7	124.03	−4.89	66.21
10c	0	422.46	7	1	2.81	8	133.26	−4.36	63.03
10d	0	452.49	8	1	2.79	9	142.49	−4.42	59.85
10e	0	422.46	7	1	2.78	8	133.26	−4.36	63.03
10f	0	491.35	7	1	3.81	8	133.26	−5.54	63.03
10g	0	461.32	6	1	3.8	7	124.03	−5.48	66.21
10h	0	495.77	6	1	4.42	7	124.03	−6.07	66.21
10i	0	474.9	8	1	3.66	8	133.26	−5.11	63.03
11a	1	493.34	6	2	3.57	7	141.84	−5.35	60.07
11b	0	440.91	5	2	2.73	7	141.84	−4.59	60.07
11c	0	475.35	5	2	3.25	7	141.84	−5.19	60.07
11d	0	470.94	6	2	2.75	8	151.07	−4.66	56.89
Pyridyl cyanoguanidine	0	161.16	3	2	−0.09	2	89.79	−1.15	78.03
Ciprofloxacin	0	331.34	5	2	1.1	3	74.57	−1.32	83.28
Griseofulvin	0	352.77	6	0	2.41	3	71.06	−3.39	84.49

a MW = molecular weight.

b HBA = hydrogen bond acceptor.

c HBD = hydrogen bond donor.

d log *P* = partition coefficient.

e ROTB = rotatable bonds.

f TPSA = total polar surface area.

g Log *S* = aqueous solubility.

h % ABS = percentage of absorption.

## 3. Theoretical analysis

### 3.1 Theoretical platform

All quantum-chemical calculations were performed using Gaussian 09, Revision D.01.^31^ Geometry optimizations and vibrational frequency analyses were carried out at the B3LYP/6-31G(d) level of theory using an UltraFine integration grid (Int = UltraFine; 99 radial shells and 590 angular points) to improve numerical stability. The optimized structures showed no imaginary frequencies, confirming that all stationary points correspond to true minima on the potential energy surface.

Single-point electronic energy calculations were subsequently performed on the optimized geometries using the higher 6-311+G(d,p) basis set to obtain more accurate electronic properties. To better represent the biological environment, solvent effects were incorporated using the SMD implicit solvation model (water) during single-point calculations.

All calculations were performed in the ground state with neutral charge (0) and singlet multiplicity (1). The optimized geometries were further used for molecular orbital analysis, electrostatic potential mapping, and related quantum-chemical descriptors, ensuring a consistent and reliable computational framework throughout the study.^31^

### 3.2 Docking studies

Molecular docking simulations were performed using Schrödinger Suite (Schrödinger, LLC, New York, NY) to evaluate the binding affinity and interaction patterns of synthesized compounds with the target receptors. The three-dimensional crystal structures of the receptors 4QGG and 3W2Q were retrieved from the Protein Data Bank in the RCSB (https://www.rcsb.org/). Protein preparation was conducted using the Protein Preparation Wizard, which included addition of hydrogen atoms, assignment of bond orders, optimization of hydrogen bond networks, and energy minimization using the OPLS4 force field until the root mean square deviation (RMSD) converged to 0.30 Å. Ligand structures were prepared using LigPrep module, generating ionization states at pH 7.0 ± 2.0 and optimizing their geometries. The receptor grid was generated using the Receptor Grid Generation module, with the grid box centered on the active site residues and dimensions set to 20 Å × 20 Å × 20 Å to encompass the entire binding pocket. Molecular docking was performed using Glide module in extra precision (XP) mode. The binding poses were ranked by docking score (binding energy, kcal mol^−1^), and the top-scoring poses were selected for interaction analysis. The interactions between ligands and receptor residues, including hydrogen bonds and hydrophobic contacts, were analyzed and visualized using the Maestro interface.^32^

### 3.3 ADMET parameter

The *in silico* ADMET properties of the synthesized compounds were evaluated using the SwissADME web tool (http://www.swissadme.ch/). SMILES notations of the compounds were generated using ChemDraw software and uploaded to the SwissADME platform.^33^

### 3.4 MTT cytotoxicity assay

MCF-7 (human breast adenocarcinoma) and HepG2 (human hepatocellular carcinoma) cell lines were obtained from the American Type Culture Collection (ATCC, Manassas, VA, USA). The cells were cultured in Dulbecco's Modified Eagle Medium (DMEM) supplemented with 10% fetal bovine serum (FBS) and 1% penicillin–streptomycin antibiotics at 37 °C in a humidified atmosphere containing 5% CO_2_.

The cytotoxic activity of synthesized compounds 10a–i and 11a–d was evaluated using the MTT [3-(4,5-dimethylthiazol-2-yl)-2,5-diphenyltetrazolium bromide] assay. Cells were seeded in 96-well plates at a density of 1 × 10^4^ cells per well and allowed to adhere overnight. The test compounds were dissolved in dimethyl sulfoxide (DMSO) and diluted with culture medium to achieve final concentrations ranging from 1.56 to 200 µg mL^−1^ (final DMSO concentration <0.5%). After 24 hours of incubation, the cells were treated with various concentrations of the test compounds and pyridyl cyanoguanidine (used as a reference standard) and incubated for an additional 48 hours. Subsequently, 20 µL of MTT solution (5 mg mL^−1^ in PBS) was added to each well, and the plates were incubated for 4 hours at 37 °C. The formazan crystals formed were dissolved in 150 µL of DMSO, and the absorbance was measured at 570 nm using a microplate reader. The IC_50_ values (concentration required to inhibit 50% of cell viability) were calculated from dose–response curves using nonlinear regression analysis. All experiments were performed in triplicate, and data are expressed as mean ± standard deviation.^34^

### 3.5 Chemical and characterization

The reagents and solvents used in the present work were obtained from Sigma-Aldrich Chemical Company Inc. and, when needed, further purified. The melting points were measured using an electrically heated apparatus for the determination of single or mixed melting points up to 350 °C, consisting of a cast aluminium case with an enclosed heating block and control apparatus. Infrared spectra were recorded on KBr discs using a Shimadzu FT-IR 8000 spectrophotometer. ^1^H- and ^13^C-NMR spectra were collected at resonance frequencies of 400 MHz and 100 MHz, respectively. NMR spectra were recorded on a Bruker 400 MHz spectrometer using dimethyl sulfoxide (DMSO)-d_6_ as solvent and tetramethylsilane as the internal standard. All chemical shift values (ppm) are reported downfield from TMS, and the coupling constants (*J*) are reported in hertz (Hz). The following standardized abbreviations were used to indicate the splitting pattern (multiplicity): singlet (s), doublet (d), triplet (t), and multiplet (m). Mass spectra were recorded on a Shimadzu LC-MS-8030 mass spectrometer operating at 70 eV. Thin-layer chromatography was carried out using pre-coated silica gel plates (0.25 mm, 60G F254).

## 4. Experimental

### 4.1 Synthesis of various azide derivatives of aniline (3)

For the formation of various azide derivatives, HCl (6 mL) and water (20 mL) were placed in three necked round bottom flask. The solution was cooled at 0 °C. Then aniline derivatives (5.0 g, 0.053 mol) was added drop wise and temperature kept constant between 0–5 °C then sodium nitrite solution (3.65 g, 0.053 mol) and sodium azide (3.44 g, 0.053 mol) was added dropwise at 0–5 °C. Then allow the reaction mass to stirred for 30 min after the completion of reaction the residue extracted using chloroform and washed with water to give 3 (4.2 g, 70.42%).

### 4.2 Synthesis of ethyl 5-methyl-1-phenyl-1*H*-1,2,3-triazole-4-carboxylate derivatives (4)

The formation of ethyl 5-methyl-1-phenyl-1*H*-1,2,3-triazole-4-carboxylate derivatives, azide (4.2 g, 0.035 mol) derivatives treated with ethyl acetoacetate (9.1 g, 0.07 mol) then reaction mixture cooled at 0 °C. Then sodium methoxide (3.78 g, 0.07 mol) was added under an inert atmosphere in ethanol as a solvent. The reaction mixture was stirred at ambient temperature and the progress of the reaction was monitored using TLC. After the completion of reaction, the reaction mass was poured into the ice-cold water, the residue obtained are dry and separated and washed with water and recrystallized from ethanol to give 4 (6.2 g, 76.68%).

### 4.3 Synthesis of 5-methyl-1-phenyl-1*H*-1,2,3-triazole-4-carbohydrazide derivatives (5)

The carbohydrazide derivatives were prepared by dissolving ethyl 5-methyl-1-phenyl-1*H*-1,2,3-triazole-4-carboxylate (6 g, 0.0259 mol) into ethanol (30 mL) and then hydrazine hydrate was added dropwise and the reaction mass was refluxed for 6 h at 80 °C. After the completion of reaction, the reaction mass was cool to room temperature and filtered, the crude product was recrystallized from ethanol (5 g, 88.80%).

### 4.4 Synthesis of 5-(5-methyl-1-phenyl-1*H*-1,2,3-triazol-4-yl)-1,3,4-oxadiazole-2-thiol derivatives (6)

5-methyl-1-phenyl-1*H*-1,2,3-triazole-4-carbohydrazide (2.0 g, 0.009 mol) was refluxed with carbon disulfide (0.76 g, 0.01 mol) and potassium hydroxide (1.40 g, 0.018 mol) in ethanol (15 mL) for 4 hours, and the reaction was followed by TLC until the starting compounds are consumed. The reaction mixture was concentrated and cooled, and the residual solid was dissolved in water (50 mL) and neutralized with 15% HCl (10 mL). The precipitate was filtered off, washed with water, dried, and recrystallized from ethanol (1.72 g, 86.00%).

### 4.5 Synthesis of 4-amino-5-(5-methyl-1-phenyl-1*H*-1,2,3-triazol-4-yl)-4*H*-1,2,4-triazole-3-thiol derivatives (9)

A mixture of 5-(5-methyl-1-phenyl-1*H*-1,2,3-triazol-4-yl)-1,3,4-oxadiazole-2-thiol derivatives (1.0 g, 0.005 mol) and hydrazine hydrate (0.46 g, 0.009 mol) in dry pyridine (10 mL) was refluxed for 2 h. The reaction mixture was then neutralized with dilute HCl under cooling. The solid 9 obtained was crystallized from DMF (0.7 g, 70.00%).

### 4.6 Synthesis of 2-chloro-*N*-phenylacetamide derivatives (8)

2-Chloro-*N*-phenylacetamide derivatives 8 were synthesized by reacting aniline derivatives 7 (1.0 g, 0.01 mol) with chloroacetylchloride (1.13 g, 0.01 mmol), K_2_CO_3_ (1.7 g, 0.012 mol) and DMF (15 mL), the reflux the reaction mass for 6 h at 80 °C after the completion of reaction, the reaction mass was poured into the ice-cold water, the residue obtained are dry and separated and washed with water and recrystallized from ethanol to give 8 (0.81 g, 81.00%).

### 4.7 Synthesis of 2-((5-(5-methyl-1-phenyl-1*H*-1,2,3-triazol-4-yl)-1,3,4-oxadiazol-2-yl)thio)-*N*-phenylacetamide (10a–i)

To a solution of 5-(5-methyl-1-phenyl-1*H*-1,2,3-triazol-4-yl)-1,3,4-oxadiazole-2-thiol derivatives (6) (0.01 mmol) in dimethylformamide (25 mL), potassium carbonate (0.03 mmol) was added gradually under ice-cooling. The reaction solution was stirred for 30 min, and then the appropriate 2-chloro-*N*-phenylacetamide (8) (0.01 mmol) was added portion-wise. The contents were stirred at room temperature for 4–6 h. Then, while constantly stirring, pour onto ice water. Filtration was used to collect the solid, which was then dried and recrystallized from DMF to yield the pure products 10a–i.

### 4.8 Synthesis of 2-((4-amino-5-(5-methyl-1-phenyl-1*H*-1,2,3-triazol-4-yl)-4*H*-1,2,4-triazol-3-yl)thio)-*N*-phenylacetamidethiol (11a–d)

To a solution of 4-amino-5-(5-methyl-1-phenyl-1*H*-1,2,3-triazol-4-yl)-4*H*-1,2,4-triazole-3-thiol derivatives (9) (0.01 mmol) in dimethylformamide (25 mL), potassium carbonate (0.03 mmol) was added gradually under ice-cooling. The reaction solution was stirred for 30 min, and then the appropriate 2-chloro-*N*-phenylacetamide (8) (0.01 mmol) was added portion-wise. The contents were stirred at room temperature for 4–6 h. Then, while constantly stirring, pour onto ice water. Filtration was used to collect the solid, which was then dried and recrystallized from DMF to yield the pure products 11a–d.

### 4.9 Spectral data

#### 4.9.1 2-((5-(5-Methyl-1-phenyl-1*H*-1,2,3-triazol-4-yl)-1,3,4-oxadiazol-2-yl)thio)-*N*-phenylacetamide (10a)

Grey solid; yield 81%; m. p. 202–204 °C; C_19_H_16_N_6_O_2_S. IR (KBr, cm^−1^): 3314 (–NH), 2919 and 2858 (C–H), 1696 (C=O amide), 1673 (C=O oxadiazole), 1603, 1582, 1513, 1490, and 1453 (aromatic C=C, N=N), 1410 and 1318 (C–N), 1269 and 1237 (C–O), 1196 and 1181 (C–N), 1156 and 1117 (aromatic C–H), 1059 and 1036 (C–S, C–N), 882, 824, and 798 (aromatic C–H), 747 (C–S), 541 and 515 cm^−1^ (aromatic ring, N–N). ^1^H NMR (400 MHz, DMSO-d_6_, *δ* ppm): 10.47 (s, 1H, NH), 7.81 (m, 5H, ArH), 7.65 (d, *J* = 8.0 Hz, 2H, ArH), 7.59 (d, *J* = 7.5 Hz, 2H, ArH), 7.54 (s, 1H, ArH), 4.39 (s, 2H, –S–CH_2_–), 2.54 (s, 3H, CH_3_). ^13^C NMR (100 MHz, DMSO-d_6_, *δ* ppm) 168.0 (s), 165.4 (s), 144.3 (s), 137.7 (s), 135.2 (s), 132.3 (s), 129.5 (s), 127.8 (s), 126.3 (s), 125.1 (s), 123.7 (s), 121.3 (s), 34.4 (s), 11.0 (s). ESI-MS (*m*/*z*): 392.1.

#### 4.9.2 *N*-(4-Chlorophenyl)-2-((5-(5-methyl-1-phenyl-1*H*-1,2,3-triazol-4-yl)-1,3,4-oxadiazol-2-yl)thio)acetamide (10b)

Brown solid; yield 83%; m. p. 209–211 °C; C_19_H_15_ClN_6_O_2_S. IR (KBr, cm^−1^): 3431 (–NH), 3275 (aromatic C–H), 3204, 3138, and 3092 (aromatic C–H), 2924 and 2854 (C–H), 1673 (C=O amide), 1601, 1550, and 1509 (aromatic C=C, N=N), 1461, 1436, and 1408 (aromatic C=C), 1373 (C–N), 1332, 1313, and 1280 (C–N, C–O), 1229 (C–O), 1216 and 1165 (C–N), 1114 and 1075 (aromatic C–H, C–S), 959 (aromatic C–H), 822 (C–Cl, aromatic C–H), 743 (C–S), 695, 605, 559, and 512 cm^−1^ (aromatic ring, C–Cl). ^1^H NMR (400 MHz, DMSO-d_6_, *δ* ppm): 10.80 (s, 1H, NH), 7.63–7.69 (m, 5H, ArH), 7.34–7.44 (m, 4H, ArH), 4.40 (s, 2H, –S–CH_2_–), 2.54 (s, 3H, CH_3_). ^13^C NMR (100 MHz, DMSO-d_6_, *δ* ppm) 169.06 (s), 165.49 (s), 145.33 (s), 136.76 (s), 132.27 (s), 131.39 (s), 128.50 (s), 127.00 (s), 124.32 (s), 123.10 (s), 122.72 (s), 120.35 (s), 32.47 (s), 21.06 (s). ESI-MS (*m*/*z*): 427.7.

#### 4.9.3 2-((5-(1-(4-Methoxyphenyl)-5-methyl-1*H*-1,2,3-triazol-4-yl)-1,3,4-oxadiazol-2-yl)thio)-*N*-phenylacetamide (10c)

White solid; yield 87%; m. p. 215–217 °C; C_20_H_18_N_6_O_3_S. IR (KBr, cm^−1^): 3411 (–NH), 3375 (aromatic C–H), 2917, 2831, and 2728 (C–H), 1682 (C=O amide), 1604, 1559, and 1508 (aromatic C=C, N=N), 1394 (C–N), 1323 (C–N), 1239 and 1189 (C–O), 1162 (C–N), 1107 and 1058 (aromatic C–H, C–S), 1028 (C–O–C methoxy), 967 (aromatic C–H), 871 and 819 (aromatic C–H), 772 (C–S), 658, 614, 589, 542, and 511 cm^−1^ (aromatic ring). ^1^H NMR (400 MHz, DMSO-d_6_, *δ* ppm): 10.44 (s, 1H, NH), 7.58–7.60 (t, 4H, ArH), 7.29–7.33 (m, 2H, ArH), 7.08–7.19 (m, 2H, ArH), 7.05–7.08 (m, 1H, ArH), 4.37 (s, 2H, –S–CH_2_–), 3.85 (s, 3H, OCH_3_), 2.53 (s, 3H, CH_3_). ^13^C NMR (100 MHz, DMSO-d_6_, *δ* ppm) 169.6 (s), 168.4(s), 160.5 (s), 150.3 (s), 140 (s), 128.9 (s), 127.8 (s), 126.2 (S),124.9 (S), 123.3 (s), 122.7 (s), 121.5 (s), 115.8 (s), 58.04 (s), 35.7 (s), 10.9 (s). ESI-MS (*m*/*z*): 423.4.

#### 4.9.4 *N*-(4-Methoxyphenyl)-2-((5-(1-(4-methoxyphenyl)-5-methyl-1*H*-1,2,3-triazol-4-yl)-1,3,4-oxadiazol-2-yl)thio)acetamide (10d)

Grey solid; yield 82%; m. p. 200–202 °C; C_21_H_20_N_6_O_4_S. IR (KBr, cm^−1^): 3458 (–NH), 3371 (aromatic C–H), 3043, 2911, 2837, 2798, and 2735 (C–H), 1690 (C=O amide), 1600, 1550, 1532, and 1505 (aromatic C=C, N=N), 1445, 1427, and 1367 (aromatic C=C, C–N), 1307 and 1289 (C–N), 1261, 1214, and 1161 (C–O), 1111 (aromatic C–H, C–S), 1059 (C–O–C methoxy), 1003 and 967 (aromatic C–H), 868 and 833 (aromatic C–H), 757 and 695 (C–S), 644, 579, 552, and 509 cm^−1^ (aromatic ring). ^1^H NMR (400 MHz, DMSO-d_6_, *δ* ppm): 10.73 (s, 1H, NH), 7.57–7.60 (d, *J* = 8.5 Hz, 2H, ArH), 7.51–7.53 (d, *J* = 8.5 Hz, 2H, ArH), 7.24–7.27 (t, *J* = 7.5 Hz, 2H, ArH), 7.12–7.15 (d, *J* = 8.5 Hz, 2H, ArH), 4.09 (s, 2H, –S–CH_2_–), 3.83 (s, 3H, OCH_3_), 3.79 (s, 3H, OCH_3_), 2.55 (s, 3H, CH_3_). ^13^C NMR (100 MHz, DMSO-d_6_, *δ* ppm) 167.6 (s), 167.4 (s), 157.3 (s), 155.4(s), 142.3 (s), 130.39 (s), 130.2 (s), 128.8 (s), 126.91 (s), 126.32 (s), 122.89 (s), 115.5 (s), 113.7 (s), 55.83 (s), 36.4 (s), 16.06 (s). ESI-MS (*m*/*z*): 453.4.

#### 4.9.5 *N*-(4-Methoxyphenyl)-2-((5-(5-methyl-1-phenyl-1*H*-1,2,3-triazol-4-yl)-1,3,4-oxadiazol-2-yl)thio)acetamide (10e)

Grey solid; yield 90%; m. p. 210–212 °C; C_20_H_18_N_6_O_3_S. IR (KBr, cm^−1^): 3300 (–NH), 3202 (aromatic C–H), 2949, 2828, 2723, and 2677 (C–H), 1661 (C=O amide), 1610, 1542, and 1516 (aromatic C=C, N=N), 1452 and 1406 (aromatic C=C, C–N), 1359, 1307, and 1280 (C–N), 1237 (C–O), 1169 (C–N), 1116 and 1087 (aromatic C–H, C–S), 1011 and 976 (C–O–C methoxy), 936, 871, and 824 (aromatic C–H), 680 and 593 (C–S), 534 and 490 cm^−1^ (aromatic ring). ^1^H NMR (400 MHz, DMSO-d_6_, *δ* ppm): 10.78 (s, 1H, NH), 7.63–7.66 (m, 5H, ArH), 7.26–7.28 (d, *J* = 7.5 Hz, 2H, ArH), 7.05–7.07 (m, 2H, ArH), 4.19 (s, 2H, –S–CH_2_–), 3.81 (s, 3H, OCH_3_), 2.49 (s, 3H, CH_3_). ^13^C NMR (100 MHz, DMSO-d_6_, *δ* ppm) ESI-MS (*m*/*z*): 422.2.

#### 4.9.6 2-((5-(1-(3,4-Dichlorophenyl)-5-methyl-1*H*-1,2,3-triazol-4-yl)-1,3,4-oxadiazol-2-yl)thio)-*N*-(4-methoxyphenyl)acetamide (10f)

Brown solid; yield 79%; m. p. 198–200 °C; C_20_H_16_Cl_2_N_6_O_3_S. IR (KBr, cm^−1^): 3445 (–NH), 3271 (aromatic C–H), 3102, 3035, 2924, and 2853 (C–H), 1656 (C=O amide), 1591, 1556, and 1514 (aromatic C=C, N=N), 1460, 1400, and 1344 (aromatic C=C, C–N), 1314, 1296, and 1255 (C–N, C–O), 1234 (C–O), 1193 and 1103 (aromatic C–H, C–S), 1020 (C–O–C methoxy), 985, 879, and 825 (aromatic C–H, C–Cl), 792, 764, and 746 (C–S, C–Cl), 704, 680, 608, and 579 cm^−1^ (aromatic ring, C–Cl). ^1^H NMR (400 MHz, DMSO-d_6_, *δ* ppm): 10.86 (s, 1H, NH), 8.08–8.09 (d, *J* = 2.0 Hz, 1H, ArH), 7.93–7.95 (d, *J* = 8.5 Hz, 1H, ArH), 7.70–7.73 (dd, *J* = 8.5, 2.0 Hz, 1H, ArH), 7.27–7.29 (t, *J* = 7.5 Hz, 2H, ArH), 7.06–7.08 (d, *J* = 8.5 Hz, 2H, ArH), 4.19 (s, 2H, –S–CH_2_–), 3.82 (s, 3H, OCH_3_), 2.53 (s, 3H, CH_3_). ^13^C NMR (100 MHz, DMSO-d_6_, *δ* ppm) 168.3 (s), 165.2 (s), 156.7 (s), 144.6 (s), 135.5 (s), 132.6 (s), 131.5 (s), 129.8 (s), 129.0 (s), 127.5 (s), 126.5 (s), 125.4 (s), 124.6 (s), 122.3 (s), 121.7 (s), 114.9 (s), 114.2 (s), 56.2 (s), 34.7 (s), 11.3 (s). ESI-MS (*m*/*z*): 491.7.

#### 4.9.7 2-((5-(1-(3,4-Dichlorophenyl)-5-methyl-1*H*-1,2,3-triazol-4-yl)-1,3,4-oxadiazol-2-yl)thio)-*N*-phenylacetamide (10g)

White solid; yield 91%; m. p. 190–192 °C; C_19_H_14_Cl_2_N_6_O_2_S. IR (KBr, cm^−1^): 3361 (–NH), 3174 (aromatic C–H), 2924 (C–H), 1648 (C=O amide), 1598, 1550, and 1485 (aromatic C=C, N=N), 1415 (aromatic C=C, C–N), 1289 and 1267 (C–N), 1202 (C–O), 1090 and 1039 (aromatic C–H, C–S), 1007 (C–N), 888 (aromatic C–H, C–Cl), 760 and 717 (C–S, C–Cl), 670, 594, and 448 cm^−1^ (aromatic ring, C–Cl). ^1^H NMR (400 MHz, DMSO-d_6_, *δ* ppm): 10.90 (s, 1H, NH), 8.09 (s, 1H, ArH), 7.93–7.95 (d, *J* = 8.5 Hz, 1H, ArH), 7.70–7.73 (s, 1H, ArH), 7.52–7.56 (d, *J* = 8.5 Hz, 2H, ArH), 7.46 (m, 1H, ArH), 7.37–7.39 (d, *J* = 8.5 Hz, 2H, ArH), 4.22 (s, 2H, –S–CH_2_–), 2.53 (s, 3H, CH_3_). ^13^C NMR (100 MHz, DMSO-d_6_, *δ* ppm) 168.2 (s), 165.7 (s), 156.2 (s), 144.1 (s), 136.7 (s), 132.9 (s), 132.6 (s), 131.8 (s), 131.5 (s), 130.5 (s), 126.5 (s), 124.9 (s), 123.6 (s), 122.3 (s), 121.6 (s), 114.9 (s), 114.1 (s), 56.3 (s), 34.2 (s), 11.2 (s). ESI-MS (*m*/*z*): 462.7.

#### 4.9.8 *N*-(4-Chlorophenyl)-2-((5-(1-(3,4-dichlorophenyl)-5-methyl-1*H*-1,2,3-triazol-4-yl)-1,3,4-oxadiazol-2-yl)thio)acetamide (10h)

White solid; yield 84%; m. p. 194–196 °C; C_19_H_13_Cl_3_N_6_O_2_S. IR (KBr, cm^−1^): 3447 (–NH), 3347 (aromatic C–H), 3147 (aromatic C–H), 2923 and 2853 (C–H), 1647 (C=O amide), 1594, 1548, and 1505 (aromatic C=C, N=N), 1482 and 1420 (aromatic C=C, C–N), 1330 (C–N), 1255 (C–O), 1192 (C–N), 1131 and 1103 (aromatic C–H, C–S), 997 (aromatic C–H), 840 and 809 (C–Cl, aromatic C–H), 765 (C–S, C–Cl), 665, 586, and 471 cm^−1^ (aromatic ring, C–Cl). ^1^H NMR (400 MHz, DMSO-d_6_, *δ* ppm): 10.61 (s, 1H, NH), 8.14 (s, 1H, ArH), 7.96–7.98 (s, 1H, ArH), 7.77 (s, 1H, ArH), 7.60–7.63 (d, *J* = 8.5 Hz, 2H, ArH), 7.38–7.40 (s, 2H, ArH), 4.39 (s, 2H, –S–CH_2_–), 2.51 (s, 3H, CH_3_). ^13^C NMR (100 MHz, DMSO-d_6_, *δ* ppm) 167.9 (s), 165.8 (s), 144.6 (s), 137.5 (s), 135.5 (s), 132.1 (s), 129.8 (s), 127.5 (s), 126.6 (s), 125.4 (s), 123.9 (s), 121.6 (s), 34.7 (s), 11.3 (s). ESI-MS (*m*/*z*): 497.4.

#### 4.9.9 *N*-(3-Chloro-4-fluorophenyl)-2-((5-(1-(4-methoxyphenyl)-5-methyl-1*H*-1,2,3-triazol-4-yl)-1,3,4-oxadiazol-2-yl)thio)acetamide (10i)

Brown solid; yield 82%; m. p. 214–216 °C; C_20_H_16_ClFN_6_O_3_S. IR (KBr, cm^−1^): 3383 (–NH), 3261 (aromatic C–H), 3089, 3052, and 2927 (C–H), 1692 (C=O amide), 1592, 1549, and 1519 (aromatic C=C, N=N), 1484, 1451, and 1414 (aromatic C=C, C–N), 1324 (C–N), 1301, 1256, and 1202 (C–F, C–N), 1150 and 1120 (C–O), 1090 (C–O–C methoxy), 1007 (aromatic C–H, C–S), 904 and 826 (aromatic C–H, C–Cl), 754 (C–S), 703 and 678 (C–Cl, C–F), 587, 632, and 489 cm^−1^ (aromatic ring). ^1^H NMR (400 MHz, DMSO-d_6_, *δ* ppm): 10.79 (s, 1H, NH), 7.74 (s, 1H, ArH), 7.63–7.65 (dd, *J* = 10.0, 2.0 Hz, 1H, ArH), 7.55–7.57 (d, *J* = 8.5 Hz, 2H, ArH), 7.47 (s, 1H, ArH), 7.15–7.18 (d, *J* = 8.5 Hz, 2H, ArH), 4.19 (s, 2H, –S–CH_2_–), 3.84 (s, 3H, OCH_3_), 2.51 (s, 3H, CH_3_). ^13^C NMR (100 MHz, DMSO-d_6_, *δ* ppm) 168.4 (s), 165.2 (s), 144.1 (s), 137.9 (s), 135.1 (s), 132.6 (s), 129.3 (s), 127.6 (s), 126.1 (s), 125.3 (s), 123.5 (s), 121.1 (s), 34.2 (s), 10.9 (s). ESI-MS (*m*/*z*): 474.6.

#### 4.9.10 2-((4-Amino-5-(1-(3-chlorophenyl)-5-methyl-1*H*-1,2,3-triazol-4-yl)-4*H*-1,2,4-triazol-3-yl)thio)-*N*-(3-chloro-4-fluorophenyl)acetamide (11a)

Grey solid; yield 86%; m. p. 211–213 °C; C_19_H_15_Cl_2_FN_8_OS. IR (KBr, cm^−1^): 3456 (–NH, –NH_2_), 3305, 3207, and 3187 (aromatic C–H, –NH_2_), 2998 and 2929 (C–H), 1697 (C=O amide), 1624, 1582, 1546, and 1488 (aromatic C=C, N=N, –NH_2_), 1460 (aromatic C=C, C–N), 1388, 1348, and 1281 (C–N), 1233 (C–F), 1208 (C–N), 1139 (C–O), 1085 (aromatic C–H, C–S), 1006 (aromatic C–H), 880 (C–Cl), 798 (C–S), 720 (C–Cl, C–F), 613, 626, and 469 cm^−1^ (aromatic ring). ^1^H NMR (400 MHz, DMSO-d_6_, *δ* ppm): 10.60 (s, 1H, NH), 7.94 (s, 1H, ArH), 7.72–7.75 (dd, *J* = 10.0, 2.0 Hz, 4H, ArH), 7.37–7.42 (d, *J* = 8.5 Hz, 2H, ArH), 6.30 (s, 2H, NH_2_), 4.18 (s, 2H, –S–CH_2_–), 2.56 (s, 3H, CH_3_). ^13^C NMR (100 MHz, DMSO-d_6_, *δ* ppm) 167.8 (s), 154.6 (s), 149.7 (s), 139.9 (s), 137.6 (s), 134.8 (s), 133.8 (s), 130.9 (s), 126.6 (s), 125.5 (s), 125.3 (s), 122.5 (s), 122.3 (s), 121.3 (s), 116.9 (s), 33.2 (s), 11.2 (s). ESI-MS (*m*/*z*): 493.1.

#### 4.9.11 2-((4-Amino-5-(1-(3-chlorophenyl)-5-methyl-1*H*-1,2,3-triazol-4-yl)-4*H*-1,2,4-triazol-3-yl)thio)-*N*-phenylacetamide (11b)

Grey solid; yield 87%; m. p. 197–199 °C; C_19_H_17_ClN_8_OS. IR (KBr, cm^−1^): 3378 (–NH, –NH_2_), 3295 and 3180 (aromatic C–H, –NH_2_), 2907 (C–H), 1681 (C=O amide), 1628, 1567, 1524, and 1490 (aromatic C=C, N=N, –NH_2_), 1382 and 1332 (aromatic C=C, C–N), 1283, 1233, 1300, and 1278 (C–N), 1173 (C–O), 1147 and 1127 (aromatic C–H, C–S), 912 (aromatic C–H), 840 (C–Cl), 775 and 748 (C–S, C–Cl), 728, 662, and 612 cm^−1^ (aromatic ring). ^1^H NMR (400 MHz, DMSO-d_6_, *δ* ppm): 10.38 (s, 1H, NH), 7.71–7.75 (d, *J* = 7.5 Hz, 4H, ArH), 7.52–7.54 (s, 2H, ArH), 7.30–7.39 (d, *J* = 8.5 Hz, 2H, ArH), 7.05 (s, 1H, ArH), 6.30 (s, 2H, NH_2_), 4.23 (s, 2H, –S–CH_2_–), 2.46 (s, 3H, CH_3_). ^13^C NMR (100 MHz, DMSO-d_6_, *δ* ppm) 168.4 (s), 149.7 (s), 147.4 (s), 146.5 (s), 145.1 (s), 143.3 (s), 140.5 (s), 139.7 (s), 134.0 (s), 133.5 (s), 132.3 (s), 130.9 (s), 130.3 (s), 128.8 (s), 121.1 (s), 35.7 (s), 10.8 (s). ESI-MS (*m*/*z*): 439.6.

#### 4.9.12 2-((4-Amino-5-(1-(3-chlorophenyl)-5-methyl-1*H*-1,2,3-triazol-4-yl)-4*H*-1,2,4-triazol-3-yl)thio)-*N*-(4-chlorophenyl)acetamide (11c)

Brown solid; yield 83%; m. p. 218–220 °C; C_19_H_16_Cl_2_N_8_OS. IR (KBr, cm^−1^): 3548 (–NH, –NH_2_), 3404 and 3319 (aromatic C–H, –NH_2_), 3212, 3176, and 3055 (aromatic C–H), 2975 and 2929 (C–H), 1677 (C=O amide), 1629, 1600, and 1597 (aromatic C=C, N=N, –NH_2_), 1373 (aromatic C=C, C–N), 1293, 1232, and 1207 (C–N), 1163 (C–O), 1114 and 1049 (aromatic C–H, C–S), 933 (aromatic C–H), 912 and 880 (C–Cl), 778 (C–S, C–Cl), 696, 659, 613, 558, and 532 cm^−1^ (aromatic ring, C–Cl). ^1^H NMR (400 MHz, DMSO-d_6_, *δ* ppm): 10.52 (s, 1H, NH), 7.75 (s, 4H, ArH), 7.62–7.64 (d, *J* = 8.5 Hz, 2H, ArH), 7.37–7.39 (d, *J* = 8.0 Hz, 2H, ArH), 6.30 (s, 2H, NH_2_), 4.20 (s, 2H, –S–CH_2_–), 2.56 (s, 3H, CH_3_). ^13^C NMR (100 MHz, DMSO-d_6_, *δ* ppm) 168.3 (s), 149.2 (s), 139.9 (s), 137.6 (s), 136.8 (s), 133.8 (s), 130.9 (s), 129.6 (s), 129.0 (s), 128.9 (s), 126.1 (s), 125.8 (s), 122.4 (s), 33.2 (s), 14.3 (s). ESI-MS (*m*/*z*): 474.6.

#### 4.9.13 2-((4-Amino-5-(1-(3-chlorophenyl)-5-methyl-1*H*-1,2,3-triazol-4-yl)-4*H*-1,2,4-triazol-3-yl)thio)-*N*-(4-methoxyphenyl)acetamide (11d)

Grey solid; yield 81%; m. p. 189–191 °C; C_20_H_19_ClN_8_O_2_S. IR (KBr, cm^−1^): 3369 (–NH, –NH_2_), 3262 and 3191 (aromatic C–H, –NH_2_), 2918 (C–H), 1683 (C=O amide), 1625, 1572, 1514, and 1460 (aromatic C=C, N=N, –NH_2_), 1432 and 1376 (aromatic C=C, C–N), 1313, 1295, and 1264 (C–N, C–O), 1197, 1170, and 1119 (C–O), 1074 (C–O–C methoxy), 1146 and 1009 (aromatic C–H, C–S), 975 (aromatic C–H), 876 and 840 (C–Cl), 788 and 742 (C–S, C–Cl), 689, 652, 606, 570, 545, and 510 cm^−1^ (aromatic ring). ^1^H NMR (400 MHz, DMSO-d_6_, *δ* ppm): 10.23 (s, 1H, NH), 7.52 (s, 4H, ArH), 7.50 (s, 2H, ArH), 6.88–6.91 (d, *J* = 8.5 Hz, 2H, ArH), 6.30 (s, 2H, NH_2_), 4.16 (s, 2H, –S–CH_2_–), 3.73 (s, 3H, OCH_3_), 2.60 (s, 3H, CH_3_). ^13^C NMR (100 MHz, DMSO-d_6_, *δ* ppm) 169.2 (s), 157.7 (s), 150.6 (s), 140.4 (s), 139.6 (s), 135.3 (s), 132.5 (s), 130.5 (s), 126.5 (s), 126.8 (s), 123.3 (s), 115.9 (s), 114.1 (s), 56.3 (s), 32.7 (s), 13.2 (s). ESI-MS (*m*/*z*): 469.5.

## 5. Conclusion

In this study, a novel series of heterocyclic derivatives (10a–i, 11a–d) was synthesized and evaluated through integrated DFT, molecular docking, and biological assays. Computational analyses identified 11b, 10f, 10i, 11c, and 10d as highly reactive compounds with strong binding affinities toward Thymidylate Kinase (4QGG) and EGFR (3W2Q). Compound 11b exhibited the best antimicrobial docking score (−5.89 kcal mol^−1^), while 10i and 11c showed excellent EGFR binding. Biological evaluations validated these predictions. Compound 10i demonstrated exceptional cytotoxicity activity with IC_50_ values of 1.2 ± 0.5 µM (MCF-7) and 0.8 ± 0.2 µM (HepG2), alongside significant EGFR inhibition (IC_50_ = 0.93 ± 0.25 µM). Compound 11c exhibited superior EGFR inhibitory activity (IC_50_ = 0.33 ± 0.06 µM), approaching erlotinib's potency. Additionally, 11b displayed potent broad-spectrum antimicrobial activity (MIC = 1.89–4.61 µg mL^−1^), outperforming standard drugs. The nitrogen- and oxygen-rich heteroaromatic frameworks enhanced molecular reactivity and target binding. Compounds 10i, 11b, and 11c emerge as promising lead candidates with cytotoxicity and antimicrobial efficacy, providing a robust foundation for future *in vivo* studies and structural optimization toward next-generation multifunctional therapeutics.

## Conflicts of interest

There are no conflicts to declare.

## Abbreviations

EGFR: epidermal growth factor receptor
MIC: minimum inhibitory concentration
HOMO: highest occupied molecular orbital
LUMO: lowest unoccupied molecular orbital
RMSD: root mean square deviation

## Supplementary Material

RA-016-D5RA09082B-s001

## Data Availability

Additional raw data can be made available from the corresponding author upon reasonable request. All data supporting the findings of this study, including NMR, FT-IR, Mass spectra, computational DFT files, molecular docking results, and ADME analysis, are provided within the manuscript and the supplementary information (SI). Supplementary information is available. See DOI: https://doi.org/10.1039/d5ra09082b.
