# Supplementary material for: Synthesis, spectroscopic characterization, and DFT-assisted molecular docking analysis of novel 1,3,4-oxadiazole–1,2,3-triazole hybrids with antimicrobial and cytotoxicity potential
Source: RSC Adv. 2026 Jan 30;16(7):6314–37. doi: 10.1039/d5ra09082b (PMC12856985; doi:10.1039/d5ra09082b)
Supplement: RA-016-D5RA09082B-s001 [file RA-016-D5RA09082B-s001.pdf]

Supplementary Information (SI) for RSC Advances.
This journal is © The Royal Society of Chemistry 2026

Synthesis, Spectroscopic Characterization, and DFT-Assisted Molecular Docking Analysis of Novel 1,3,4-Oxadiazole - 1,2,3-Triazole Hybrids with Antimicrobial and Cytotoxicity Potential

Darshna K. Lakhnotra[a,b], Jay B. Maheta[a], Nargis H. Shaikh[a], Prince A. Dave[a], Yogesh O. Bhola[a*], Bhavesh N. Socha[c], Suresh B. Koradiya[a]

[a] Department of Chemistry, Shri M.P. Pandya Science College-Lunawada, Mahisagar, Shri Govind Guru University-Godhra, Gujarat, India.

[b] Department of Chemistry, Mahisagar Science College-Lunawada, Mahisagar, Shri Govind Guru University-Godhra, Gujarat, India.

[c]Department of Materials Science, Sardar Patel University, Vallabh Vidyanagar-388120, Gujarat, India

Supporting Information

Contain
**Fig. S1:** ^{1}H NMR spectra of final compound 10a
**Fig. S2:** ^{1}H NMR spectra of final compound 10b
**Fig. S3:** ^{1}H NMR spectra of final compound 10c
**Fig. S4:** ^{1}H NMR spectra of final compound 10d
**Fig. S5:** ^{1}H NMR spectra of final compound 10e
**Fig. S6:** ^{1}H NMR spectra of final compound 10f
**Fig. S7:** ^{1}H NMR spectra of final compound 10g
**Fig. S8:** ^{1}H NMR spectra of final compound 10h
**Fig. S9:** ^{1}H NMR spectra of final compound 10i
**Fig. S10:** ^{1}H NMR spectra of final compound 11a
**Fig. S11:** ^{1}H NMR spectra of final compound 11b
**Fig. S12:** ^{1}H NMR spectra of final compound 11c
**Fig. S13:** ^{1}H NMR spectra of final compound 11d
**Fig. S14:** Mass spectra of final compound 10a
**Fig. S15**: Mass spectra of final compound 10b
**Fig. S16:** Mass spectra of final compound 10c
**Fig. S17**: Mass spectra of final compound 10d
**Fig. S18**: Mass spectra of final compound 10e

**Fig. S19**: Mass spectra of final compound 10f
**Fig. S20**: Mass spectra of final compound 10g
**Fig. S21**: Mass spectra of final compound 10h
**Fig. S22**: Mass spectra of final compound 10i
**Fig. S23**: Mass spectra of final compound 11a
**Fig. S24**: Mass spectra of final compound 11b
**Fig. S25**: Mass spectra of final compound 11c
**Fig. S26**: Mass spectra of final compound 11d
**Fig. S27:** IR spectra of final compound 10a
**Fig. S28:** IR spectra of final compound 10b
**Fig. S29:** IR spectra of final compound 10c
**Fig. S30:** IR spectra of final compound 10d
**Fig. S31:** IR spectra of final compound 10e
**Fig. S32:** IR spectra of final compound 10f
**Fig. S33:** IR spectra of final compound 10g
**Fig. S34:** IR spectra of final compound 10h
**Fig. S35:** IR spectra of final compound 10i
**Fig. S36:** IR spectra of final compound 11a
**Fig. S37:** IR spectra of final compound 11b
**Fig. S38:** IR spectra of final compound 11c
**Fig. S39:** IR spectra of final compound 11d
**Fig. S40:** ^{13}C NMR spectra of final compound 10a
**Fig. S41:** ^{13}C NMR spectra of final compound 10b
**Fig. S42:** ^{13}C NMR spectra of final compound 10c
**Fig. S43:** ^{13}C NMR spectra of final compound 10d
**Fig. S44:** ^{13}C NMR spectra of final compound 10e
**Fig. S45:** ^{13}C NMR spectra of final compound 10f
**Fig. S46:** ^{13}C NMR spectra of final compound 10g
**Fig. S47:** ^{13}C NMR spectra of final compound 10h
**Fig. S48:** ^{13}C NMR spectra of final compound 10i
**Fig. S49:** ^{13}C NMR spectra of final compound 11a
**Fig. S50:** ^{13}C NMR spectra of final compound 11b
**Fig. S51:** ^{13}C NMR spectra of final compound 11c
**Fig. S52:** ^{13}C NMR spectra of final compound 11d
**Table S1:** Minimum inhibitory concentration (MIC; µg/mL) of the most active derivatives [b]
**Table S2.** The antitumor activities of the tested compounds expressed as IC50 values and compared with reference standard drugs evaluated on breast and liver cancer cell lines [a]
**Table S3:** Molecular docking results of synthesized pyrazole-oxadiazole hybrids (10a-i, 11a-d) and

reference drugs (Erlotinib and Doxorubicin) against EGFR kinase domain (PDB: 3W2Q), displaying binding affinity scores and critical amino acid interactions.
**Figure S53.** 2D and 3D Molecular docking diagram 3W2Q
**Table S4:** Molecular docking results of synthesized pyrazole-oxadiazole hybrids (10a-i, 11a-d) and reference drugs (Ciprofloxacin and Griseofulvin) against EGFR kinase domain (PDB: 4QGG), displaying binding affinity scores and critical amino acid interactions.
**Figure S54.** 2D and 3D Molecular docking diagram 4QGG
**Table S5.** Mulliken Atomic Charges of Selected Atoms in the Optimized Structure (Compound 10a)
**Table S6.** Mulliken Atomic Charges of Selected Atoms in the Optimized Structure (Compound 10b)
**Table S7.** Mulliken Atomic Charges of Selected Atoms in the Optimized Structure (Compound 10c)
**Table S8.** Mulliken Atomic Charges of Selected Atoms in the Optimized Structure (Compound 10d)
**Table S9.** Mulliken Atomic Charges of Selected Atoms in the Optimized Structure (Compound 10e)
**Table S10.** Mulliken Atomic Charges of Selected Atoms in the Optimized Structure (Compound 10f)
**Table S11.** Mulliken Atomic Charges of Selected Atoms in the Optimized Structure (Compound 10g)
**Table S12.** Mulliken Atomic Charges of Selected Atoms in the Optimized Structure (Compound 10h)
**Table S13.** Mulliken Atomic Charges of Selected Atoms in the Optimized Structure (Compound 10i)
**Table S14.** Mulliken Atomic Charges of Selected Atoms in the Optimized Structure (Compound 11a)
**Table S15.** Mulliken Atomic Charges of Selected Atoms in the Optimized Structure (Compound 11b)
**Table S16.** Mulliken Atomic Charges of Selected Atoms in the Optimized Structure (Compound 11c)
**Table S17.** Mulliken Atomic Charges of Selected Atoms in the Optimized Structure (Compound 11d)
**Figure S55.** HOMO–LUMO energy level distribution and energy gap (ΔE) diagrams of compounds 10a–10d, showing electron density localization in frontier molecular orbitals. The visualized HOMO and LUMO surfaces reveal charge-transfer regions, highlighting the electronic transitions that govern molecular reactivity and stability.
**Figure S56.** HOMO–LUMO energy level distribution and energy gap (ΔE) diagrams of compounds 10e–10h, illustrating the spatial electron density distribution in the frontier molecular orbitals. The visualization highlights intramolecular charge-transfer pathways that influence the electronic properties, reactivity, and stability of the compounds.
**Figure S57:** HOMO-LUMO electron density clouds and energy gaps (ΔE) for compounds 10i-11d, showing charge distribution and reactivity differences.
**Figure S58 :** 10a-I &11a-d ESP

Fig. S1: ^{1}H NMR spectra of final compound 10a

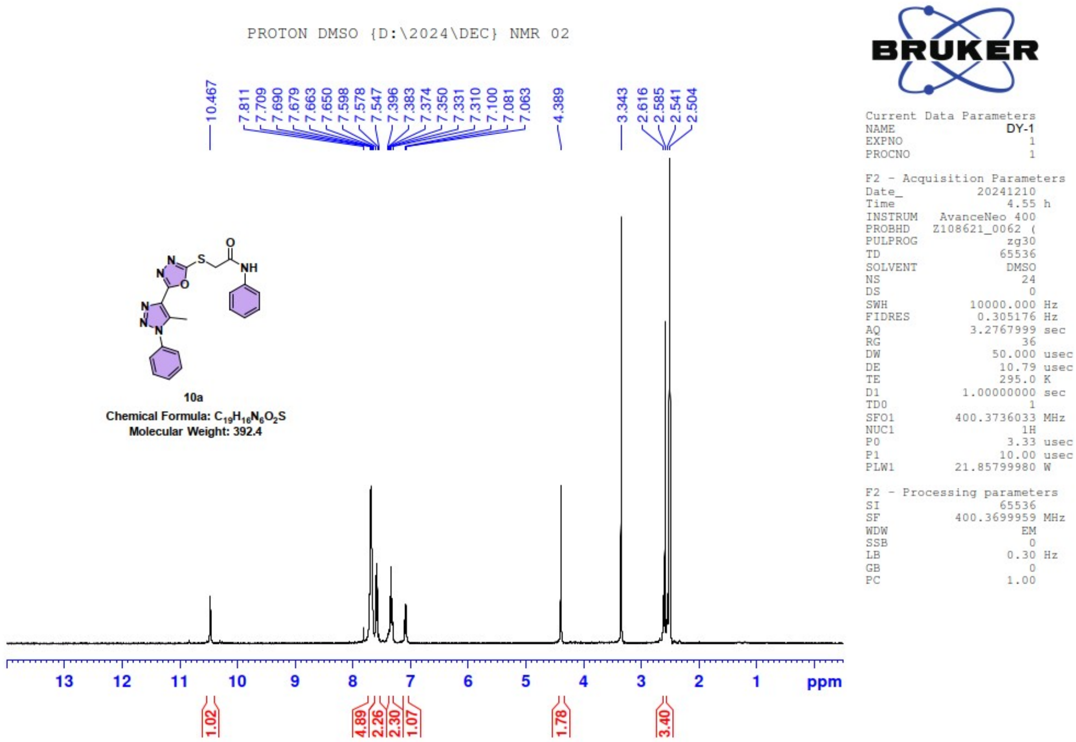

Fig. S2: ^{1}H NMR spectra of final compound 10b

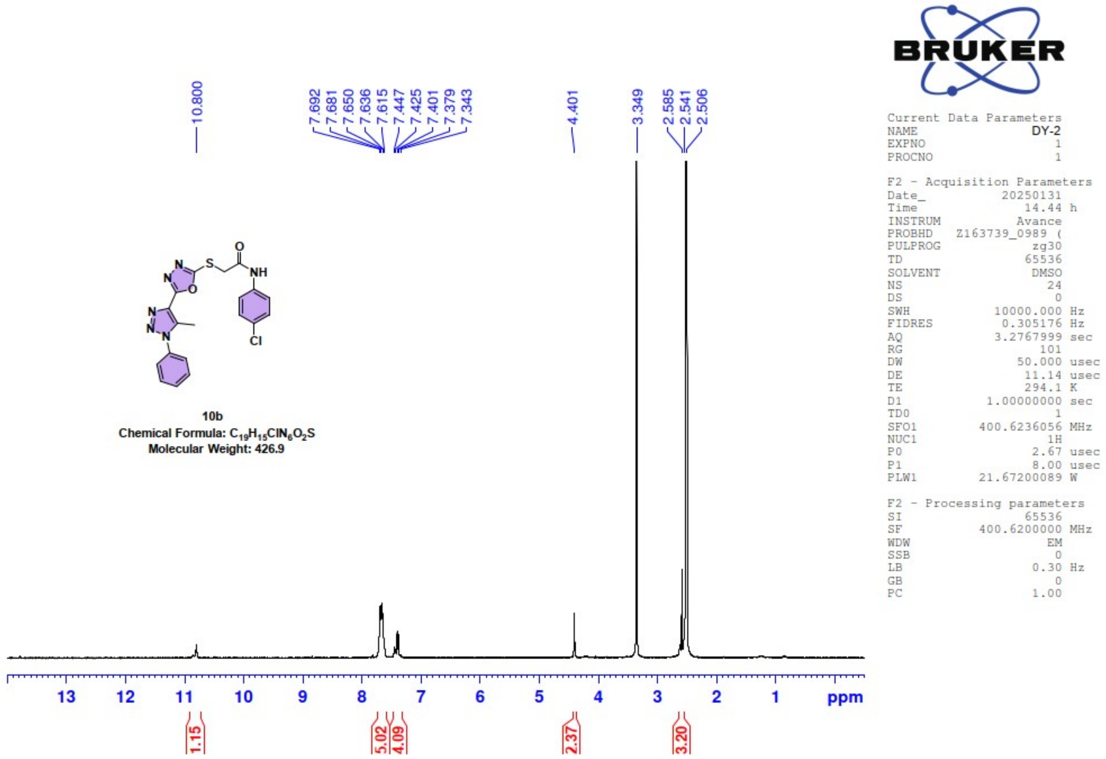

Fig. S3: ^{1}H NMR spectra of final compound 10c

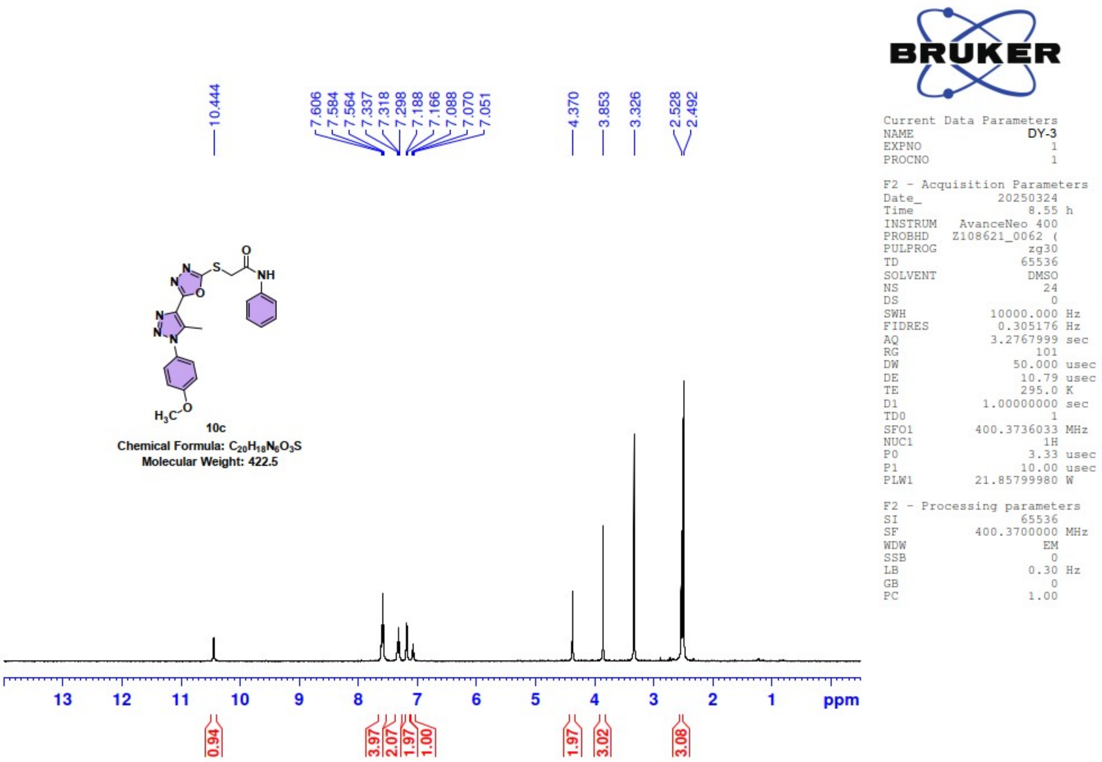

Fig. S4: ^{1}H NMR spectra of final compound 10d

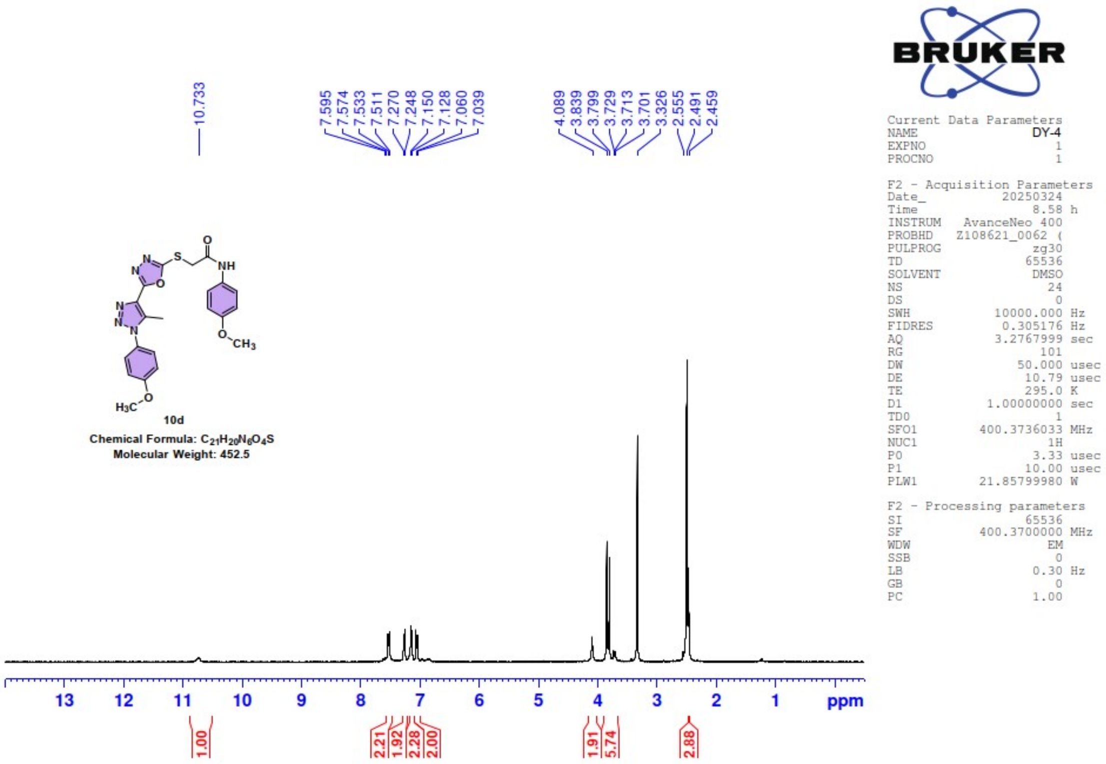

Fig. S5: ^{1}H NMR spectra of final compound 10e

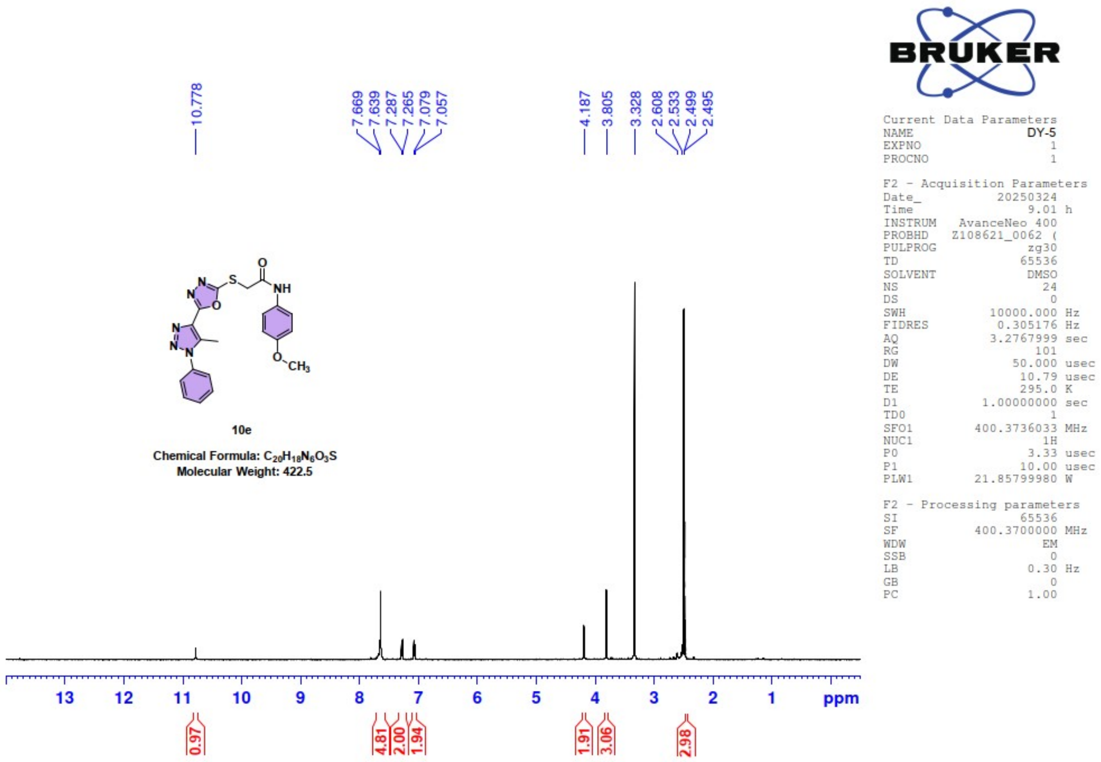

Fig. S6: ^{1}H NMR spectra of final compound 10f

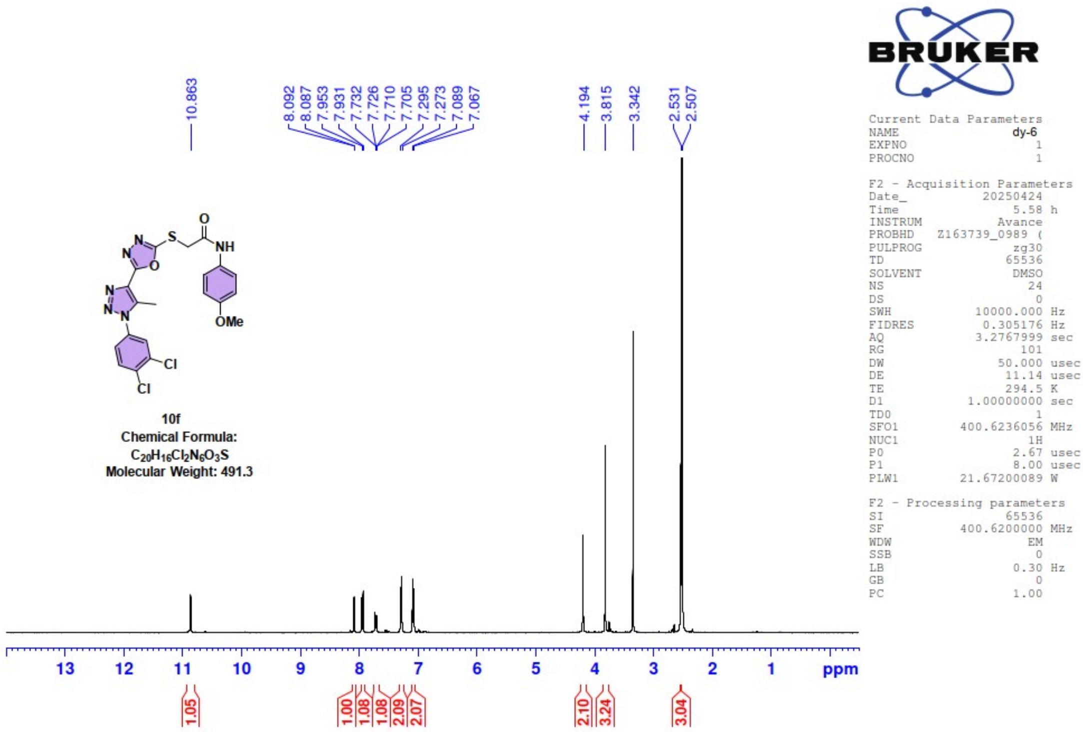

Fig. S7: ^{1}H NMR spectra of final compound 10g

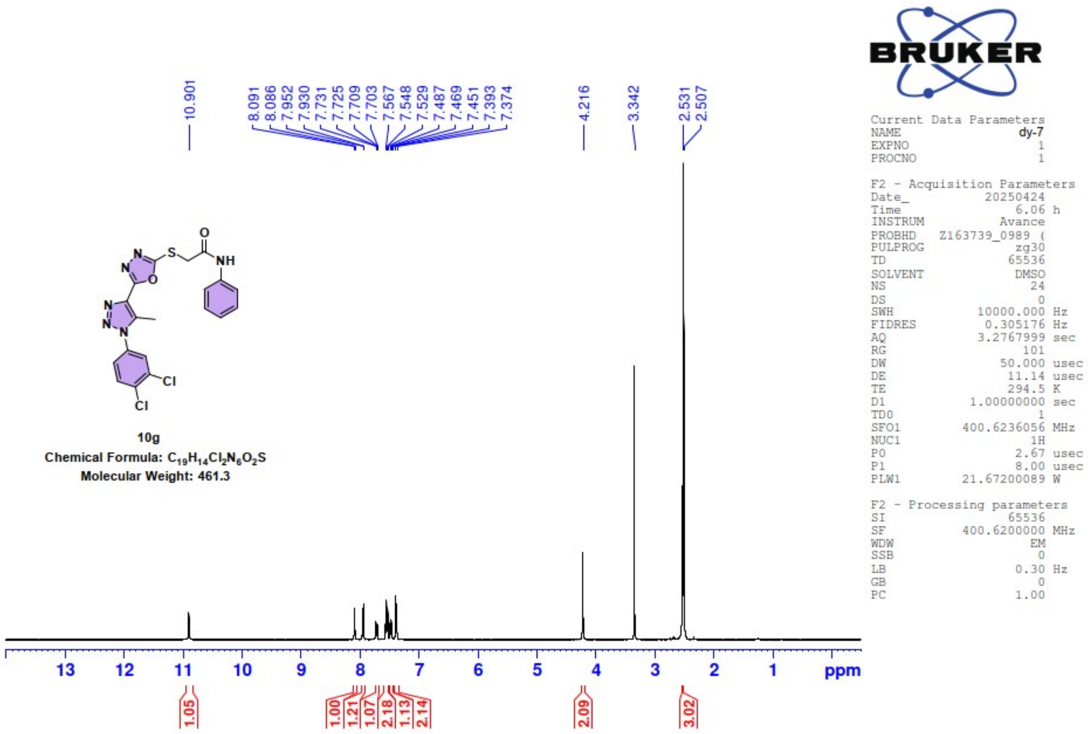

Fig. S8: ^{1}H NMR spectra of final compound 10h

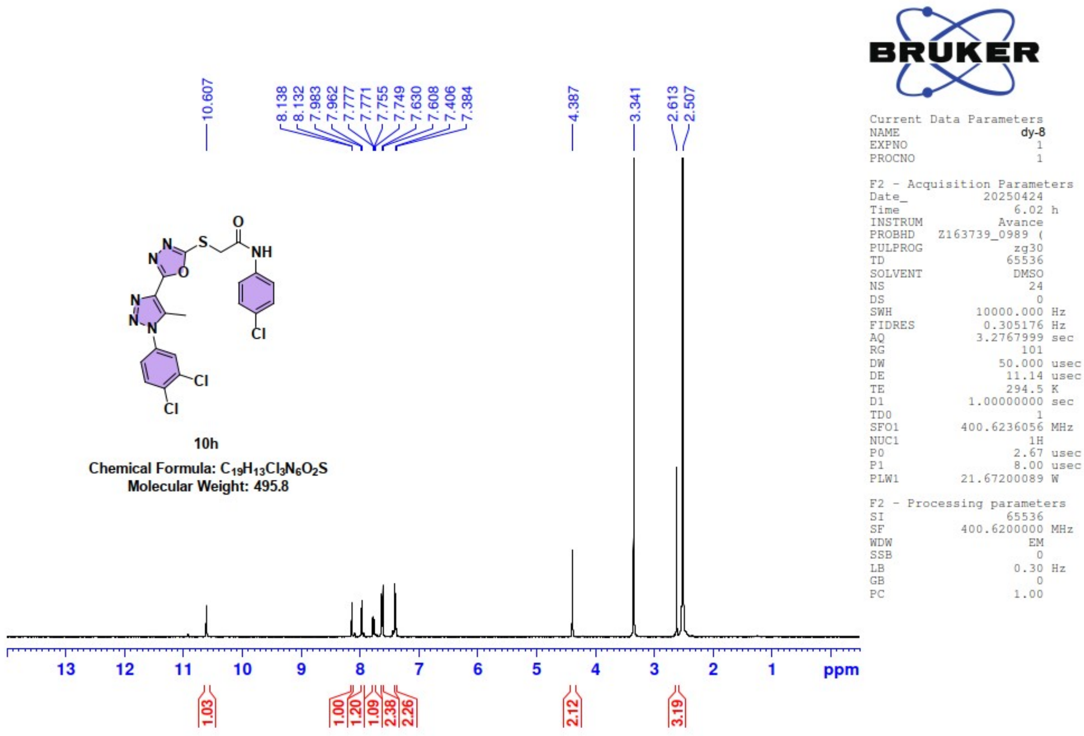

Fig. S9: ^{1}H NMR spectra of final compound 10i

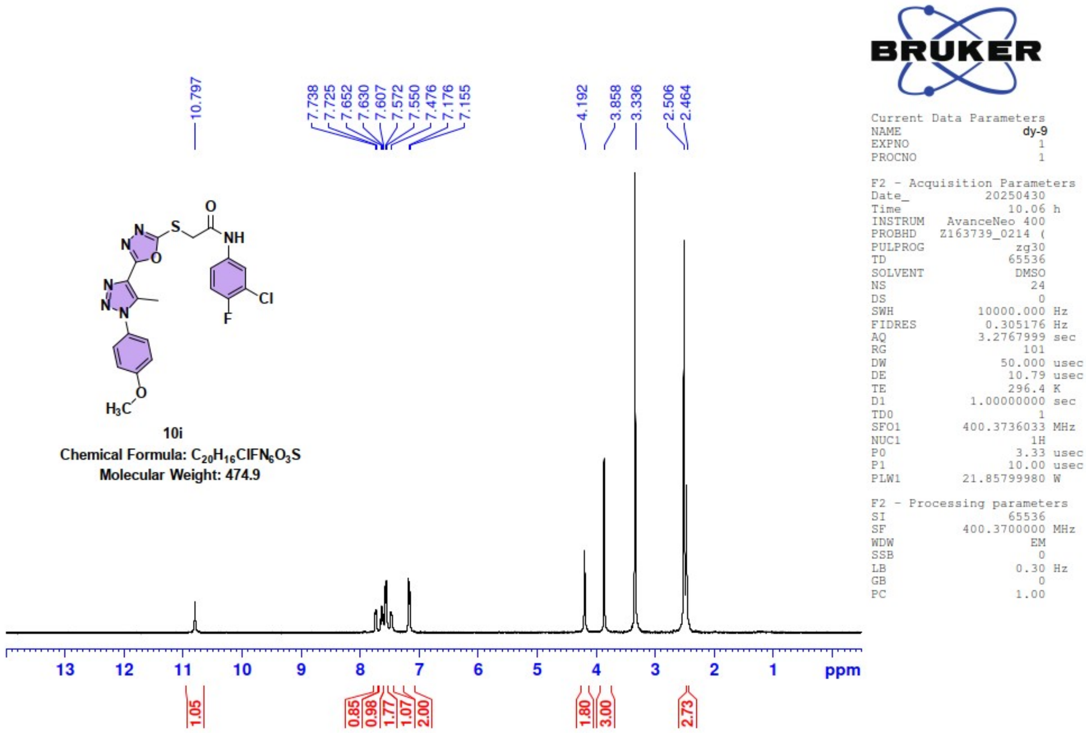

Fig. S10: ^{1}H NMR spectra of final compound 11a

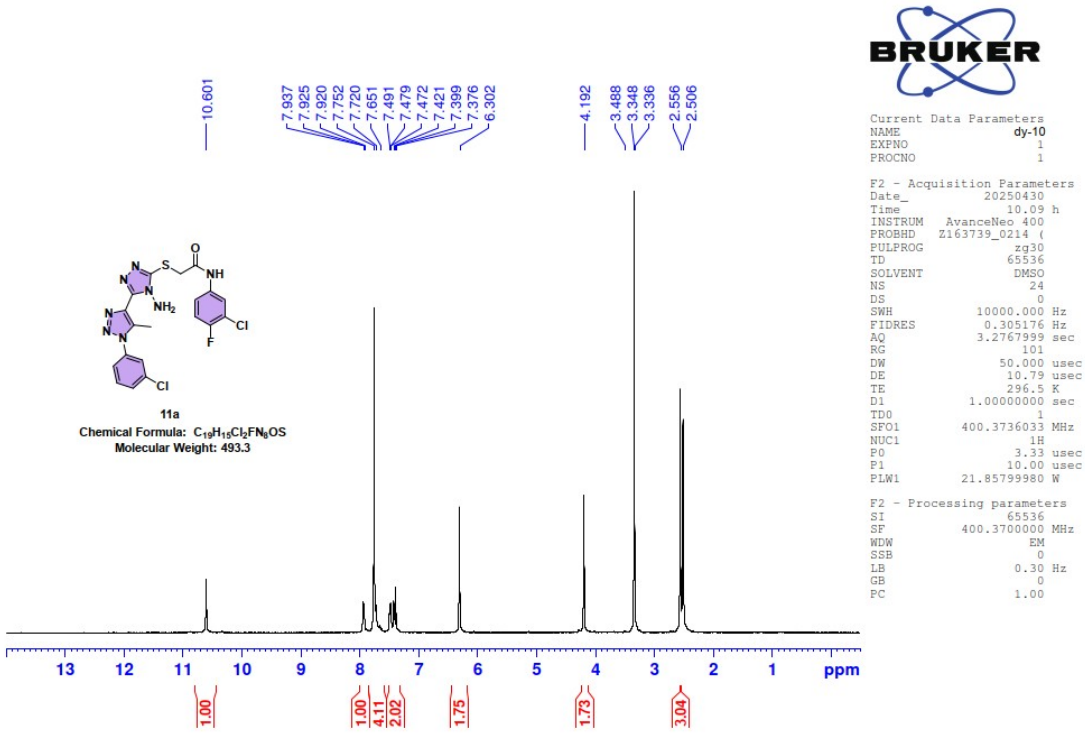

Fig. S11: ^{1}H NMR spectra of final compound 11b

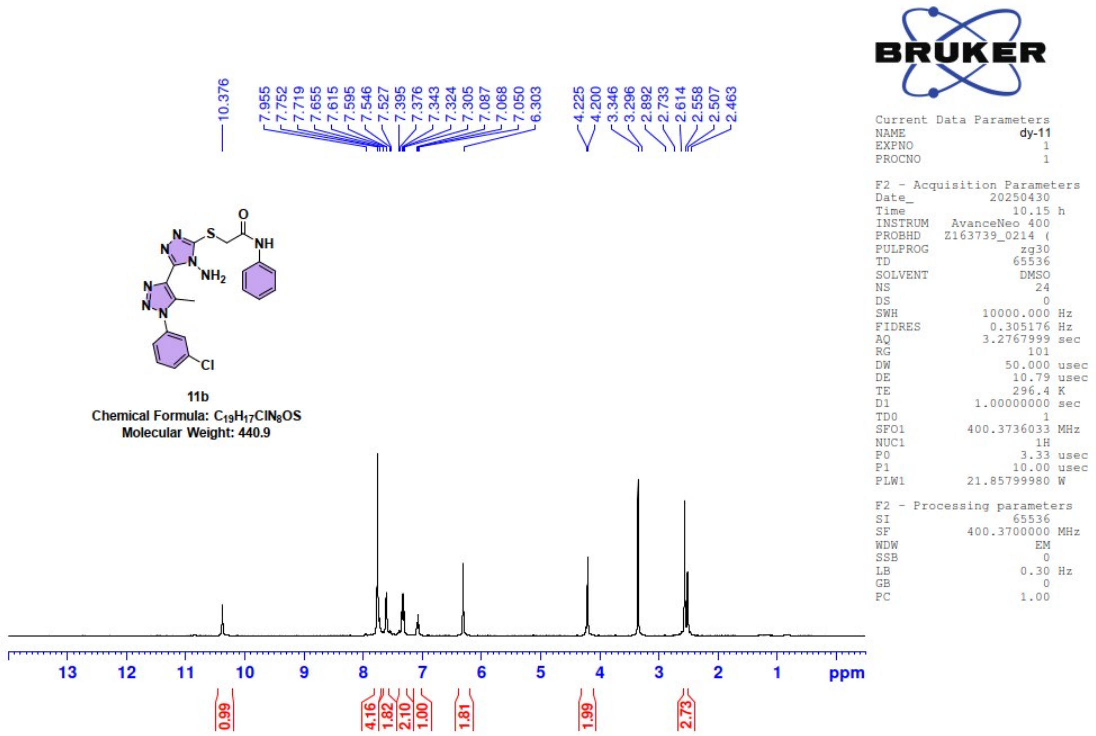

Fig. S12: ^{1}H NMR spectra of final compound 11c

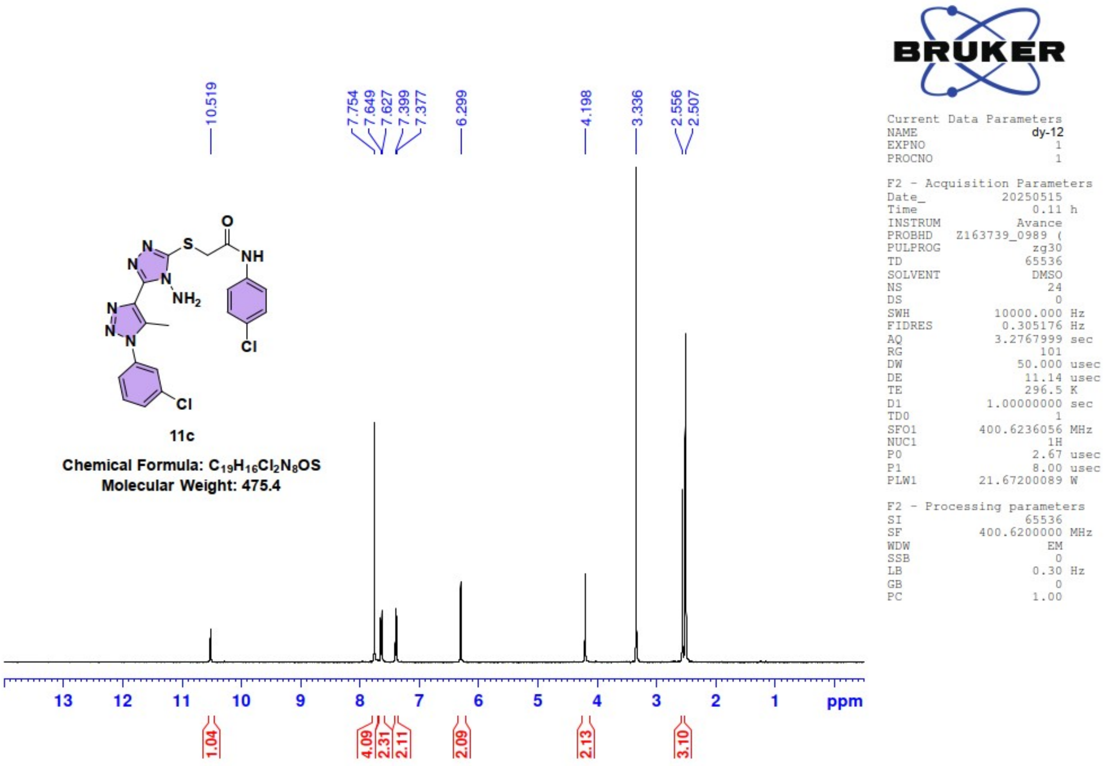

Fig. S13: ^{1}H NMR spectra of final compound 11d

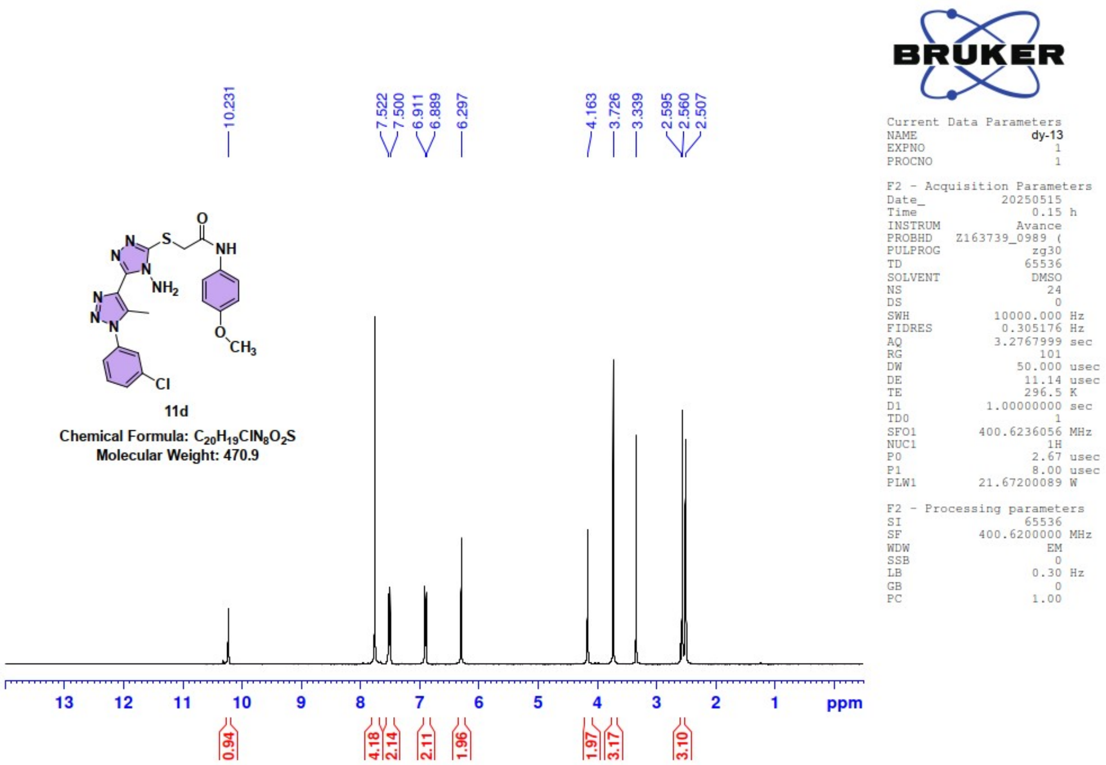

Fig. S14: Mass spectra of final compound 10a

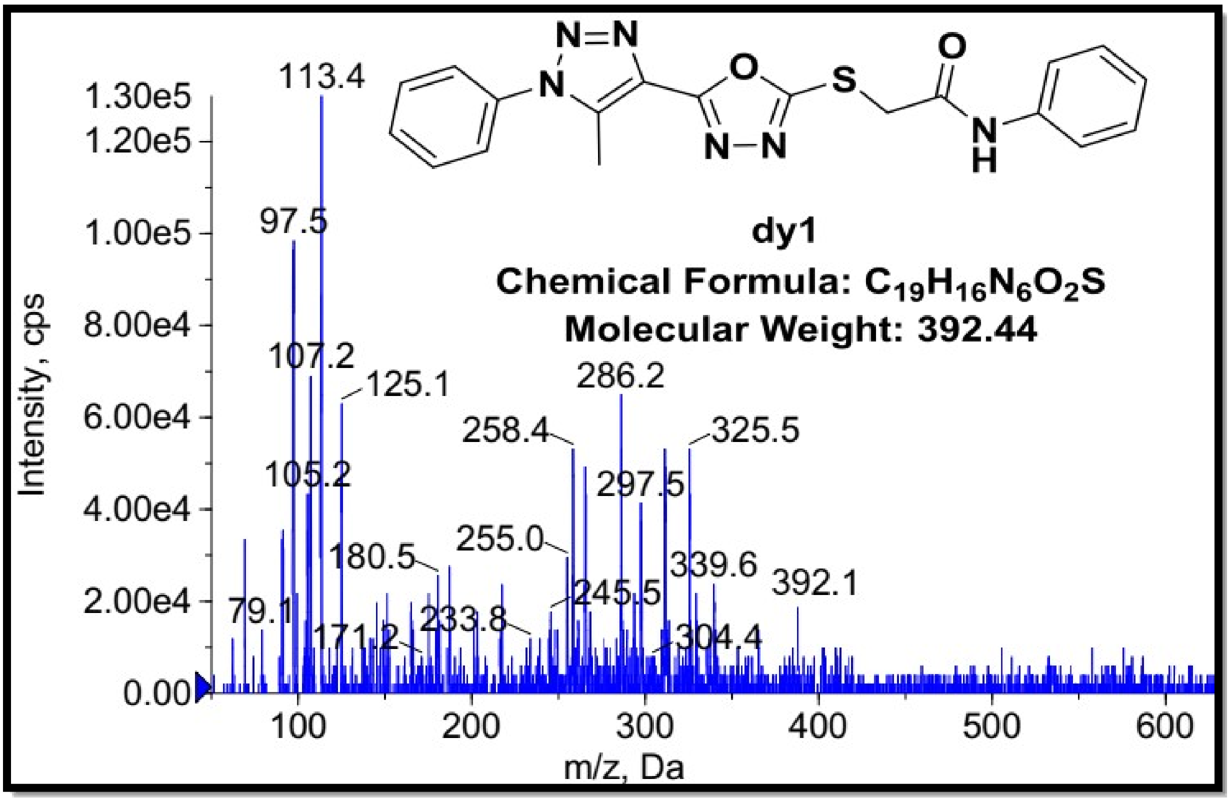

Fig. S15: Mass spectra of final compound 10b

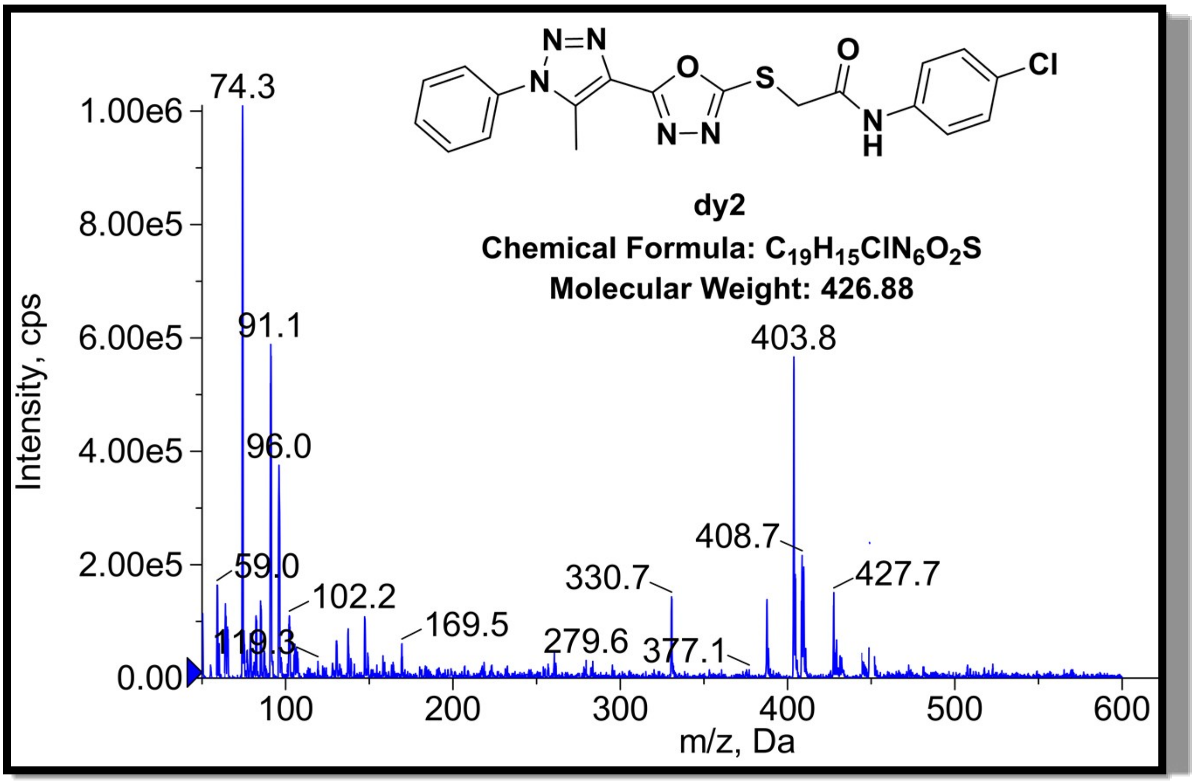

Fig. S16: Mass spectra of final compound 10c

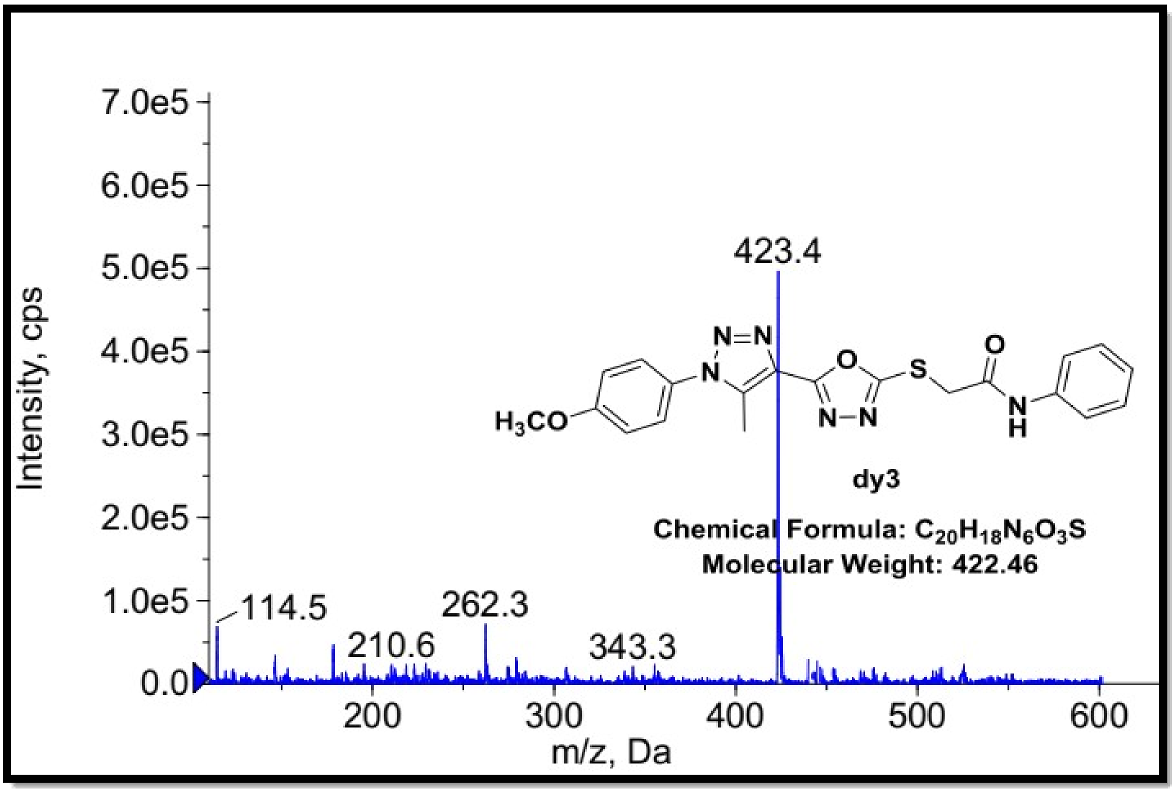

Fig. S17: Mass spectra of final compound 10d

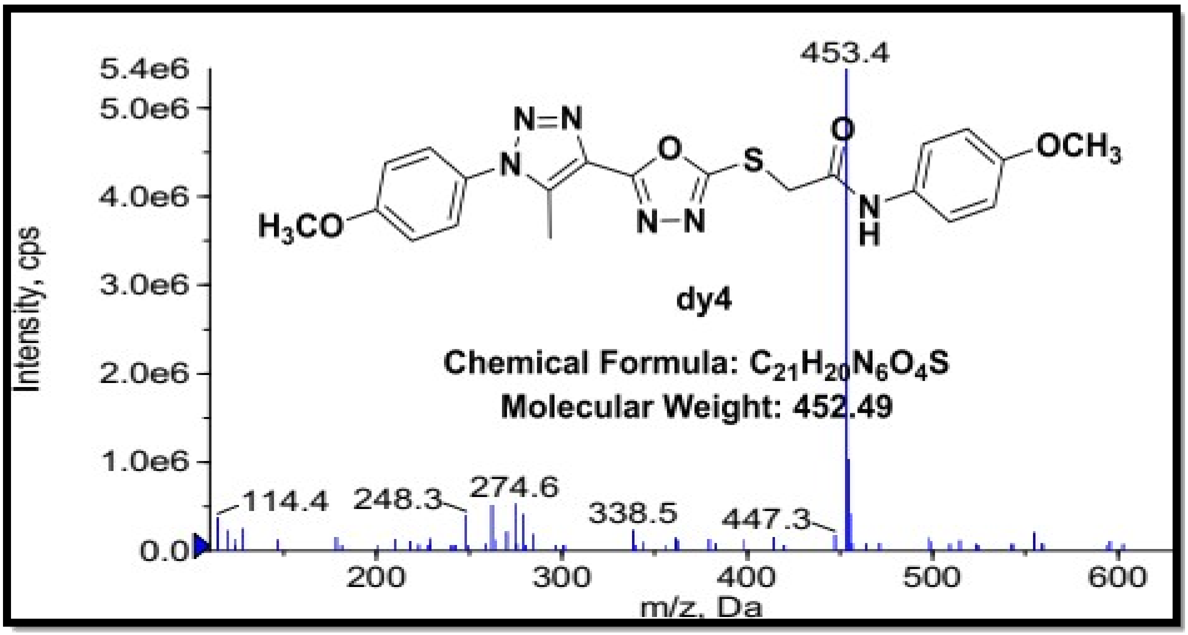

Fig. S18: Mass spectra of final compound 10e

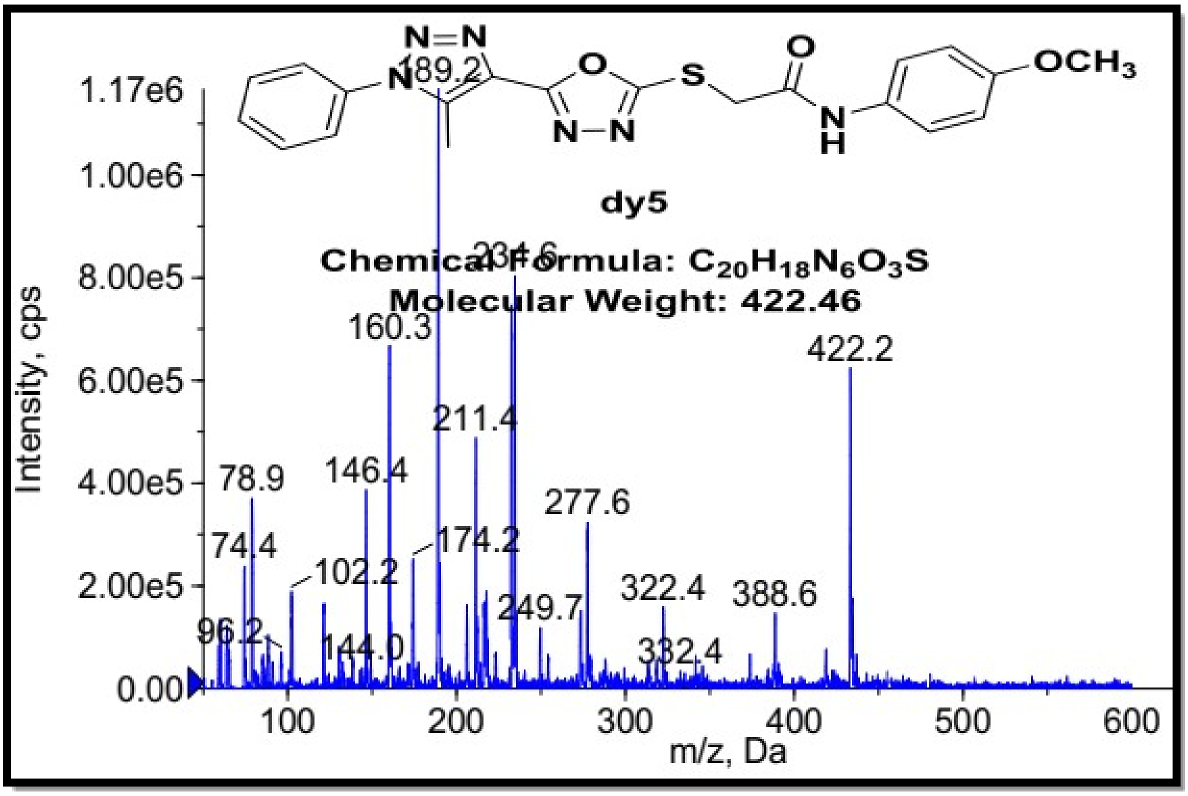

Fig. S19: Mass spectra of final compound 10f

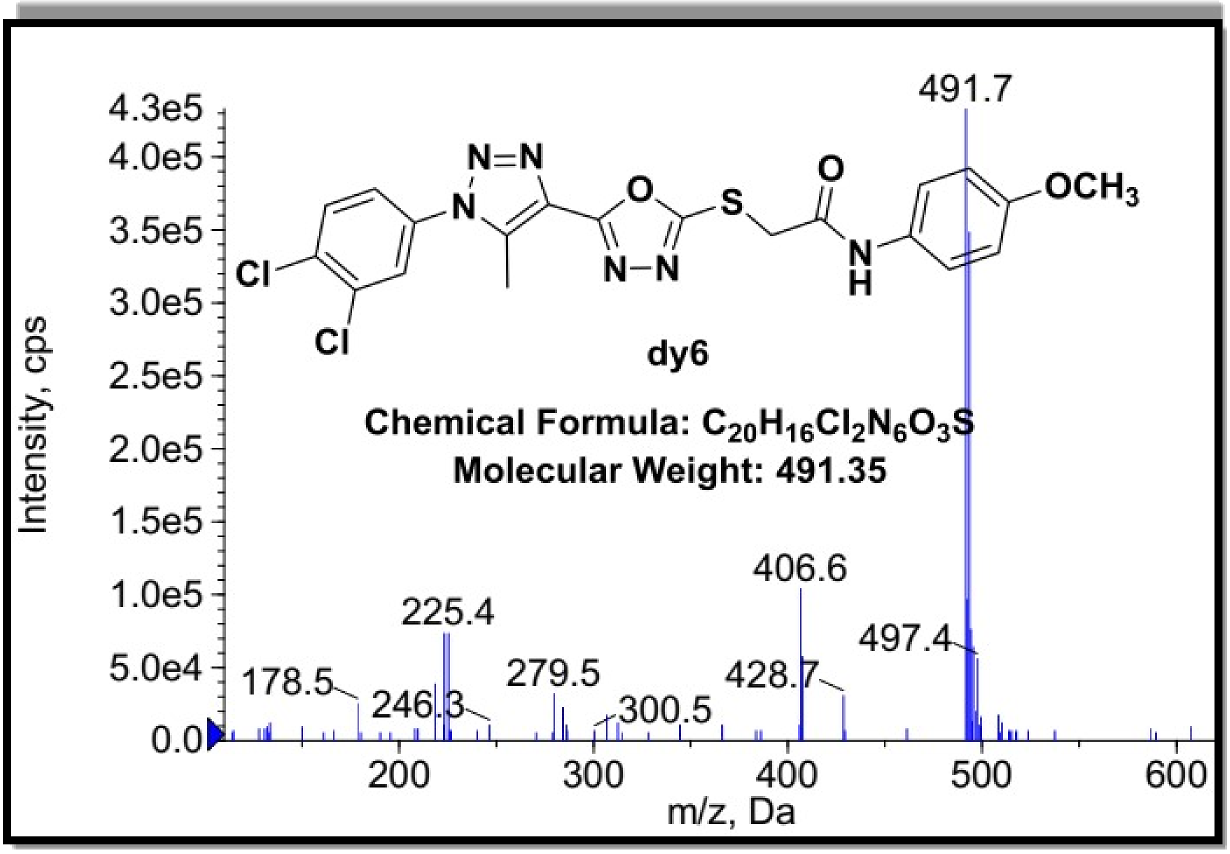

Fig. S20: Mass spectra of final compound 10g

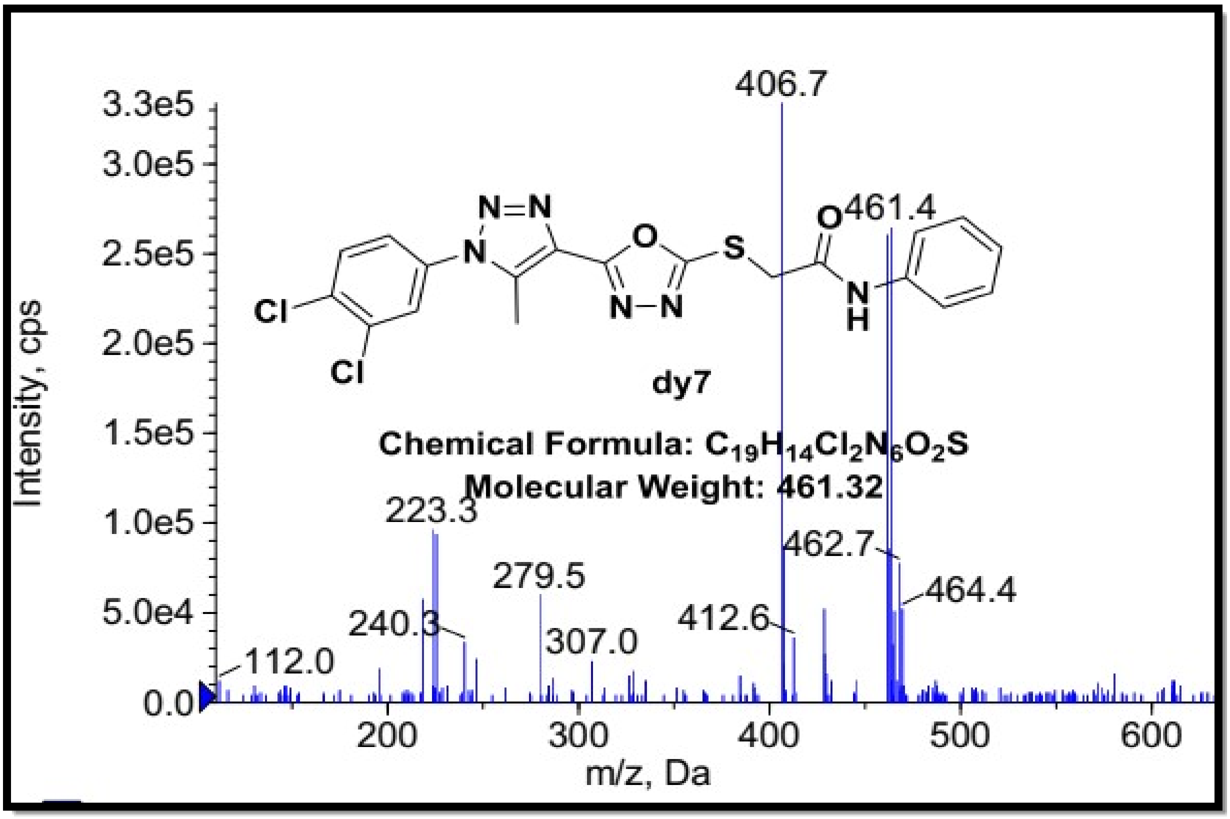

Fig. S21: Mass spectra of final compound 10h

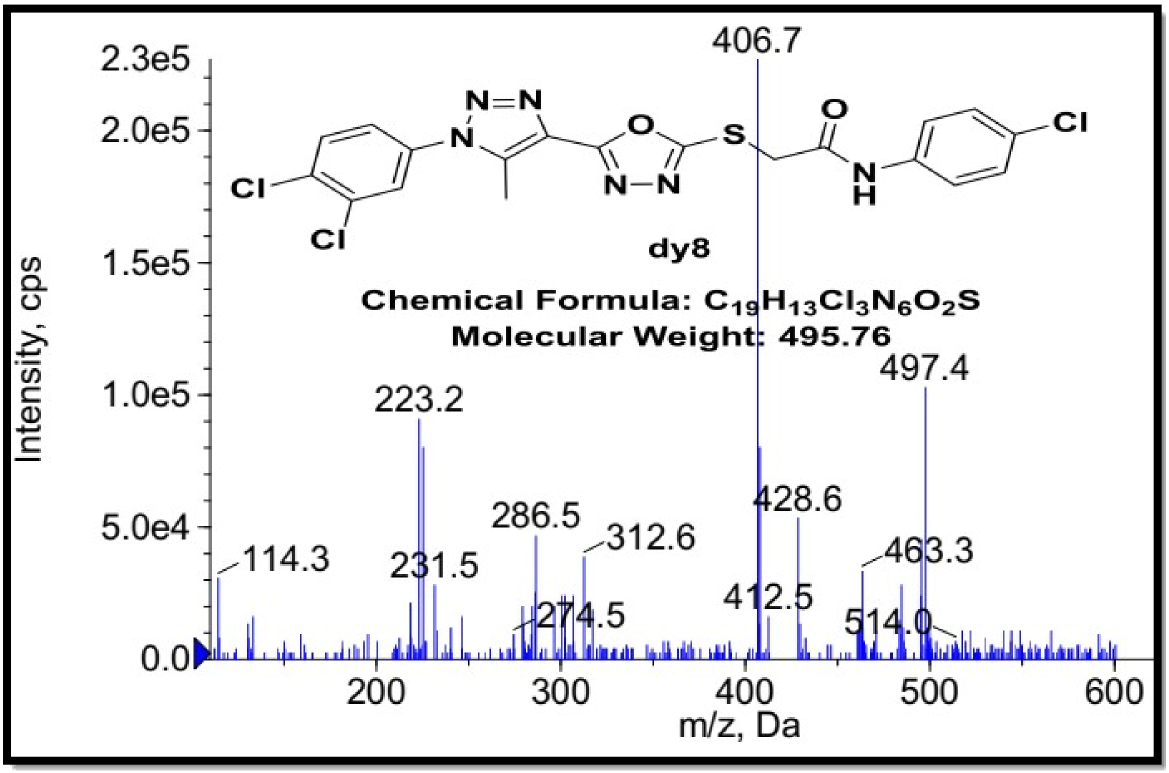

Fig. S22: Mass spectra of final compound 10i

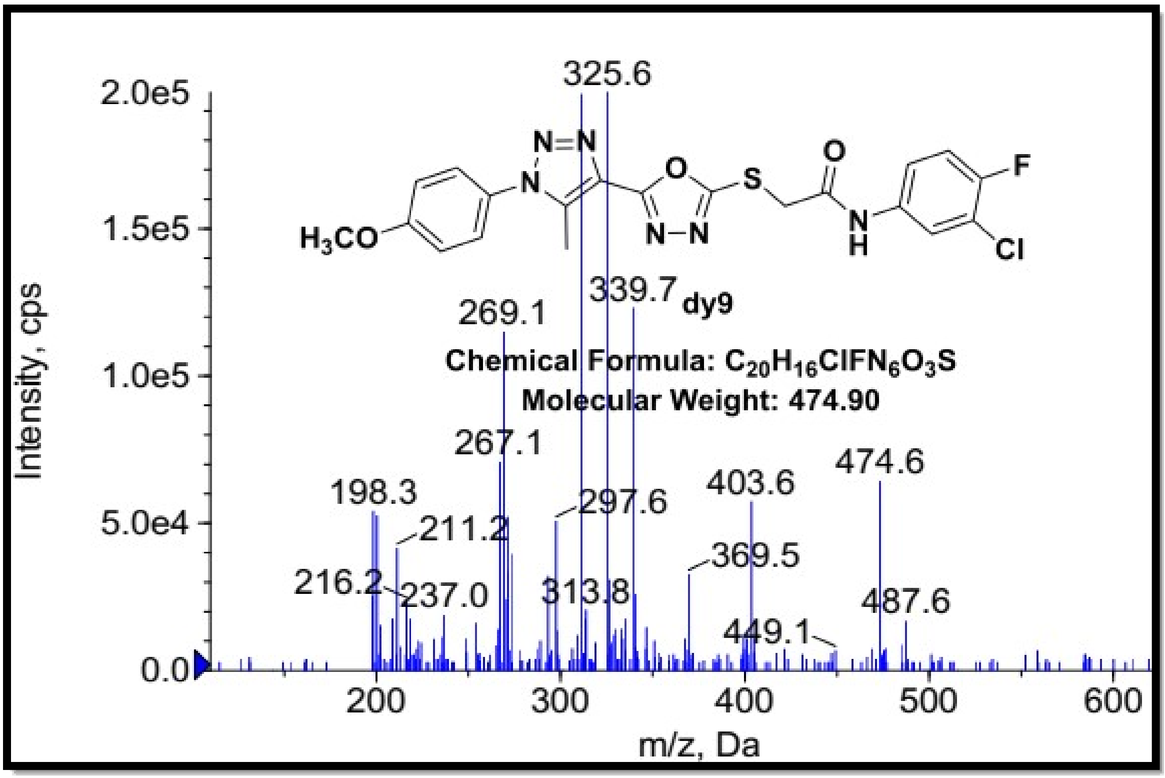

Fig. S23: Mass spectra of final compound 11a

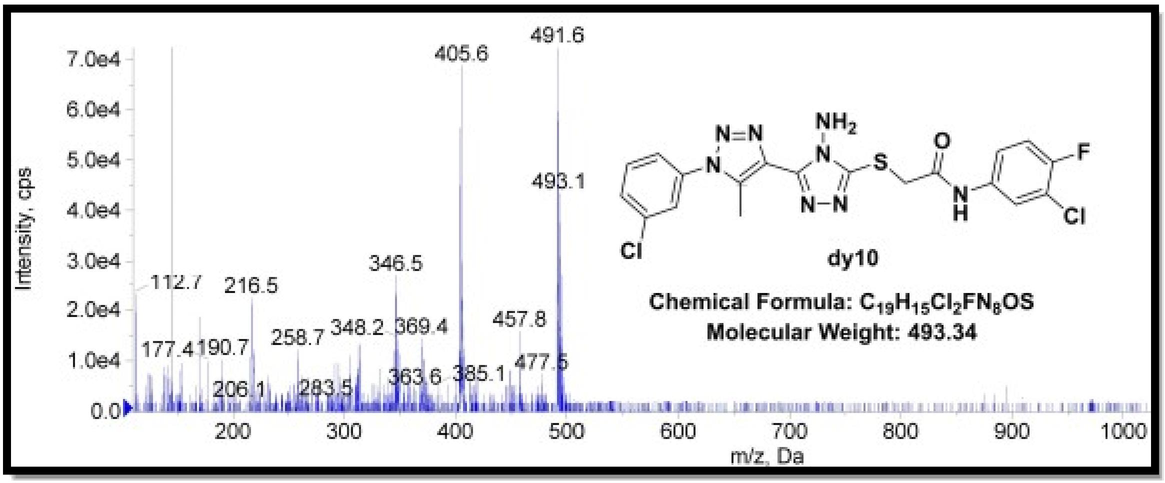

Fig. S24: Mass spectra of final compound 11b

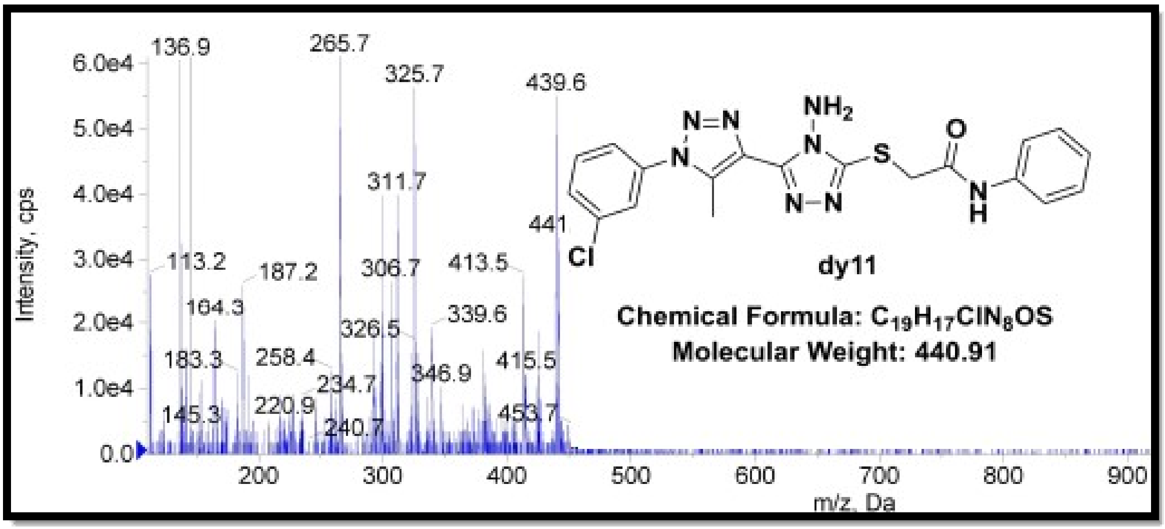

Fig. S25: Mass spectra of final compound 11c

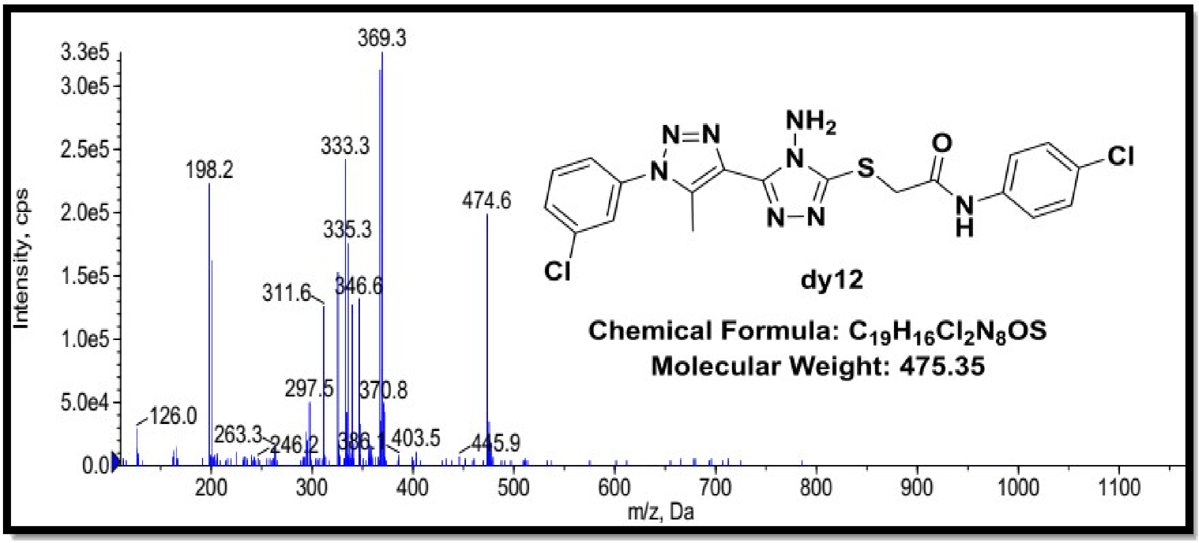

Fig. S26: Mass spectra of final compound 11d

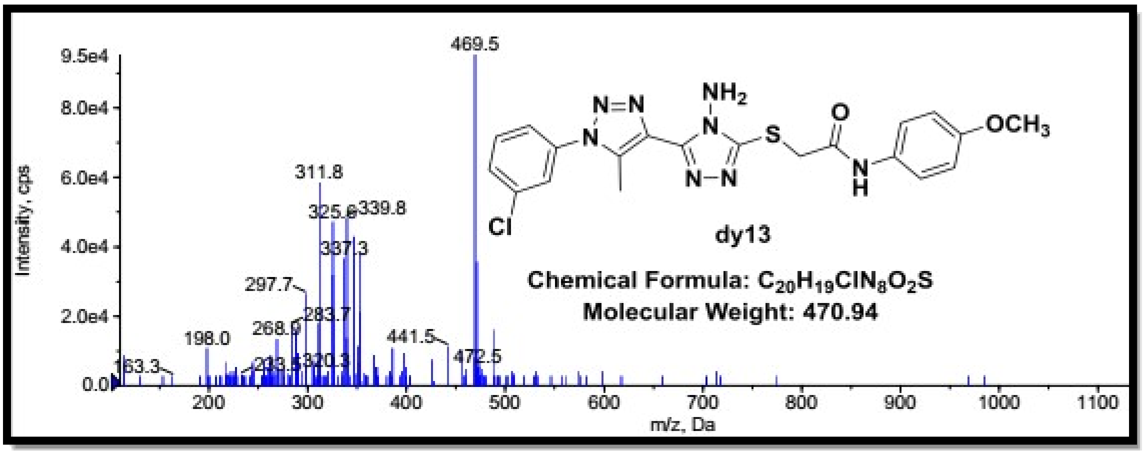

Fig. S27: IR spectra of final compound 10a

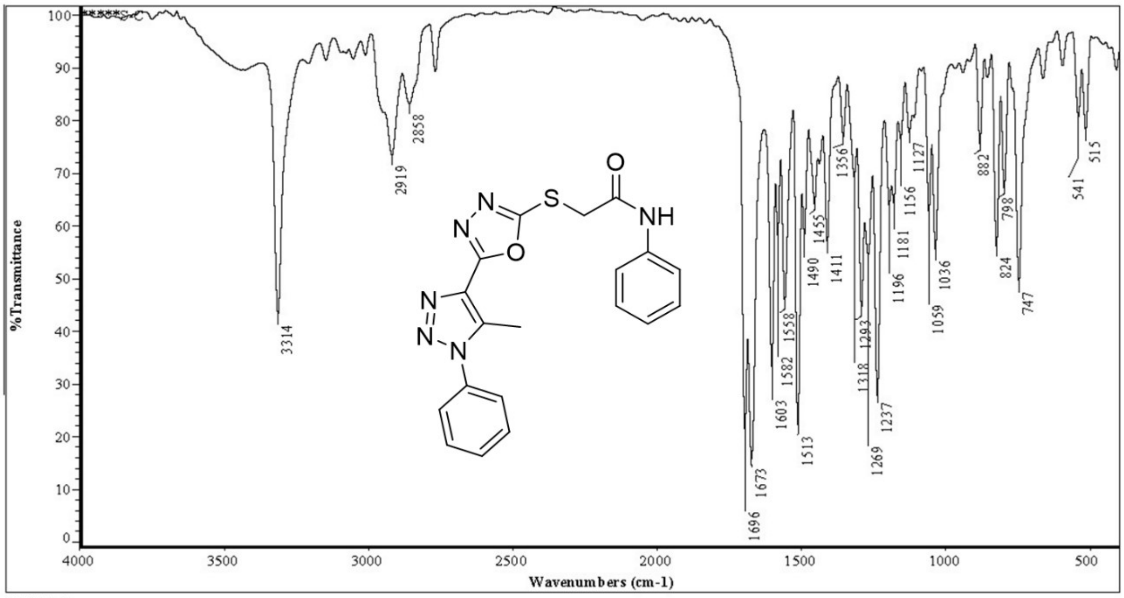

*****S-C

Number of sample scans: 32
Number of background scans: 32
Resolution: 4.000
Sample gain: 2.0
Optical velocity: 0.4747
Aperture: 80.00

ThermoFisher SCIENTIFIC

Mansoura University
Faculty of Science
Spectral Analyses unit
Chemistry Department
ThermoFisher Nicolete IS10, USA
Spectral range: 4000 - 400 cm-1

Fig. S28: IR spectra of final compound 10b

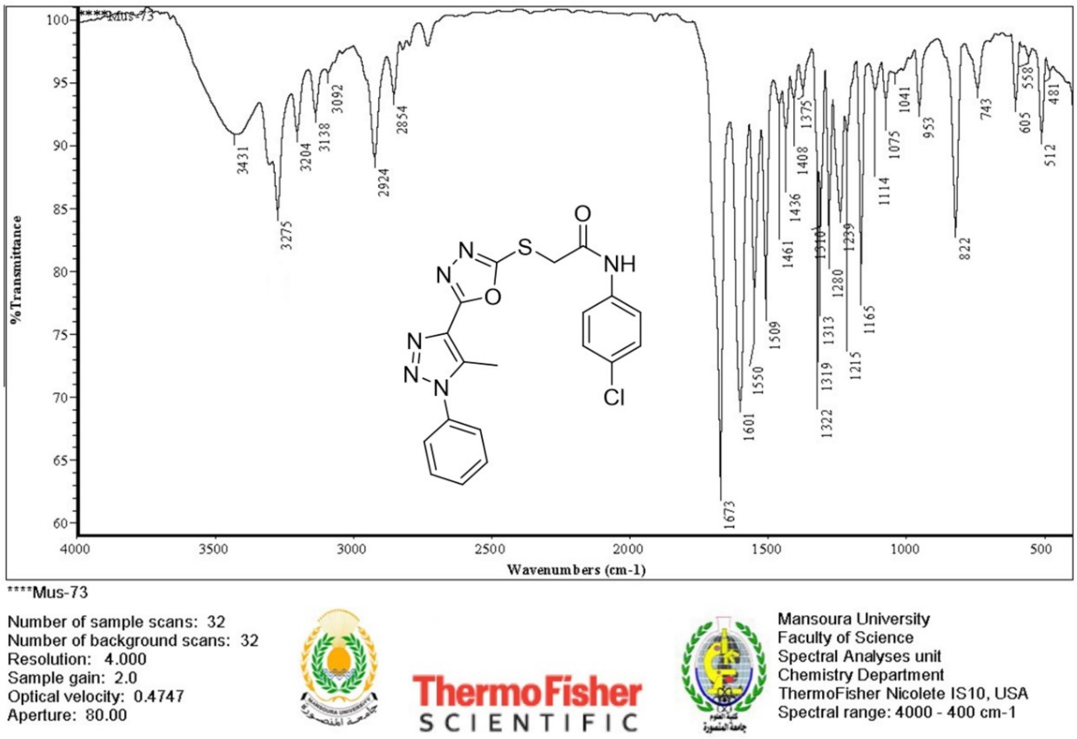

Fig. S29: IR spectra of final compound 10c

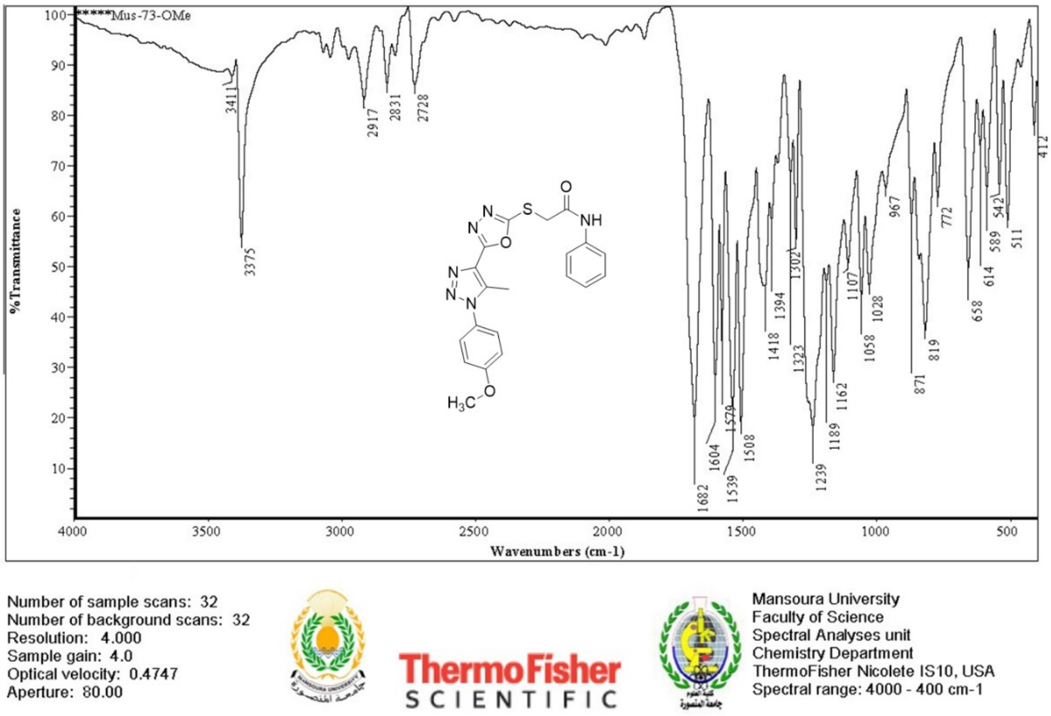

Fig. S30: IR spectra of final compound 10d

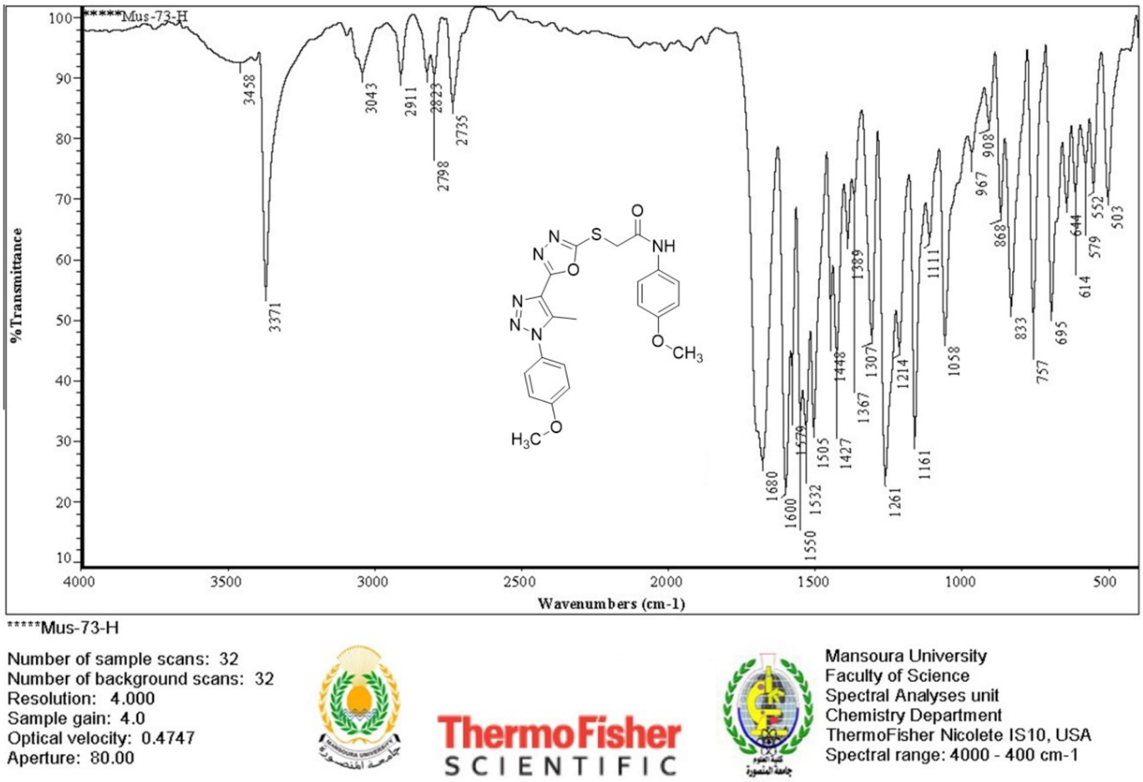

Fig. S31: IR spectra of final compound 10e

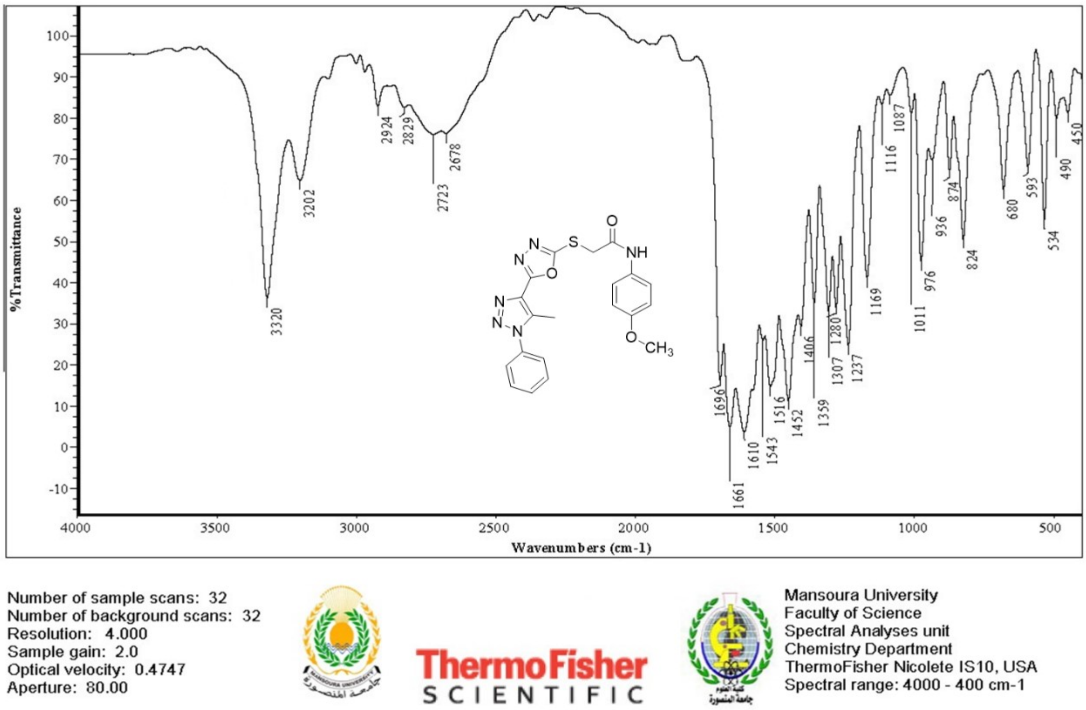

Fig. S32: IR spectra of final compound 10f

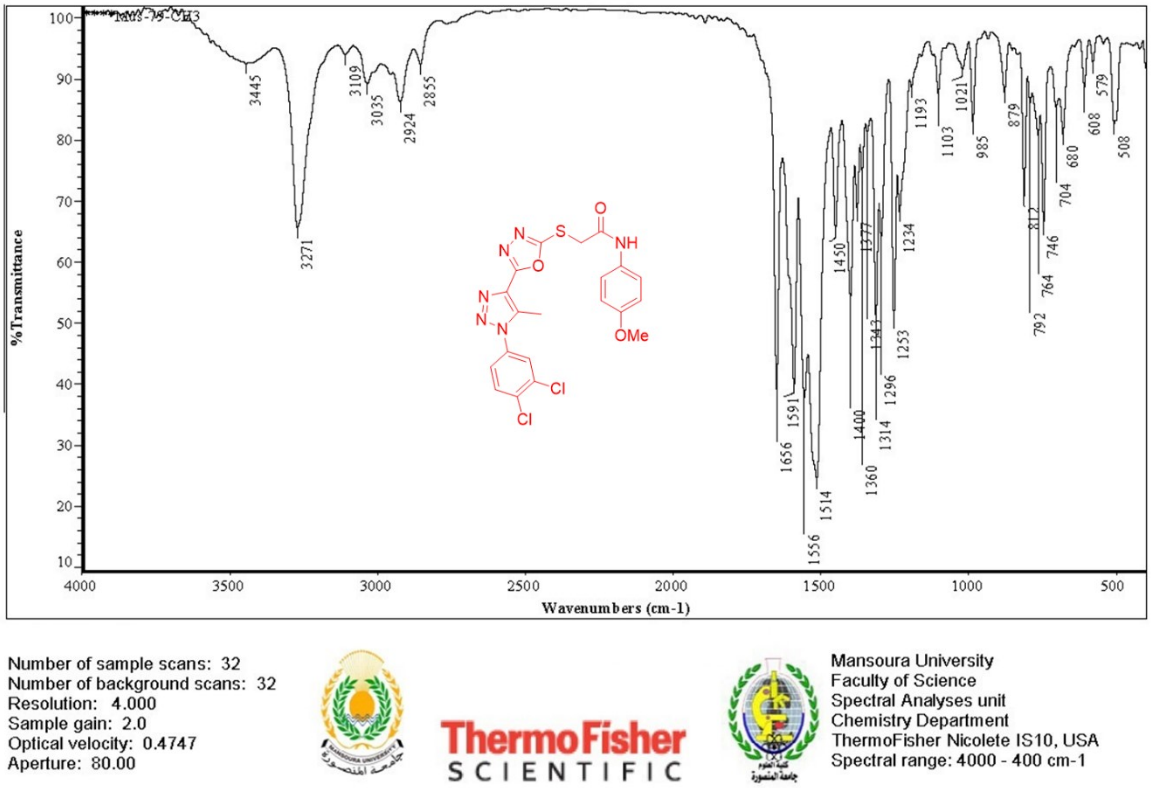

Fig. S33: IR spectra of final compound 10g

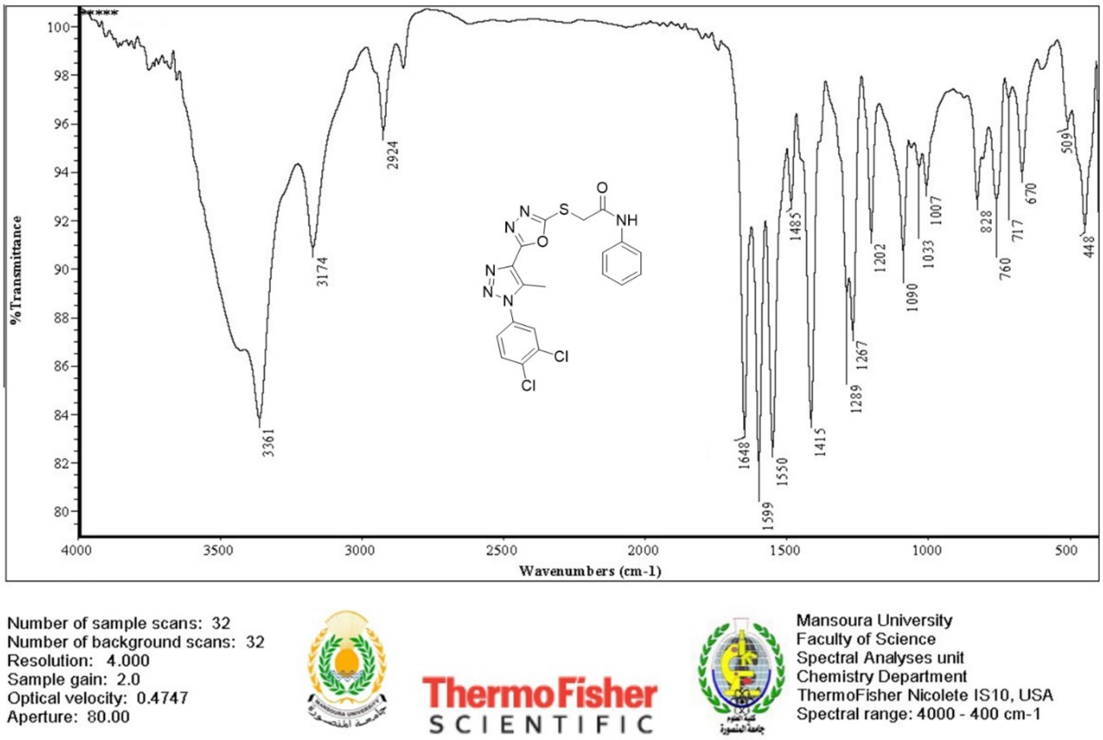

Fig. S34: IR spectra of final compound 10h

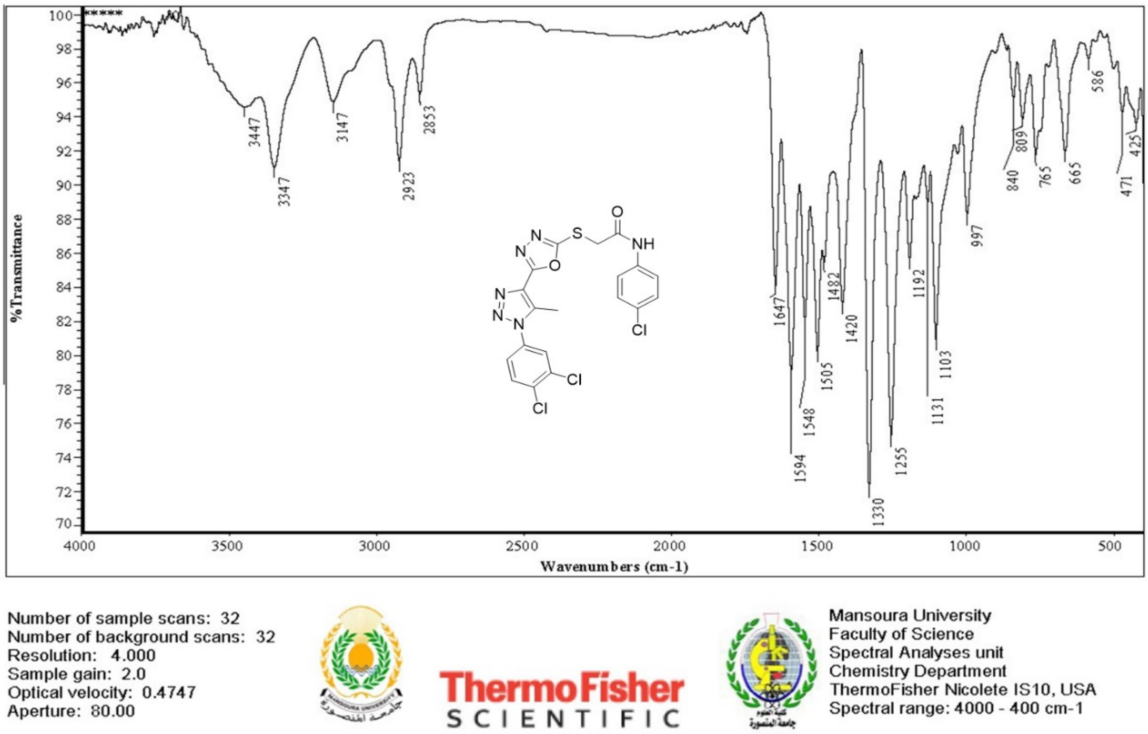

Fig. S35: IR spectra of final compound 10i

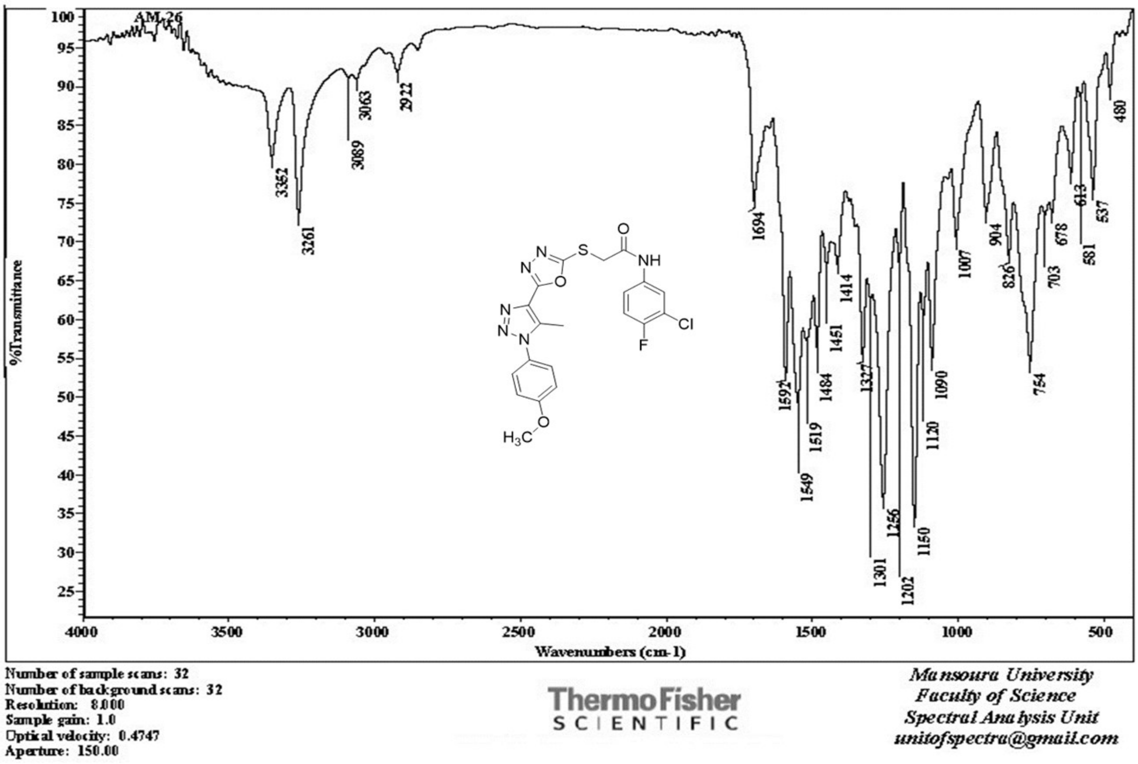

Fig. S36: IR spectra of final compound 11a

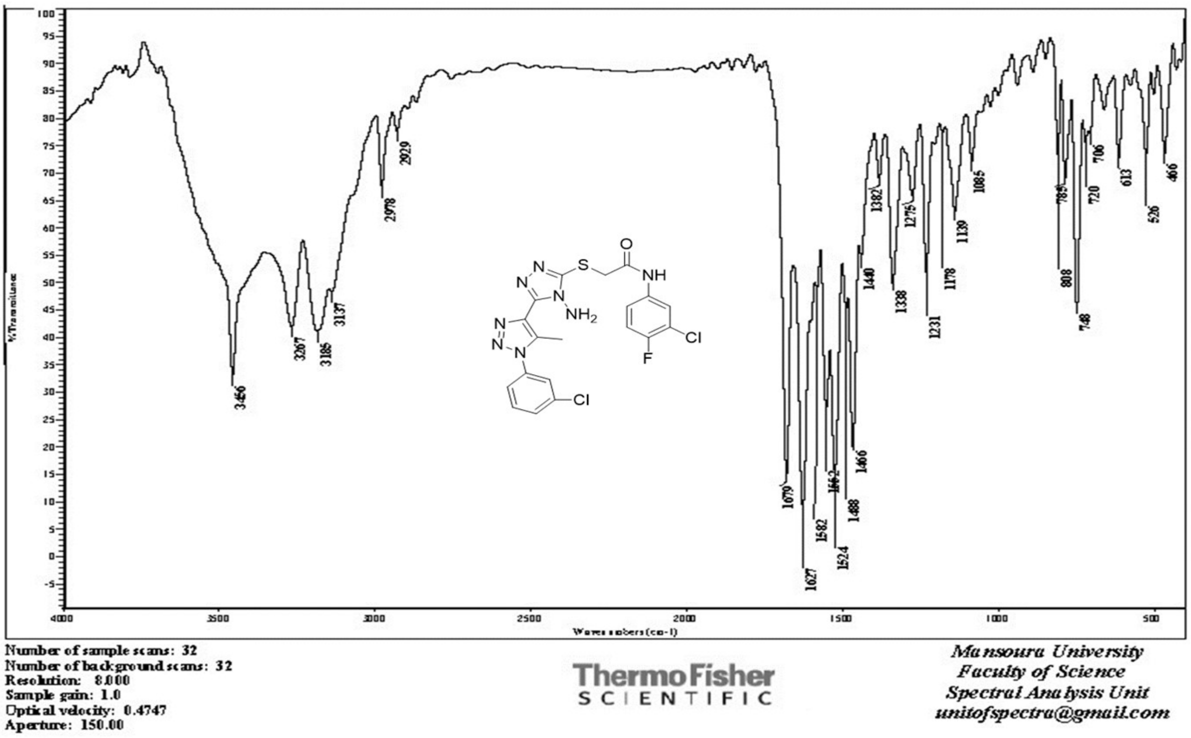

Fig. S37: IR spectra of final compound 11b

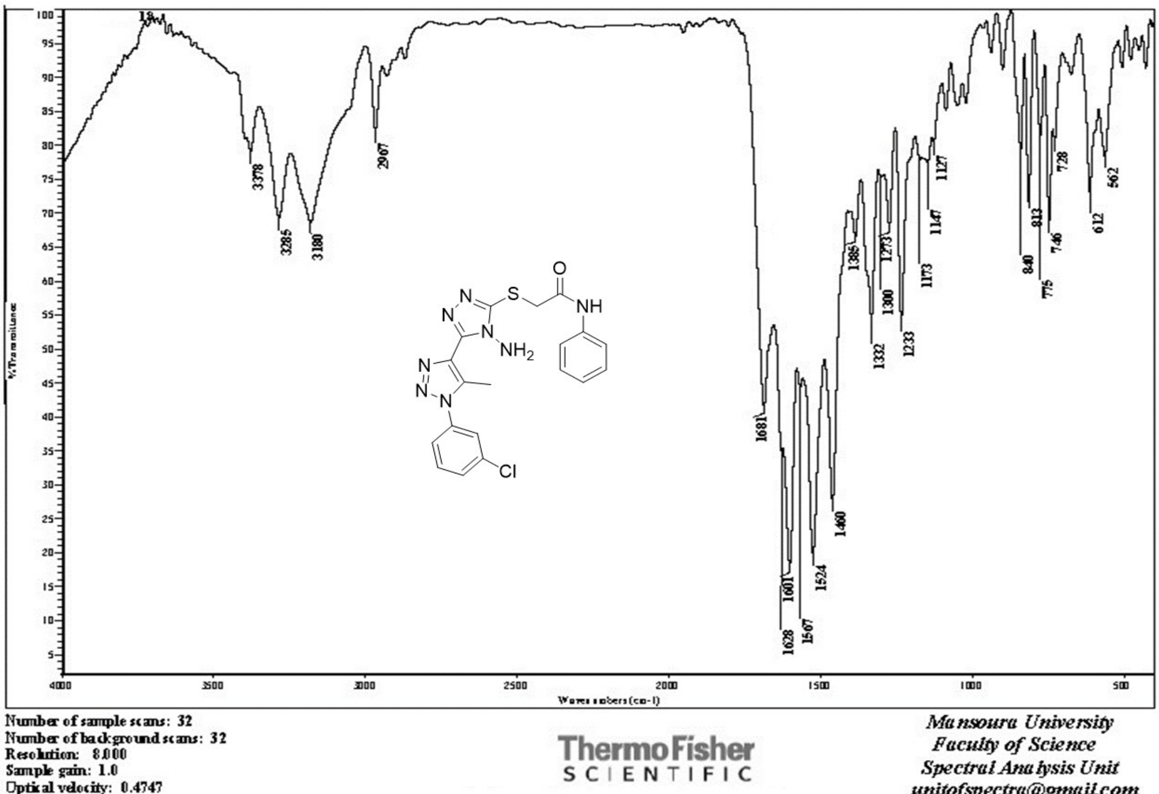

Fig. S38: IR spectra of final compound 11c

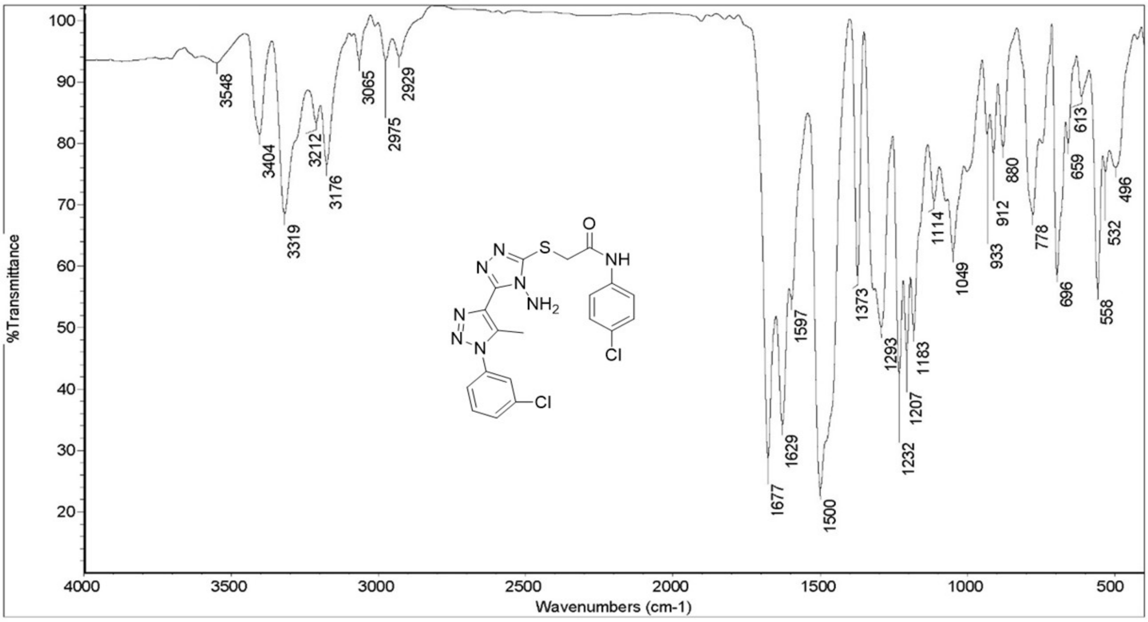

Number of sample scans: 32
Number of background scans: 32
Resolution: 4.000
Sample gain: 1.0
Optical velocity: 0.6329
Aperture: 80.00

ThermoFisher SCIENTIFIC

Mansoura University
Faculty of Science
Spectral Analyses Unit
Chemistry department
ThermoFisher Nicolete IS10, USA
spectral range : 4000-400 cm-1

Fig. S39: IR spectra of final compound 11d

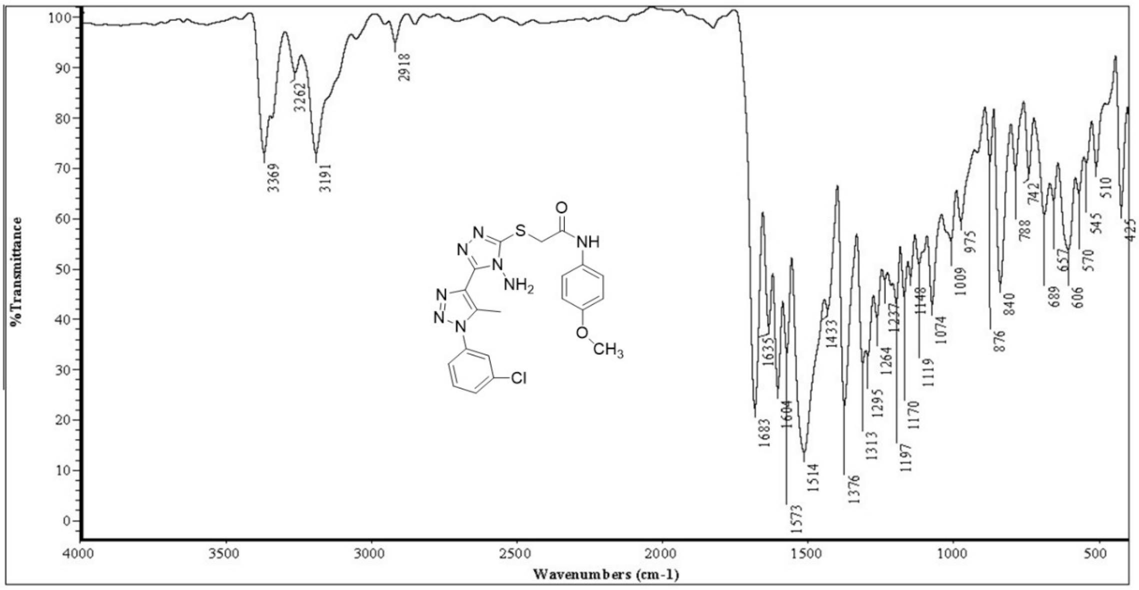

Number of sample scans: 32
Number of background scans: 32
Resolution: 4.000
Sample gain: 2.0
Optical velocity: 0.4747
Aperture: 80.00

ThermoFisher SCIENTIFIC

Mansoura University
Faculty of Science
Spectral Analyses unit
Chemistry Department
ThermoFisher Nicolete IS10, USA
Spectral range: 4000 - 400 cm-1

Fig. S40: ^{13}C NMR spectra of final compound 10a

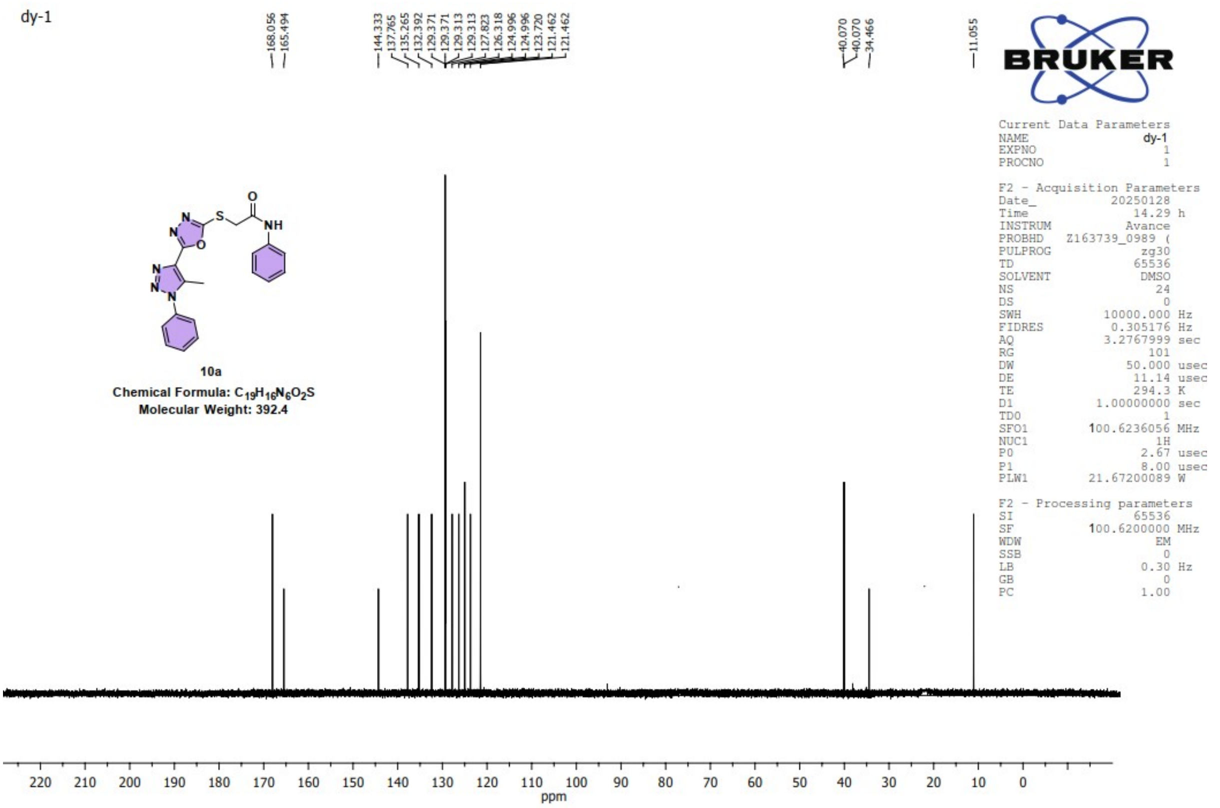

Fig. S41: ^{13}C NMR spectra of final compound 10b

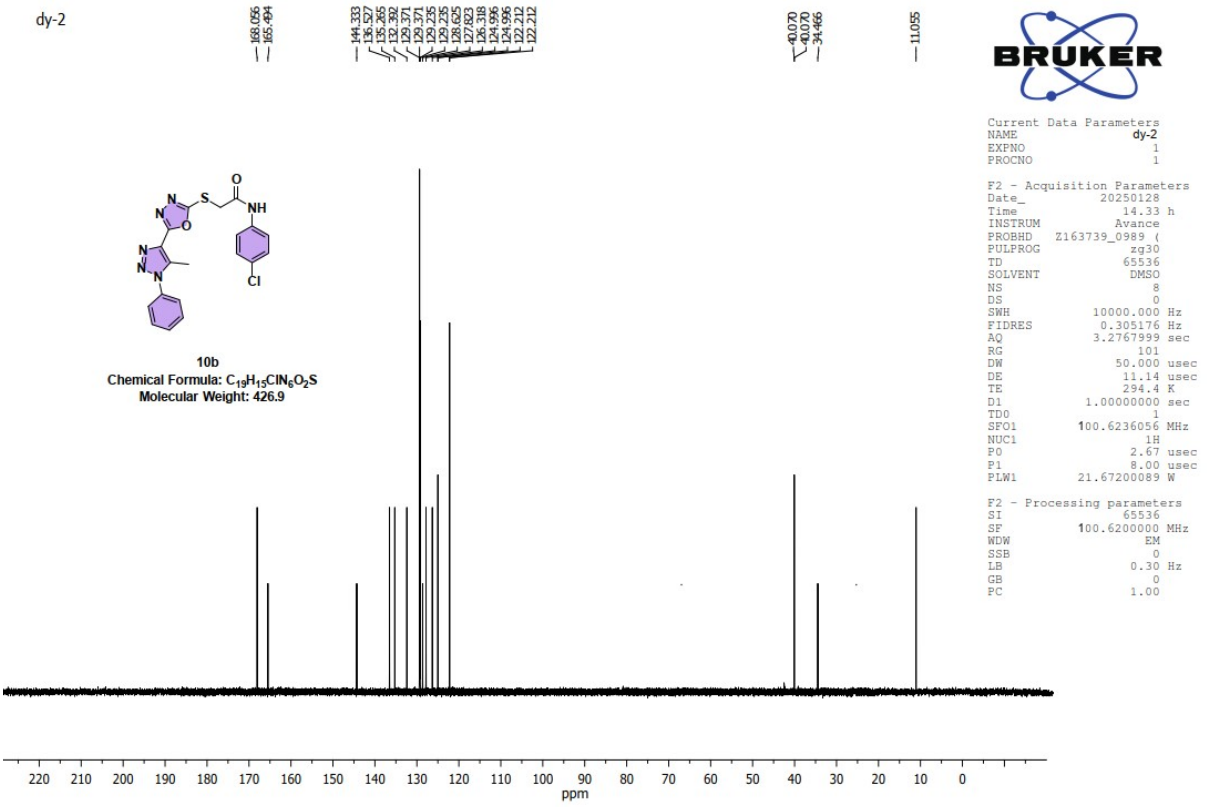

Fig. S42: ^{13}C NMR spectra of final compound 10c

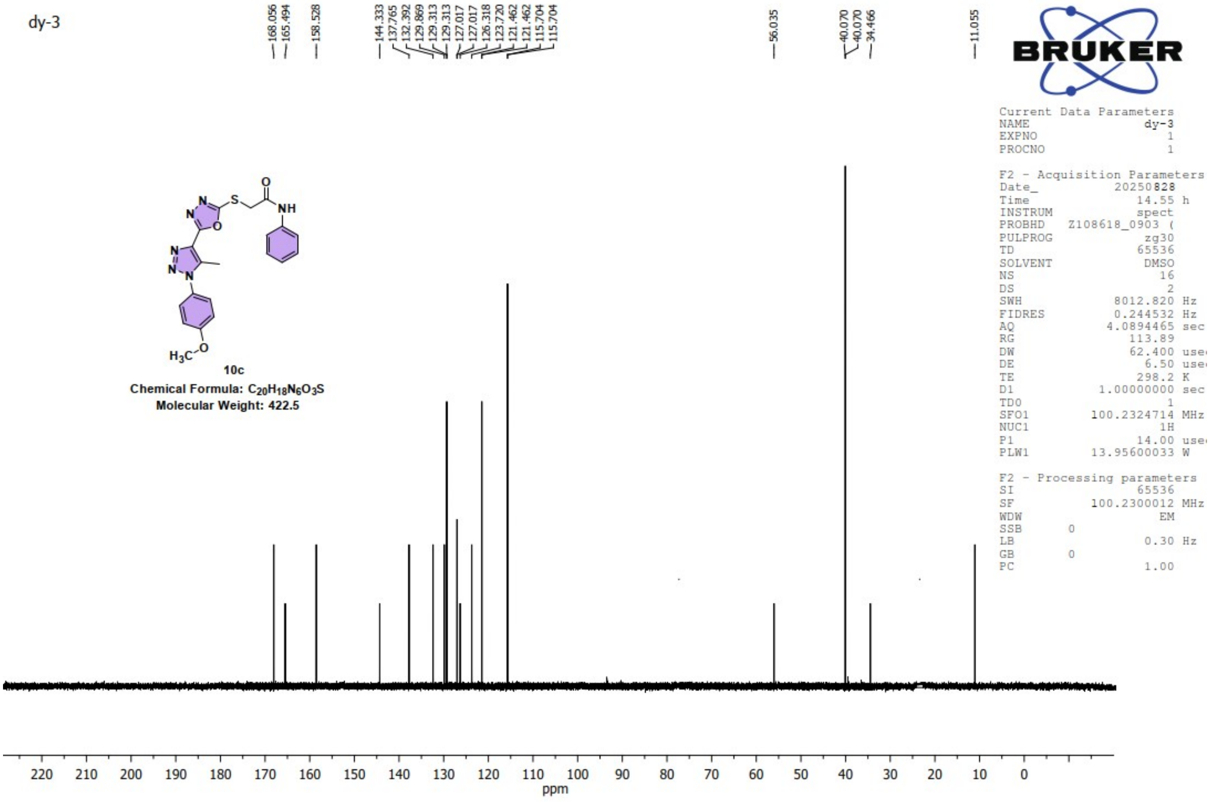

Fig. S43: ^{13}C NMR spectra of final compound 10d

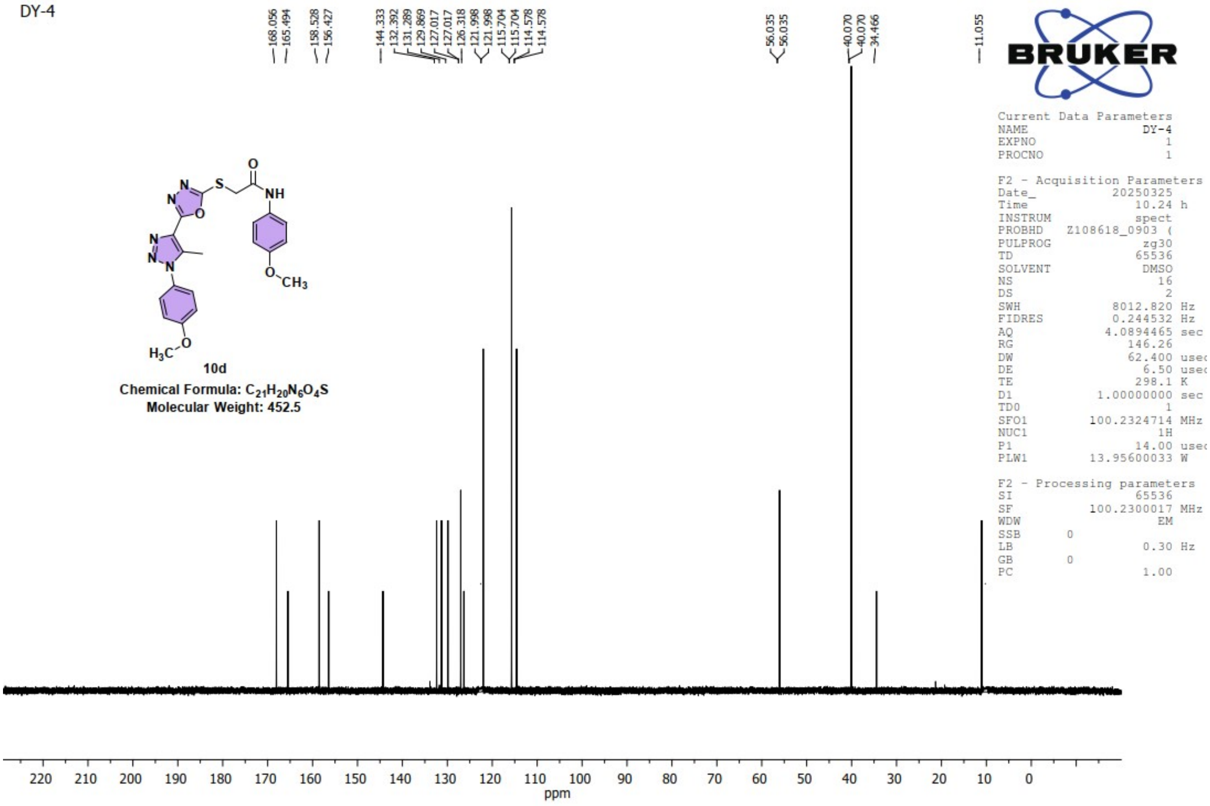

Fig. S44: ^{13}C NMR spectra of final compound 10e

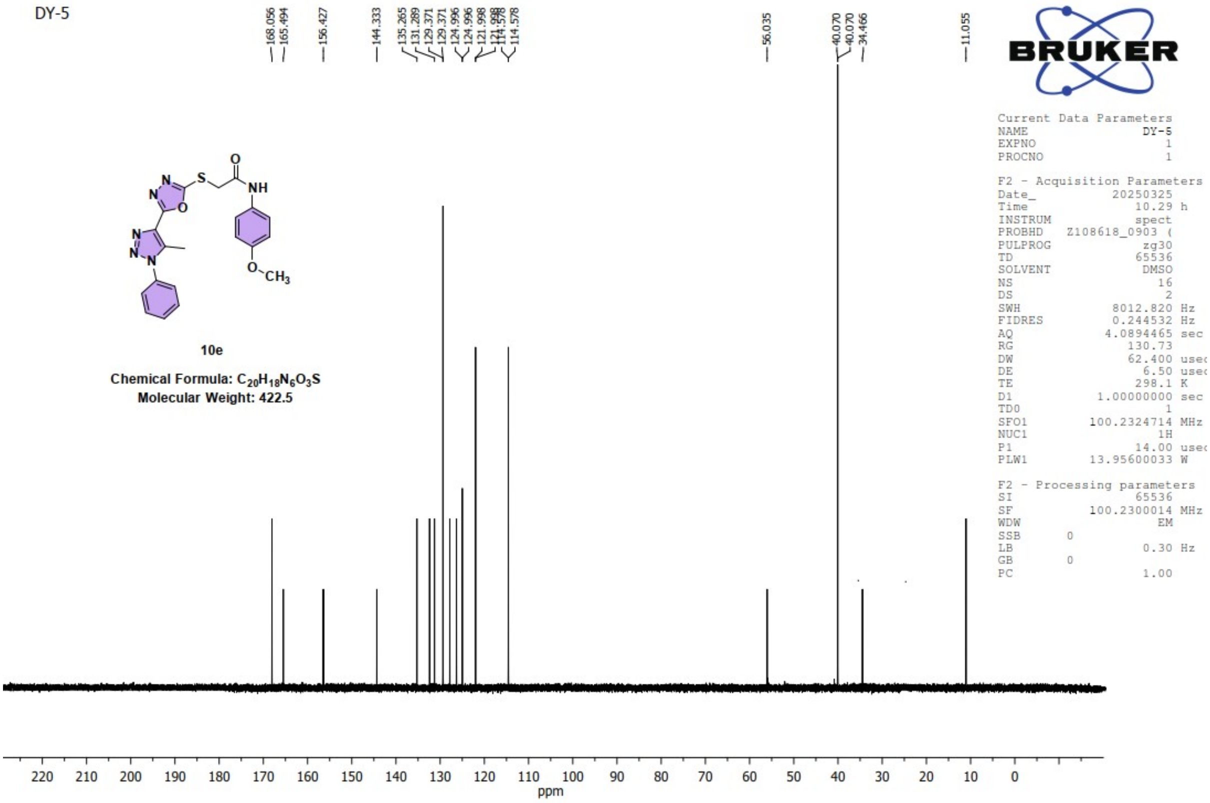

Fig. S45: ^{13}C NMR spectra of final compound 10f

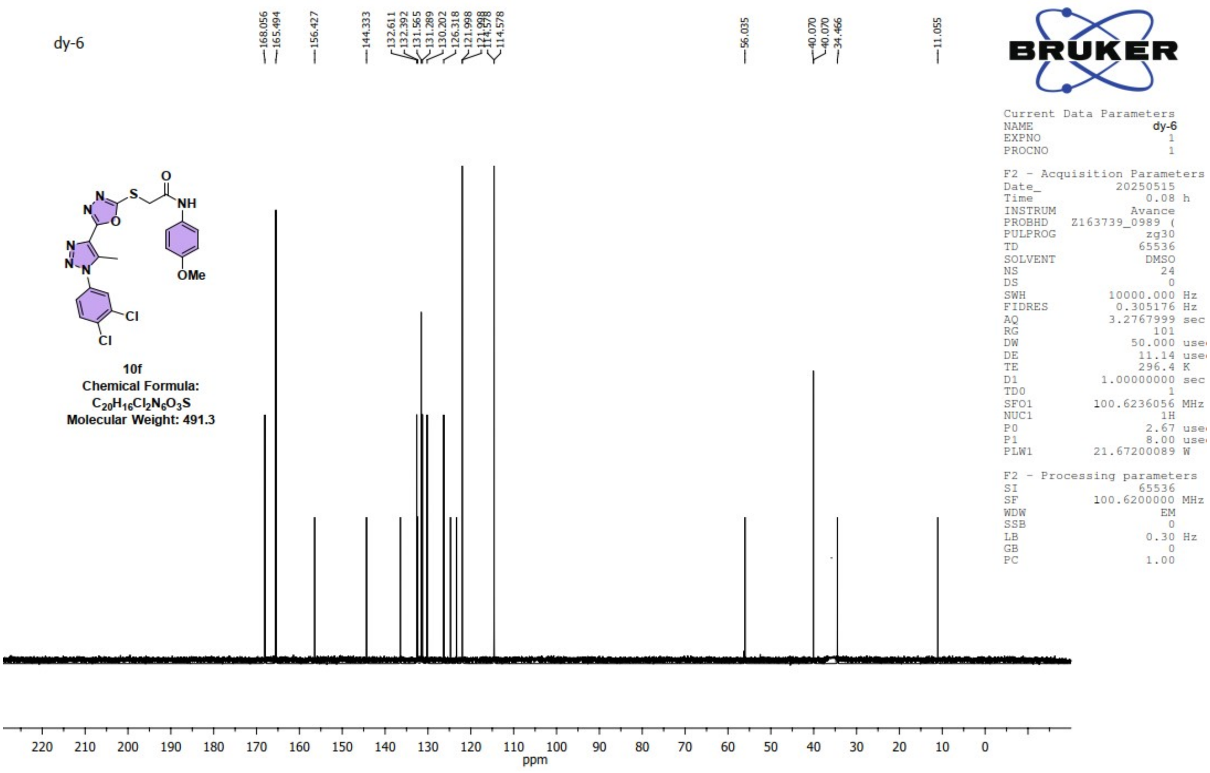

Fig. S46: ^{13}C NMR spectra of final compound 10g

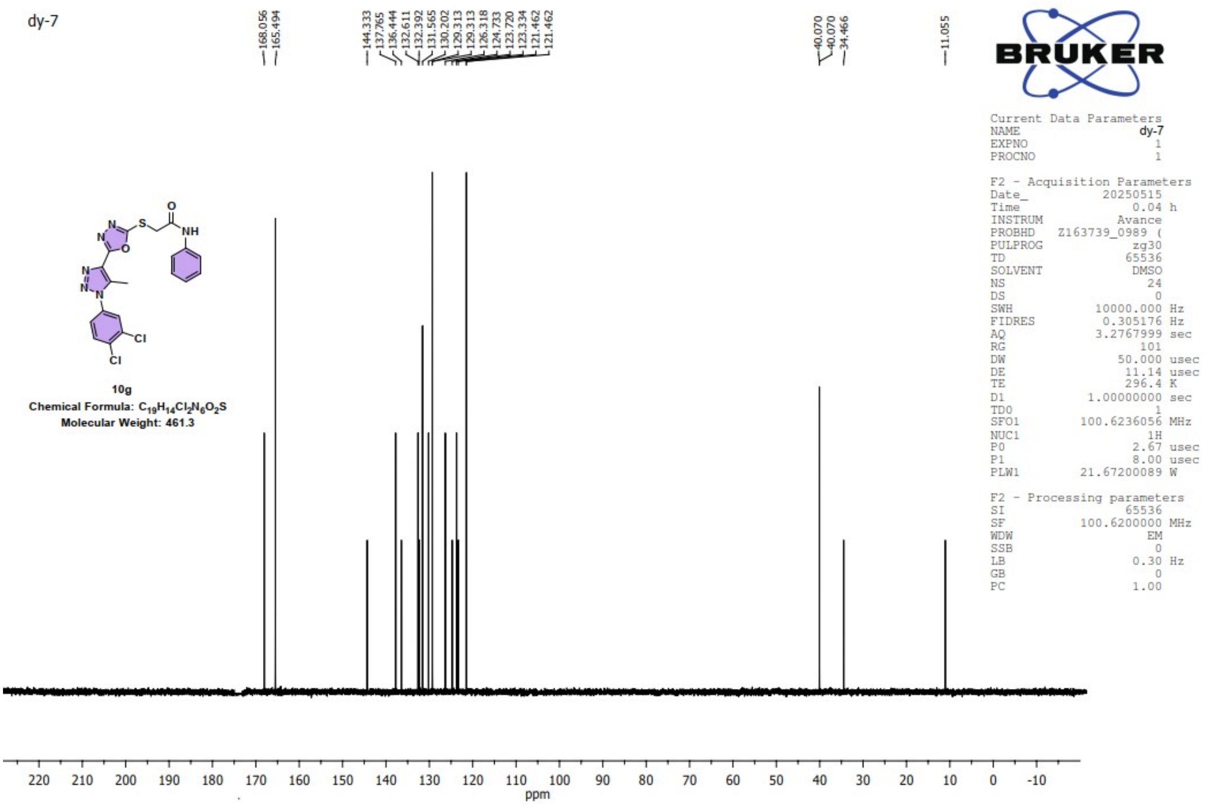

Fig. S47: ^{13}C NMR spectra of final compound 10h

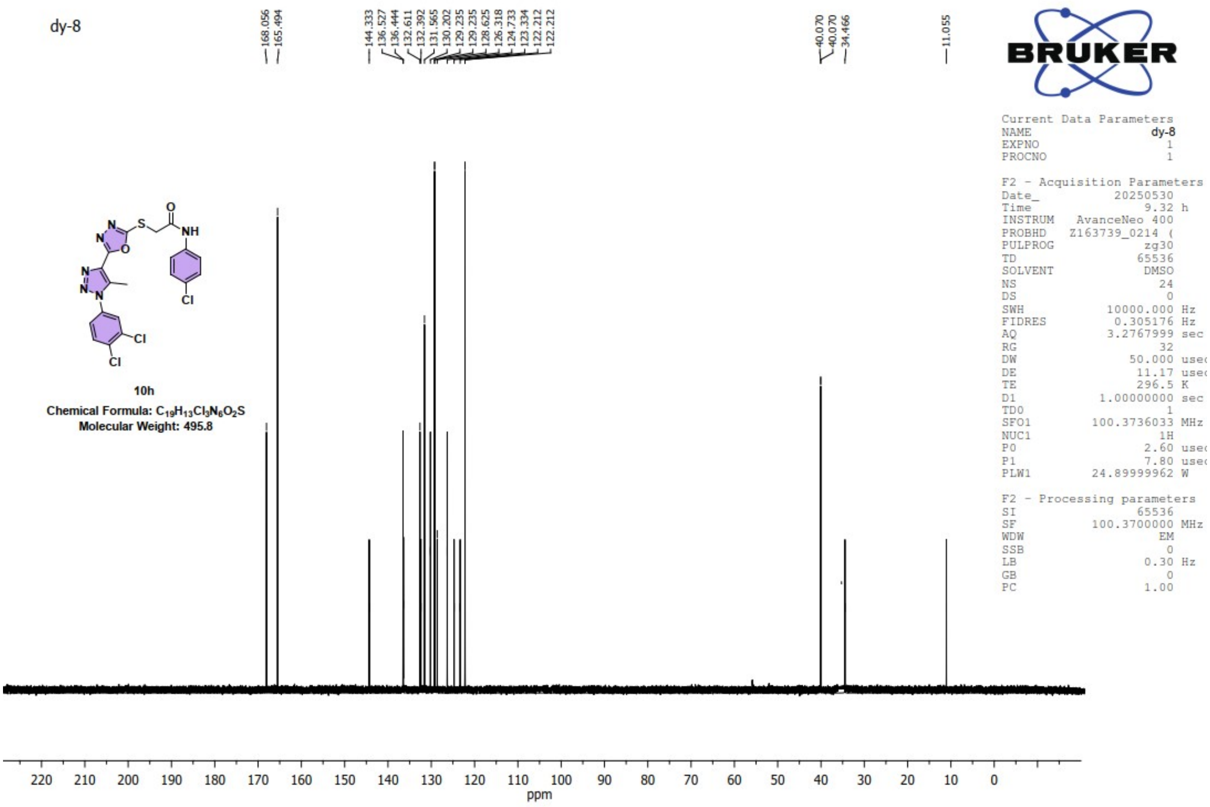

Fig. S48: ^{13}C NMR spectra of final compound 10i

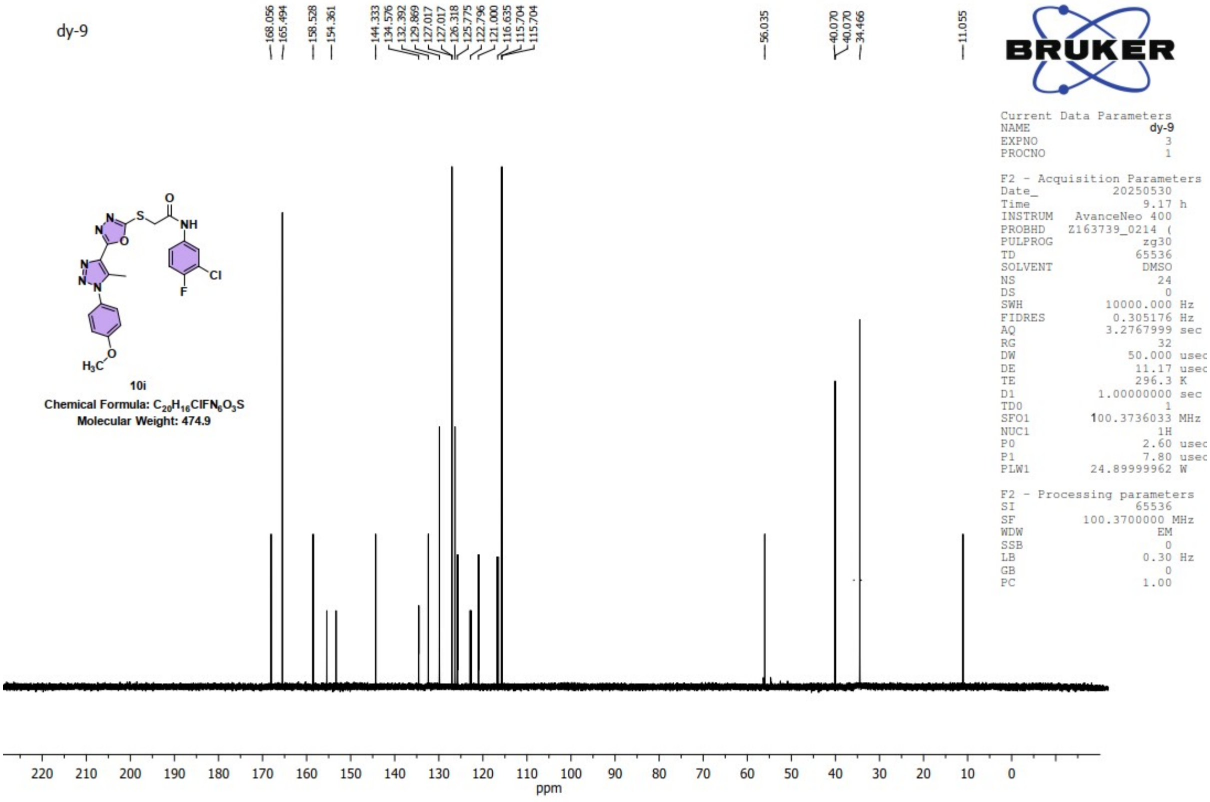

Fig. S49: ^{13}C NMR spectra of final compound 11a

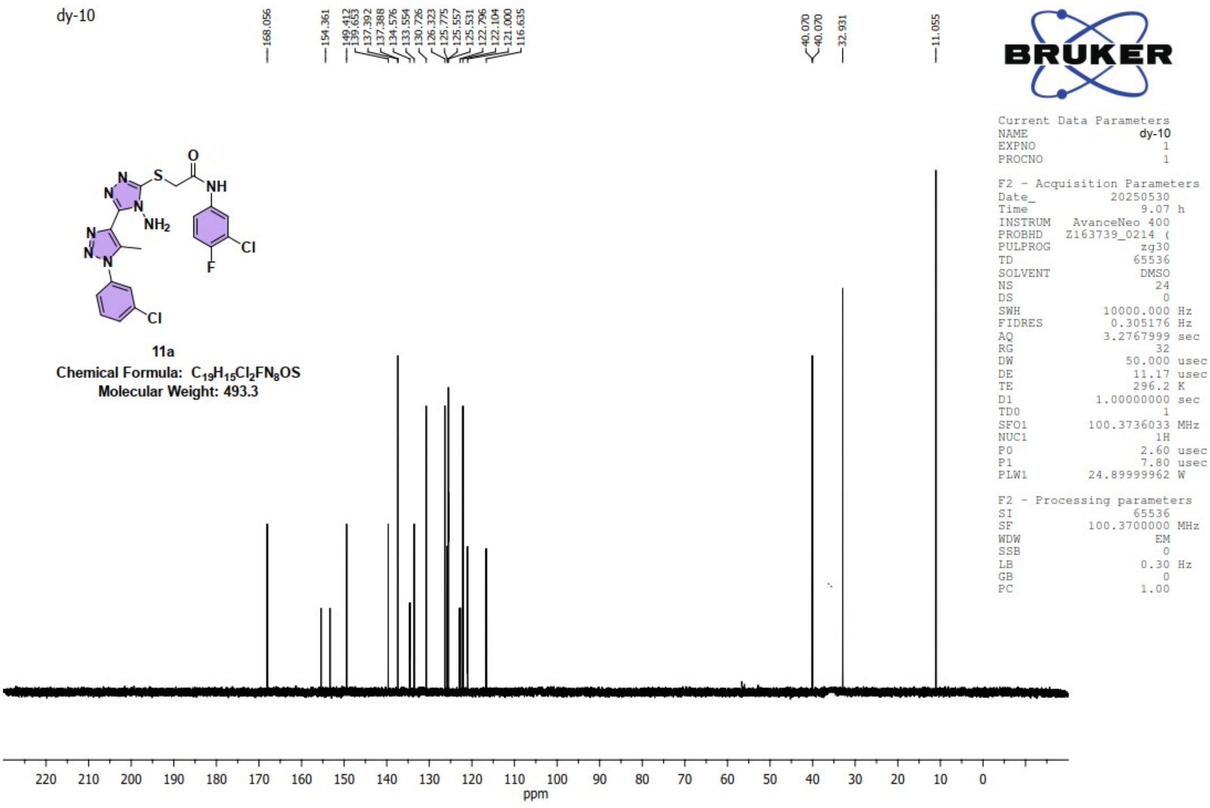

Fig. S50: ^{13}C NMR spectra of final compound 11b

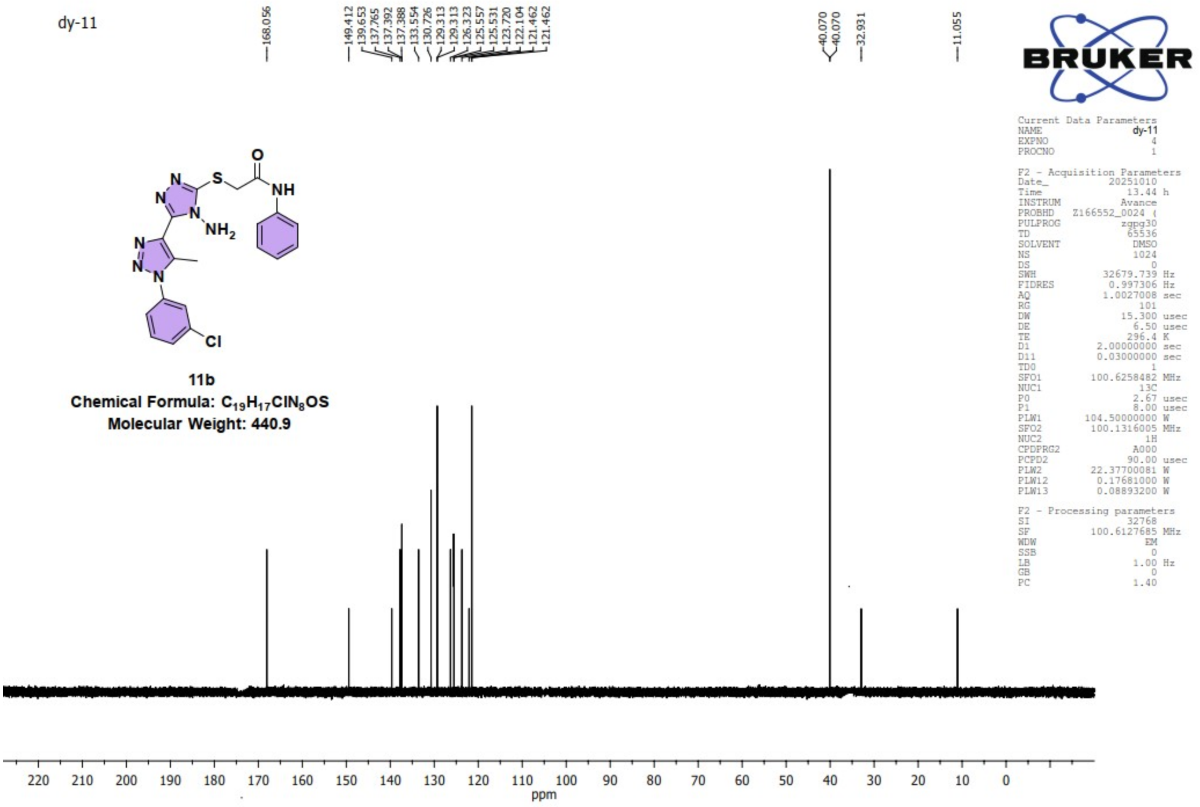

Fig. S51: ^{13}C NMR spectra of final compound 11c

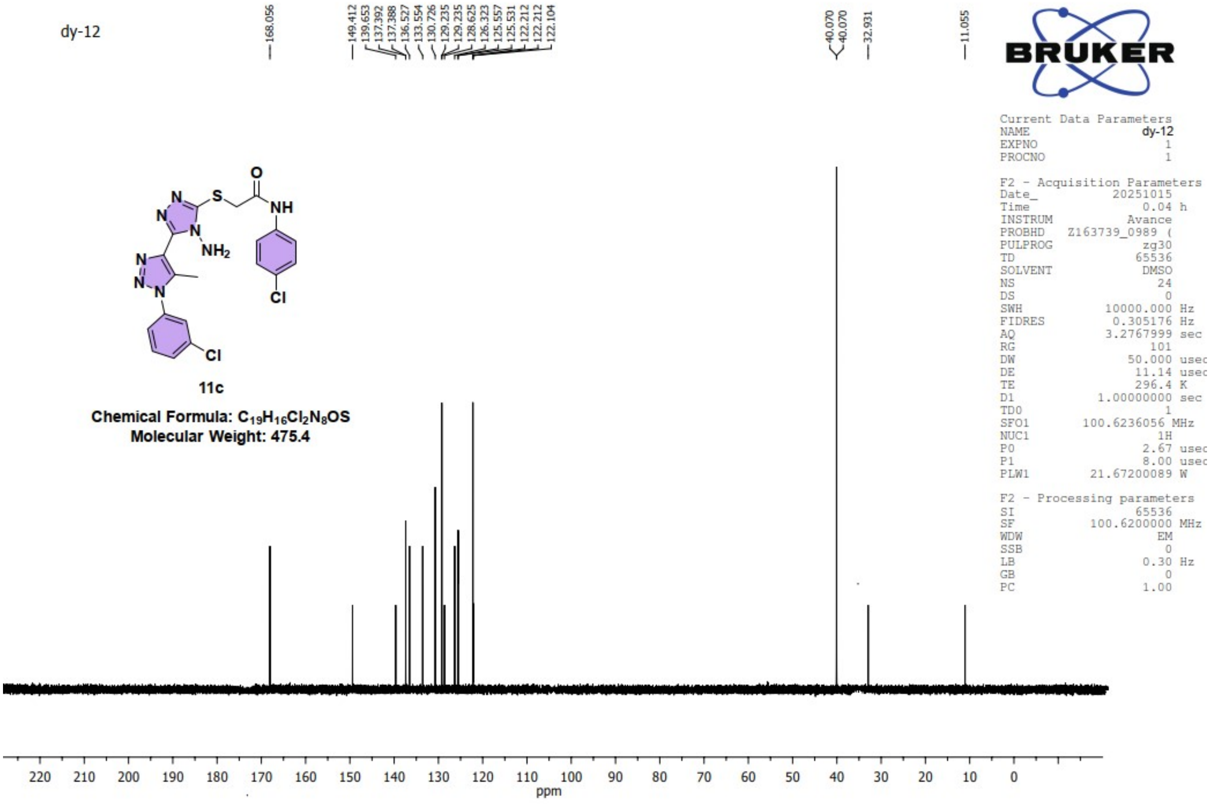

Fig. S52: ^{13}C NMR spectra of final compound 11d

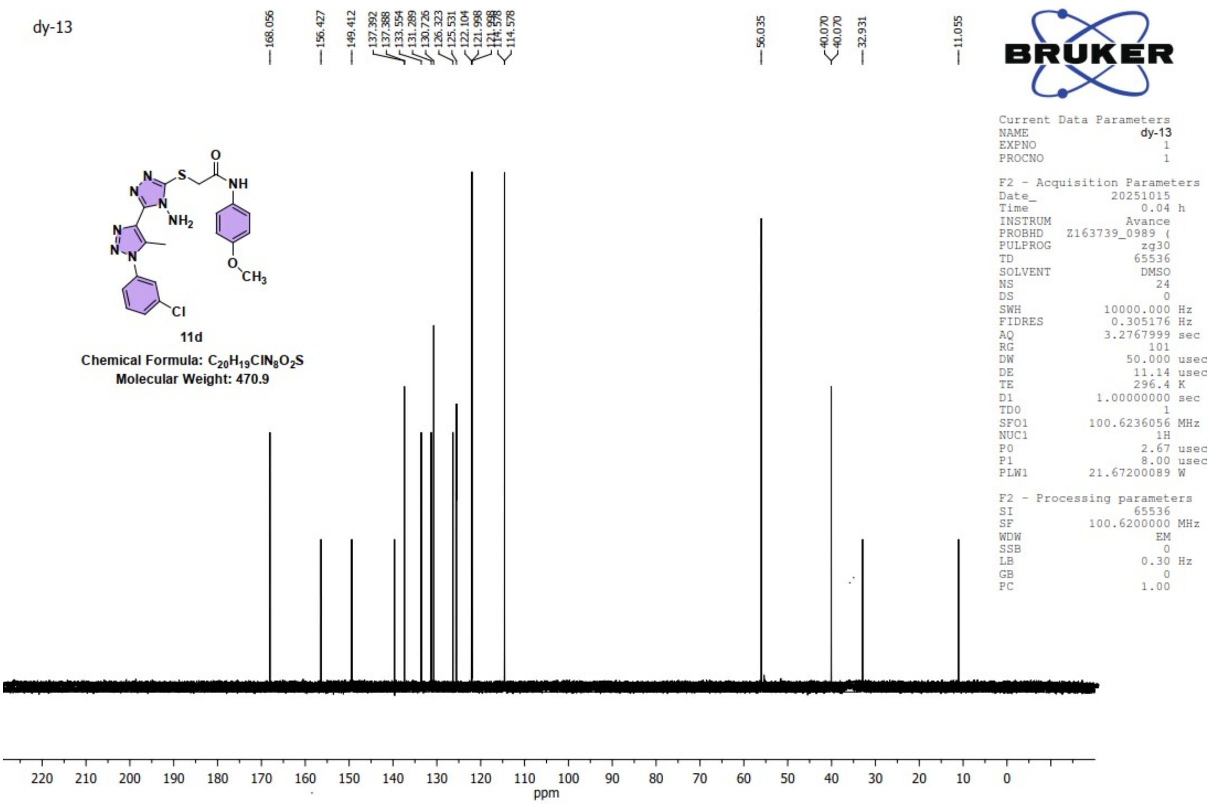

Table S1: Minimum inhibitory concentration (MIC; µg/mL) of the most active derivatives [b]

	MIC (Mean ± SEM) (µg/mL)					
	**Gram Positive Bacteria**		**Gram-Negative Bacteria**		**Fungi**	
**Comp.**	***S. aureus*** **ATCC 29213**	***B. subtilis*** **ATCC 6633**	***P. aeruginosa*** **ATCC 27853**	***E. coli*** **ATCC 25922**	***A. flavus*** **ATCC 46283**	***C.albicans*** **ATCC 10231**
10a	6.72 ± 0.31	2.96 ± 0.08	3.77 ± 0.03	5.51 ± 0.31	6.31 ± 0.05	9.92 ± 0.12
10b	4.31 ± 0.56	3.63 ± 0.02	4.21 ± 0.06	6.76 ± 0.12	7.90 ± 0.08	6.61 ± 0.21
10c	5.05 ± 0.21	5.90 ± 0.06	4.56 ± 0.05	9.72 ± 0.21	5.56 ± 0.02	5.53 ± 0.33
10d	5.25 ± 0.87	7.24 ± 0.06	6.21 ± 0.02	8.70 ± 0.27	7.21 ± 0.06	11.21 ± 0.15
10e	7.10 ± 0.64	9.61 ± 0.05	5.96 ± 0.01	5.69 ± 0.56	13.10 ± 0.05	13.16 ± 0.25
10f	6.63 ± 0.88	6.31 ± 0.09	7.92 ± 0.05	4.61 ± 0.78	10.90 ± 0.02	8.20 ± 0.20
10g	11.21 ± 0.81	3.23 ± 0.05	8.24 ± 0.05	3.90 ± 0.62	5.21 ± 0.07	6.64 ± 0.40
10h	16.34 ± 1.00	4.51 ± 0.08	4.67 ± 0.08	4.67 ± 0.25	7.89 ± 0.06	5.85 ± 0.16
10i	5.34 ± 0.57	10.23 ± 0.05	5.55 ± 0.06	9.12 ± 0.33	11.58 ± 0.08	19.22 ± 0.26
11a	3.85 ± 0.90	1.89 ± 0.05	2.41 ± 0.02	2.76 ± 0.40	3.31 ± 0.06	4.61 ± 0.35
11b	4.39 ± 0.53	8.56 ± 0.07	9.31 ± 0.08	8.52 ± 0.61	9.21 ± 0.09	7.54 ± 0.64
11c	8.54 ± 0.61	9.41 ± 0.08	16.22 ± 0.07	11.32 ± 0.50	8.54 ± 0.01	10.78 ± 0.42
11d	8.90 ± 0.72	12.50 ± 0.06	6.33 ± 0.08	7.61 ± 0.84	4.34 ± 0.03	12.25 ± 0.29
Ciprofloxacin	5.85 ± 0.13	2.90 ± 0.02	2.90 ± 0.04	2.90 ± 0.25	-	-
Griseofulvin	-	-	-	-	4.25 ± 0.05	12.5 ± 0.15

[a]Data were expressed as mean ± SD of three experiments, - represents no activity[b]

Table S2. The antitumor activities of the tested compounds expressed as IC50 values and compared with reference standard drugs evaluated on breast and liver cancer cell lines[a]

Comp.	IC50 values (µg/mL) against tumor cell lines	
	MCF-7	HepG2
10a	151.3 ± 0.5	29.1 ± 0.5
10b	16.1 ± 0.5	>200
10c	6.8± 0.4	21 ± 0.6
10d	96.6 ± 0.5	175 ± 0.2
10e	>200	22.9 ± 0.5
10f	49.6 ± 0.5	>200
10g	85.2 ± 0.5	39 ± 0.5
10h	>200	164 ± 0.8
10i	69.2 ± 0.5	>200
11a	5.8 ± 0.2	4.2 ± 0.7
11b	134.9 ± 0.5	36.9 ± 0.5
11c	109.8 ± 0.5	89.1 ± 0.2
11d	>200	>200
Pyridyl cyanoguanidine	6.5 ± 0.5	5.1 ± 0.2

Data were expressed as mean ± SD of three experiments[a]

Table S3: Molecular docking results of synthesized pyrazole-oxadiazole hybrids (10a-i, 11a-d) and reference drugs (Erlotinib and Doxorubicin) against EGFR kinase domain (PDB: 3W2Q), displaying binding affinity scores and critical amino acid interactions.

Compound	Docking Score (Kcal/mol)	Hydrophobic Interactions	Polar Interactions	Hydrogen Bonds	Other Interactions
10a	-5.186	VAL726, PHE723, MET766, LEU718, ALA743, MET793, LEU792, MET790, LEU844, ILE759, GLY796, GLY857	THR854, GLN791	LYS745 (H-bond donor/acceptor)	GLU758 (charged negative), GLU762 (charged negative), ASP855 (charged negative), LYS745 (charged positive)
10b	-5.394	LEU718, ALA743, PRO794, MET793, LEU792, MET790, VAL726, PHE723, ILE759, GLY857, MET766, LEU844	THR854	LYS745 (H-bond donor/acceptor)	GLU762 (charged negative), ASP855 (charged negative), LYS745 (charged positive), π-π stacking interaction with PHE723
10c	-5.570	ILE759, ALA755, ALA743, LEU718, VAL726, PHE723, MET766, LEU844, MET790, LEU792, MET793, PRO794, GLY796	GLN791	LYS745 (multiple H-bonds with ligand carbonyl and nitrogen)	GLU758 (charged negative), ASP855 (charged negative), GLU762 (charged negative), LYS745 (charged positive)
10d	-5.713	LEU718, VAL726, PRO794, MET793, LEU792, MET790, ALA743, MET766, LEU844,	GLN791, ASN842, THR854	LYS745 (H-bond with nitrogen), ASP855 (H-bond with NH group)	ARG841 (charged positive), ASP837 (charged negative), ASP855 (charged negative), GLU762 (charged negative), LYS745 (charged positive)

		GLY796			
10e	-5.400	PHE723, VAL726, LEU718, ALA755, ALA743, ILE759, GLY796, PRO794, MET793, LEU792, MET790, LEU844, GLY857, PHE856	GLN791, THR854	LYS745 (H-bond with nitrogen), GLU762 (H-bond with ligand)	GLU758 (charged negative), GLU762 (charged negative), ASP855 (charged negative), LYS745 (charged positive)
10f	-5.572	LEU844, MET790, LEU792, MET793, PRO794, LEU718, GLY796, CYS797, ALA743, VAL726, MET766	GLN791, ASN842, THR854	THR854 (H-bond with carbonyl oxygen), MET793 (H-bond interaction)	ARG841 (charged positive), ASP855 (charged negative), GLU762 (charged negative), LYS745 (charged positive), Halogen bonds with Cl substituents
10g	-5.707	ILE759, ALA755, GLY857, PHE856, LEU844, MET790, LEU792, MET793, PRO794, GLY796, LEU718, ALA743, MET766, PHE723, VAL726	GLN791, THR854	LYS745 (H-bond with nitrogen), GLU762 (H-bond with NH group)	GLU758 (charged negative), GLU762 (charged negative), ASP855 (charged negative), LYS745 (charged positive), Halogen bonds with Cl substituents
10h	-5.622	ILE759, ALA755, GLY857, PHE856, LEU844, MET790, LEU792,	GLN791, THR854	LYS745 (H-bond with nitrogen), GLU762 (H-bond with NH group)	GLU758 (charged negative), GLU762 (charged negative), ASP855 (charged negative), LYS745 (charged positive), Halogen bonds with Cl

		MET793, PRO794, GLY796, LEU718, ALA743, MET766, PHE723, VAL726			substituents on both phenyl rings
10i	-5.779	PRO877, GLY857, ALA743, VAL726, LEU844, LEU718, GLY796, PRO794, MET793, LEU792, MET790	GLN791, THR854, ASN842	LYS745 (H-bond with nitrogen), ASP855 (multiple H-bonds), MET793 (H-bond interaction)	LYS875 (charged positive), ARG858 (charged positive), GLU762 (charged negative), ASP837 (charged negative), ASP855 (charged negative), LYS745 (charged positive), Halogen bonds with F and Cl substituents
11a	-5.330	LEU 792, LEU 718, LEU 844, ILE 759, VAL 726, PHE 723, MET 790, MET 793, PRO 794, ALA 743, CYS 797, GLY 796, GLY 857	THR 854	H_2N group with surrounding residues, Potential bonds with ASP 855, THR 854, LYS745 (multiple H-bonds with ligand nitrogen)	GLU762 (charged negative), ASP855 (charged negative), LYS745 (charged positive)
11b	-5.530	LEU 792, LEU 718, LEU 844, ILE 759, ALA 755, PHE 723, VAL 726, MET 790, MET 793, ALA 743, GLY 796, GLY 857	THR 854, GLN 791	LYS745 (multiple H-bonds with ligand carbonyl and nitrogen)	GLU762 (charged negative), ASP855 (charged negative), GLU758 (charged negative), LYS745 (charged positive)
11c	-5.762	LEU 792, LEU 718, LEU 844, VAL 726, PHE 723, PHE 856, MET 790, MET 793, MET 766, PRO 794, ALA 743, CYS 797, GLY 796, GLY 857	THR 854, GLN 791	GLU762 (H-bond with NH group)	GLU762 (charged negative), ASP855 (charged negative), LYS745 (charged positive)

11d	-5.099	LEU 792, LEU 718, LEU 844, VAL 726, PHE 723, PHE 856, MET 790, MET 793, MET 766, PRO 794, ALA 743, CYS 797, GLY 796, GLY 857	THR 854, GLN 791	LYS745 (multiple H-bonds with ligand carbonyl and nitrogen)	GLU762 (charged negative), ASP855 (charged negative), LYS728 (charged positive), LYS745 (charged positive)
Erlotinib	-5.292	LEU 792, LEU 718, LEU 844, VAL 726, PHE 723, PHE 856, MET 790, MET 793, MET 766, PRO 794, ALA 743, CYS 775	THR 854, GLN 791, ASN 842	LYS745 (multiple H-bonds with Oxygen)	GLU762 (charged negative), ASP855 (charged negative), LYS745 (charged positive)
Doxorubicin	-6.588	LEU 792, LEU 718, LEU 844, VAL 726, PHE 723, PHE 856, MET 766, ALA 743, GLY 796, GLY 857	THR 854, GLN 791, ASN 842	LYS745 (multiple H-bonds with Oxygen atom), ASN 842 (H-bond with Oxygen atom), GLU 762 (H-bond with nitrogen atom)	GLU762 (charged negative), ASP855 (charged negative), LYS745 (charged Positive), ARG841 (charged negative)

Molecular Docking (3W2Q)

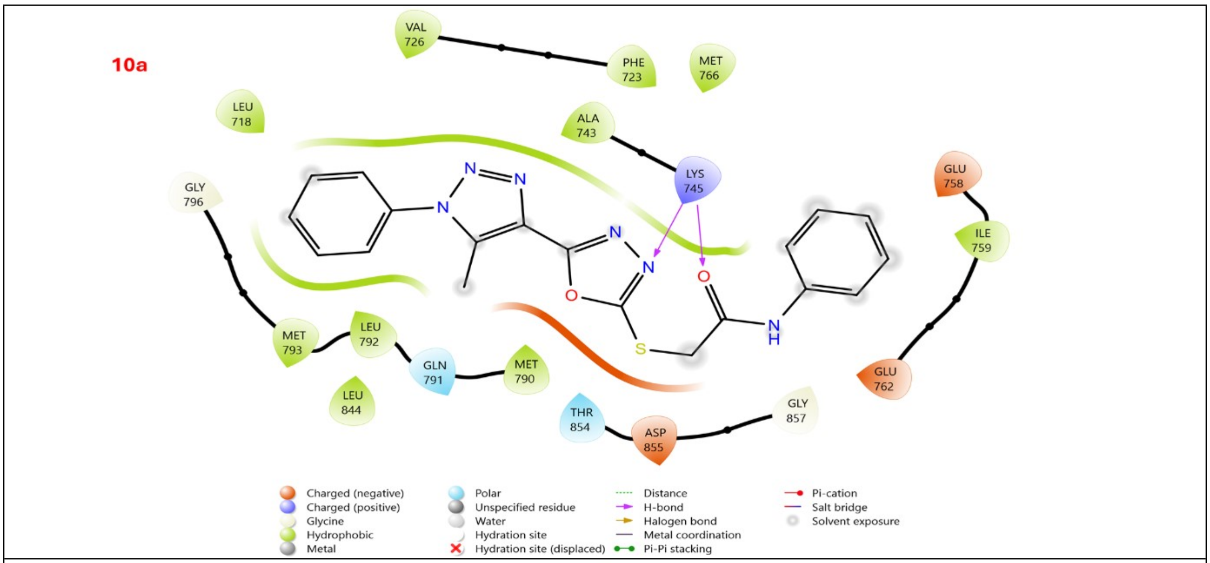

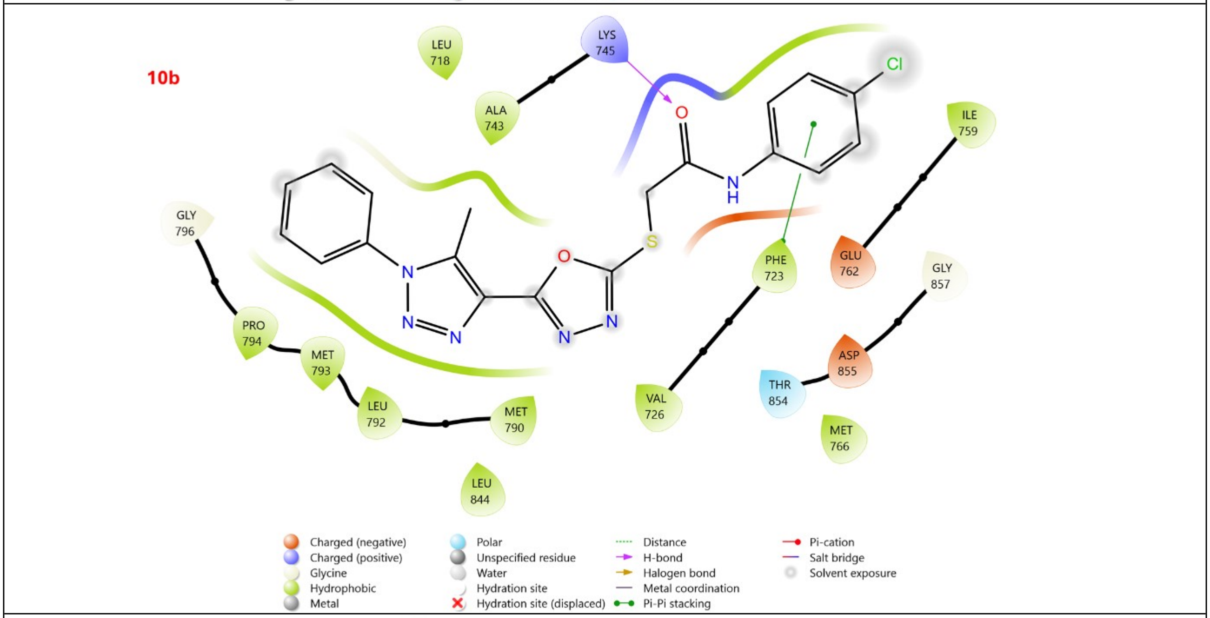

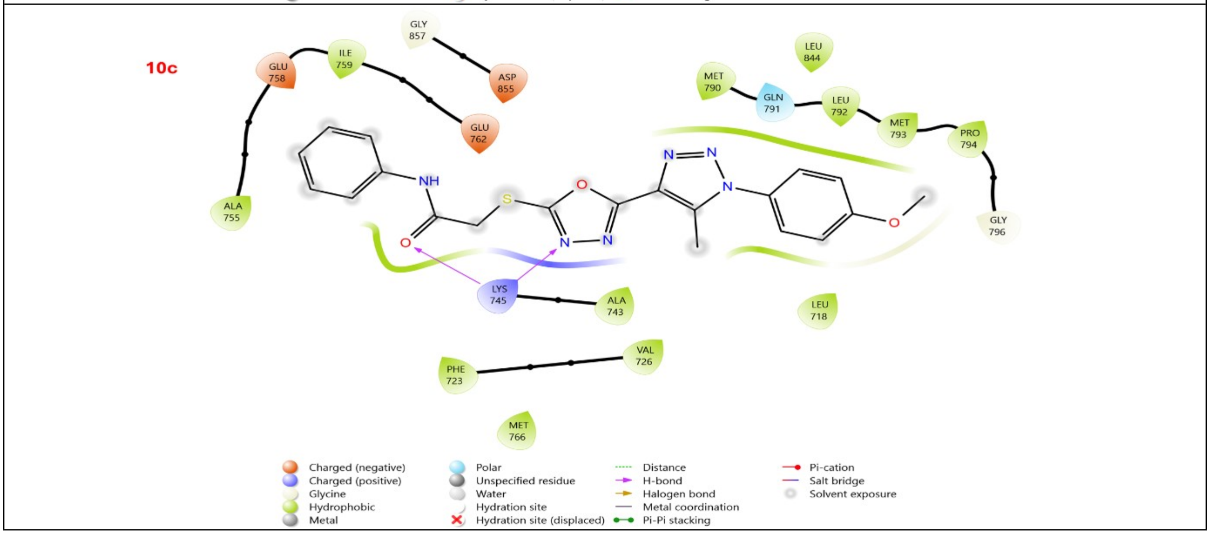

10d

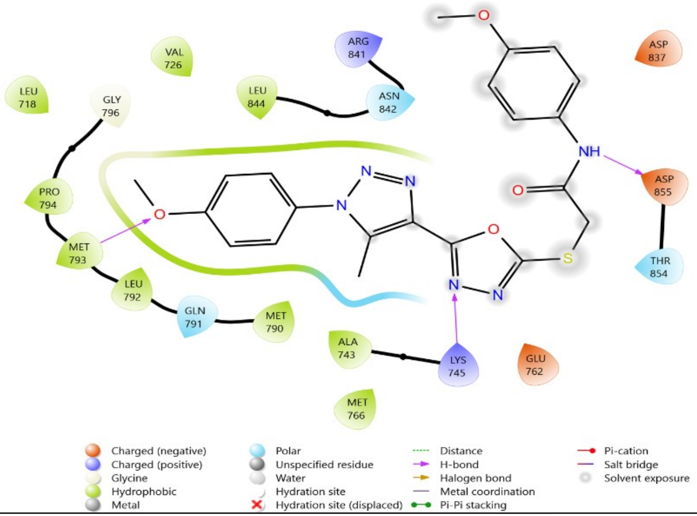
VAL 726
ARG 841
ASP 837
LEU 718
GLY 796
LEU 844
ASN 842
NH
ASP 855
O
N
N
N
PRO 794
O
N
O
MET 793
S
THR 854
N
N
LEU 792
GLN 791
MET 790
ALA 743
LYS 745
GLU 762
MET 766
Charged (negative)
Charged (positive)
Glycine
Hydrophobic
Metal
Polar
Unspecified residue
Water
Hydration site
Hydration site (displaced)
Distance
H-bond
Halogen bond
Metal coordination
Pi-Pi stacking
Pi-cation
Salt bridge
Solvent exposure

10e

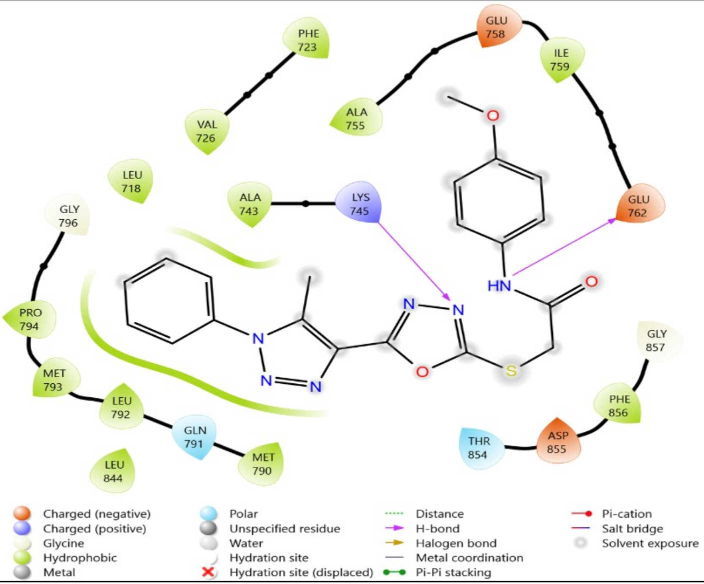
PHE 723
GLU 758
ILE 759
ALA 755
VAL 726
O
LEU 718
ALA 743
LYS 745
GLU 762
GLY 796
HN
O
N
N
PRO 794
N
GLY 857
N
N
O
S
MET 793
LEU 792
PHE 856
GLN 791
THR 854
ASP 855
MET 790
LEU 844
Charged (negative)
Charged (positive)
Glycine
Hydrophobic
Metal
Polar
Unspecified residue
Water
Hydration site
Hydration site (displaced)
Distance
H-bond
Halogen bond
Metal coordination
Pi-Pi stacking
Pi-cation
Salt bridge
Solvent exposure

10f

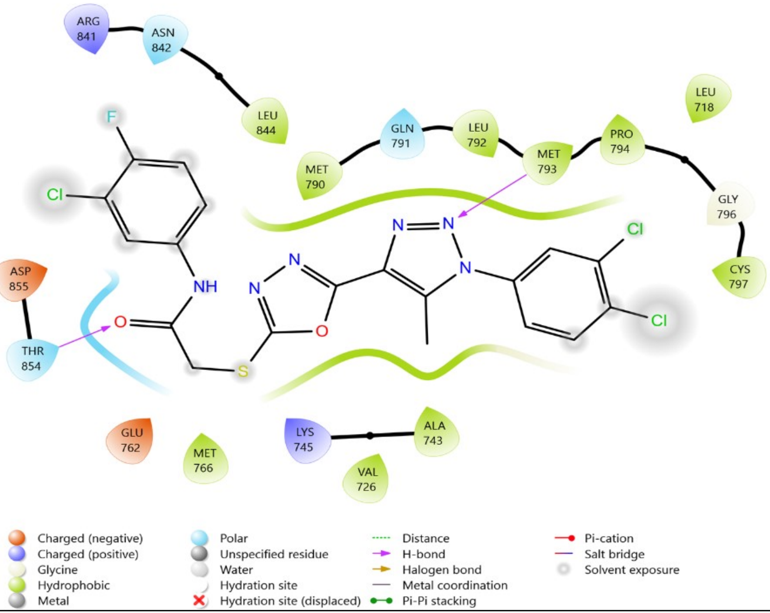
ARG 841
ASN 842
LEU 718
F
LEU 844
GLN 791
LEU 792
MET 793
PRO 794
MET 790
GLY 796
Cl
N
N
Cl
N
N
NH
N
CYS 797
ASP 855
O
Cl
O
THR 854
S
GLU 762
MET 766
LYS 745
ALA 743
VAL 726
Charged (negative)
Charged (positive)
Glycine
Hydrophobic
Metal
Polar
Unspecified residue
Water
Hydration site
Hydration site (displaced)
Distance
H-bond
Halogen bond
Metal coordination
Pi-Pi stacking
Pi-cation
Salt bridge
Solvent exposure

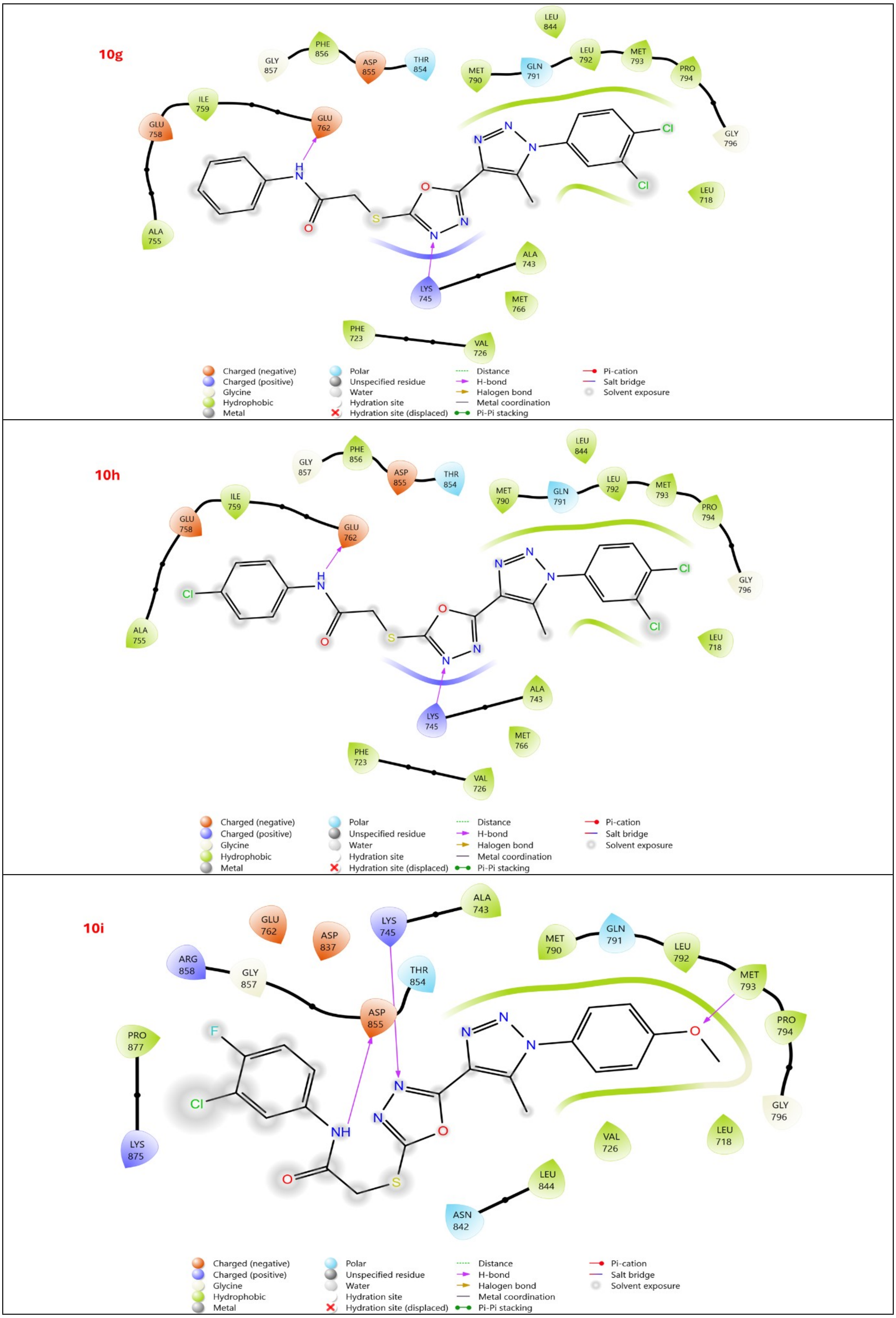
10g
LEU 844
PHE 856
GLY 857
ASP 855
THR 854
MET 790
GLN 791
LEU 792
MET 793
PRO 794
ILE 759
GLU 758
GLU 762
GLY 796
LEU 718
ALA 755
ALA 743
LYS 745
MET 766
PHE 723
VAL 726
H N O S O N N N N N Cl Cl
Charged (negative)
Charged (positive)
Glycine
Hydrophobic
Metal
Polar
Unspecified residue
Water
Hydration site
Hydration site (displaced)
Distance
H-bond
Halogen bond
Metal coordination
Pi-Pi stacking
Pi-cation
Salt bridge
Solvent exposure
10h
LEU 844
PHE 856
GLY 857
ASP 855
THR 854
MET 790
GLN 791
LEU 792
MET 793
PRO 794
ILE 759
GLU 758
GLU 762
GLY 796
LEU 718
ALA 755
ALA 743
LYS 745
MET 766
PHE 723
VAL 726
Cl H N O S O N N N N N Cl Cl
Charged (negative)
Charged (positive)
Glycine
Hydrophobic
Metal
Polar
Unspecified residue
Water
Hydration site
Hydration site (displaced)
Distance
H-bond
Halogen bond
Metal coordination
Pi-Pi stacking
Pi-cation
Salt bridge
Solvent exposure
10i
GLU 762
ASP 837
LYS 745
ALA 743
MET 790
GLN 791
LEU 792
MET 793
ARG 858
GLY 857
THR 854
ASP 855
PRO 794
PRO 877
LYS 875
GLY 796
LEU 718
VAL 726
LEU 844
ASN 842
F Cl NH O S N N O N N N O
Charged (negative)
Charged (positive)
Glycine
Hydrophobic
Metal
Polar
Unspecified residue
Water
Hydration site
Hydration site (displaced)
Distance
H-bond
Halogen bond
Metal coordination
Pi-Pi stacking
Pi-cation
Salt bridge
Solvent exposure

11a

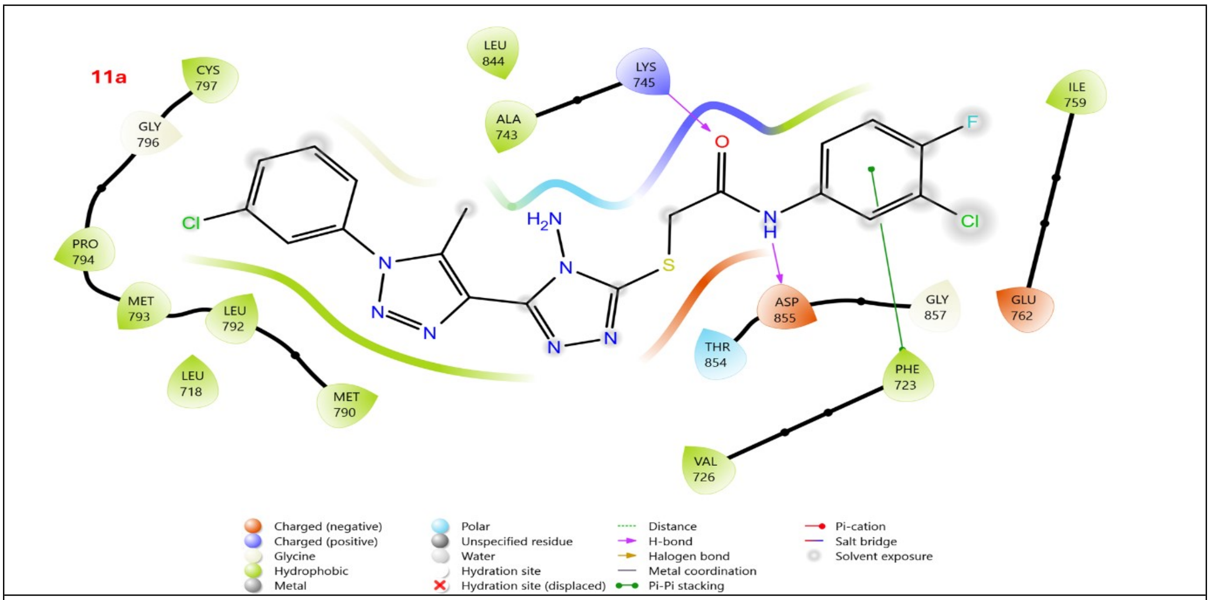
CYS 797
GLY 796
PRO 794
MET 793
LEU 792
LEU 718
MET 790
LEU 844
ALA 743
LYS 745
ASP 855
THR 854
GLY 857
PHE 723
VAL 726
ILE 759
GLU 762
Charged (negative)
Charged (positive)
Glycine
Hydrophobic
Metal
Polar
Unspecified residue
Water
Hydration site
Hydration site (displaced)
Distance
H-bond
Halogen bond
Metal coordination
Pi-Pi stacking
Pi-cation
Salt bridge
Solvent exposure

11b

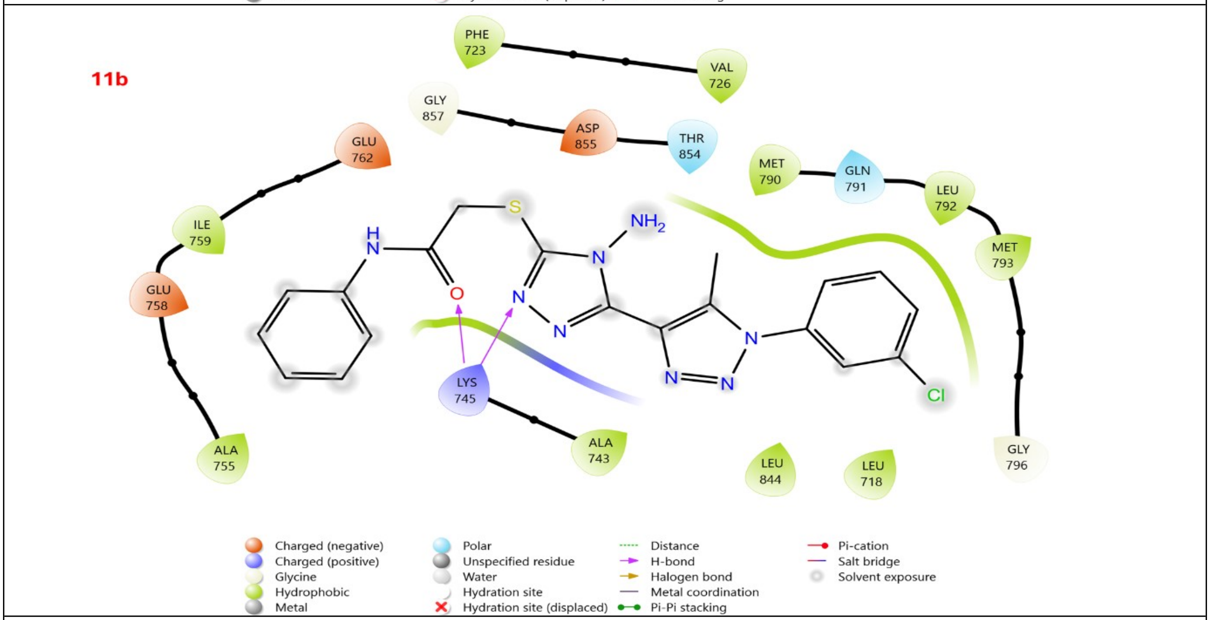
PHE 723
VAL 726
GLY 857
ASP 855
THR 854
GLU 762
ILE 759
GLU 758
ALA 755
LYS 745
ALA 743
MET 790
GLN 791
LEU 792
MET 793
GLY 796
LEU 844
LEU 718
Charged (negative)
Charged (positive)
Glycine
Hydrophobic
Metal
Polar
Unspecified residue
Water
Hydration site
Hydration site (displaced)
Distance
H-bond
Halogen bond
Metal coordination
Pi-Pi stacking
Pi-cation
Salt bridge
Solvent exposure

11c

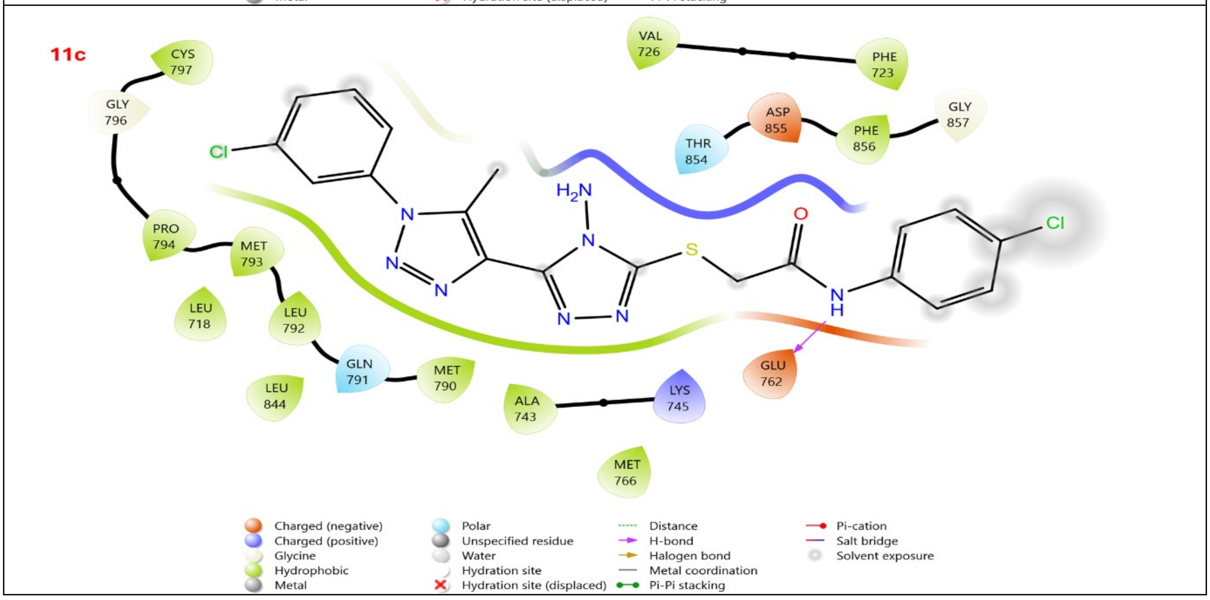
CYS 797
GLY 796
PRO 794
MET 793
LEU 718
LEU 792
GLN 791
MET 790
LEU 844
ALA 743
LYS 745
MET 766
VAL 726
PHE 723
ASP 855
THR 854
PHE 856
GLY 857
GLU 762
Charged (negative)
Charged (positive)
Glycine
Hydrophobic
Metal
Polar
Unspecified residue
Water
Hydration site
Hydration site (displaced)
Distance
H-bond
Halogen bond
Metal coordination
Pi-Pi stacking
Pi-cation
Salt bridge
Solvent exposure

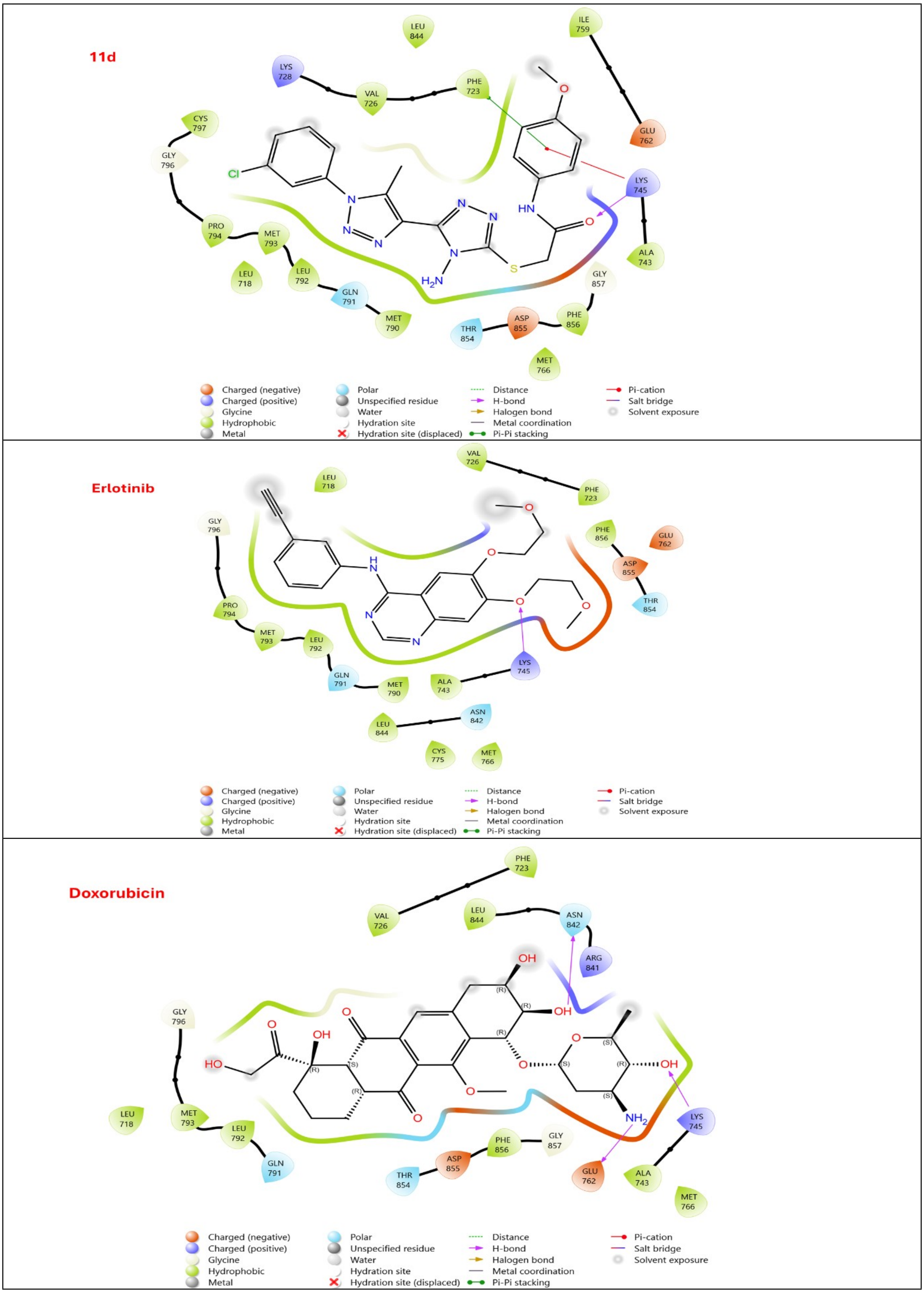

Figure S53. 2D and 3D Molecular docking diagram 3W2Q

Table S4: Molecular docking results of synthesized pyrazole-oxadiazole hybrids (10a-i, 11a-d) and reference drugs (Ciprofloxacin and Griseofulvin) against EGFR kinase domain (PDB: 4QGG), displaying binding affinity scores and critical amino acid interactions.

Compound	Docking Score (Kcal/mol)	Hydrophobic Interactions	Polar Interactions	Hydrogen Bonds	Other Interactions
10a	-5.522	PRO10, VAL51, LEU52, ILE47, ILE143, PRO38, PHE66	SER13, SER69, THR16	ARG92 (H-bond donor/acceptor with O)	ASP91(charged negative), GLU11 (charged negative), GLU37 (charged negative), ARG36(charged positive), ARG48 (charged positive), LYS15 (charged positive), LYS144 (charged positive), ARG92 (charged positive)
10b	-5.072	PRO10, VAL51, LEU52, LEU65, ILE47, ILE143, PHE66	SER13, SER69, THR16, ASN145	ASN145 (H-bond carbonyl O)	GLU11 (charged negative), GLU37 (charged negative), ARG36 (charged positive), ARG48 (charged positive), LYS15 (charged positive), LYS144 (charged positive)
10c	-5.607	PRO38, VAL51, LEU52, ILE47, PHE66, TYR100, PHE159	SER69, GLN101	GLU37 (H-bond with N); GLN101 (H-bond with N)	ARG36 (charged positive), ARG48 (charged positive), ARG70 (charged positive), ARG105 (charged positive), GLU37 (charged negative), GLU62 (charged negative)
10d	-4.661	PRO38, VAL51, LEU52, PHE66, TYR93, TYR100, PHE159	SER96, SER97, GLN101	GLN101 (H-bond with O)	ARG36 (charged positive), ARG48 (charged positive), ARG70 (charged positive), ARG105 (charged positive), GLU37 (charged negative), GLU45 (charged negative), GLU62 (charged negative)
10e	-5.198	PRO10, VAL51, LEU52, ILE47, LEU65, PHE66, ILE143	SER13, SER69, THR16	ASP91 (H-bond with -NH), ARG92 (H-bond with Carbonyl C), GLY12 (H-bond with O)	ARG36(charged positive), ARG48 (charged positive), LYS15(charged positive), LYS144 (charged positive), GLU11(charged negative), GLU37 (charged negative)
10f	-5.785	ILE47, VAL51, LEU52, PHE66, ILE143, PRO38	SER13, SER69, THR16,	ARG48 (H bond with N atom) ARG92 (H-bond with carbonyl C)	ARG36 (charged positive), ARG48 (charged positive), LYS15 (charged positive), LYS144 (charged positive), GLU11(charged negative), GLU37 (charged negative)
10g	-5.017	PRO38, ILE47, VAL51, LEU52, PHE66, TYR100, PHE159	SER69, SER 96, GLN101	GLU37 (H bond with -NH atom) GLN101 (H-bond with N atom)	ASP91(charged negative), GLU11 (charged negative), GLU37 (charged negative), GLU62 (charged negative), ARG36 (charged positive), ARG48 (charged positive), ARG70 (charged positive), ARG92 (charged positive), ARG105 (charged positive)
10h	-4.784	PHE66, TYR100, PHE159	SER 96, SER 97, GLN101	GLN101 (H-bond with N atom)	ASP156 (charged negative), GLU11 (charged negative), GLU37 (charged negative), GLU62 (charged negative),

					ARG70 (charged positive), ARG92 (charged positive), ARG105 (charged positive), ARG151 (charged positive),
10i	-5.460	PRO10, PRO38, ILE47, VAL51, LEU52, LEU65, PHE66	SER13, THR16, SER69, ASN145	ARG48 (H bond with N atom), ASN145 (H bond with O atom)	ASP91 (charged negative), GLU11 (charged negative), GLU37 (charged negative), ARG48 (charged positive), ARG92 (charged positive), ARG36 (charged positive), LYS15 (charged positive), LYS144 (charged positive),
11a	-5.242	PHE66, TYR100, TYR93, ILE143	SER13, THR16, SER96, SER97, GLN101, ASN145	GLN101 (H-bond with N atom)	GLU11 (charged negative), GLU37 (charged negative), ARG70 (charged positive), ARG92 (charged positive), ARG105 (charged positive), LYS15 (charged positive), LYS144 (charged positive),
11b	-5.890	PRO10, ILE143, TYR100, TYR93, PHE66	SER13, THR16, SER96, SER97, GLN101, ASN145	GLN101 (H-bond with O atom), GLU11 (H-bond with -NH2 atom)	GLU11 (charged negative), GLU37 (charged negative), ARG70 (charged positive), ARG92 (charged positive), ARG105 (charged positive), LYS15 (charged positive), LYS144 (charged positive),
11c	-4.918	PRO10, ILE143, TYR100, TYR93, PHE66	SER13, THR16, SER96, SER97, GLN101, ASN145	ASN145 (H-bond with O atom)	GLU11 (charged negative), GLU37 (charged negative), ASP91 (charged negative), ARG70 (charged positive), ARG92 (charged positive), ARG36 (charged positive), LYS15 (charged positive), LYS144 (charged positive), ARG48 (charged positive)
11d	-5.672	ILE143, TYR100, TYR93, PHE66, PHE159	SER13, THR16, SER96, SER97, GLN101, ASN145	GLN101 (H-bond with O atom), GLU11 (H-bond with -NH2 atom)	GLU11 (charged negative), GLU37 (charged negative), ARG70 (charged positive), ARG92 (charged positive), LYS15 (charged positive), LYS144 (charged positive), ARG105 (charged positive),
Griseofulvin	-3.817	PRO38, ILE47, VAL51, LEU52, LEU65, TYR93	SER69, SER96,	ARG48 (H bond with O atom),	ASP91 (charged negative), GLU37 (charged negative), ARG70 (charged positive), ARG92 (charged positive), ARG36 (charged positive), ARG48 (charged positive
Ciprofloxacin	-6.747	PRO38, VAL51, LEU52, PHE66, TYR100	SER69, SER96, SER97,	ARG48 (H bond with O atom), ARG92 (H-bond	GLU37 (charged negative), ARG70 (charged positive), ARG92 (charged positive),

			GLN101,	with O)	ARG36 (charged positive), ARG48 (charged positive

Molecular Docking (4QGG)

10a

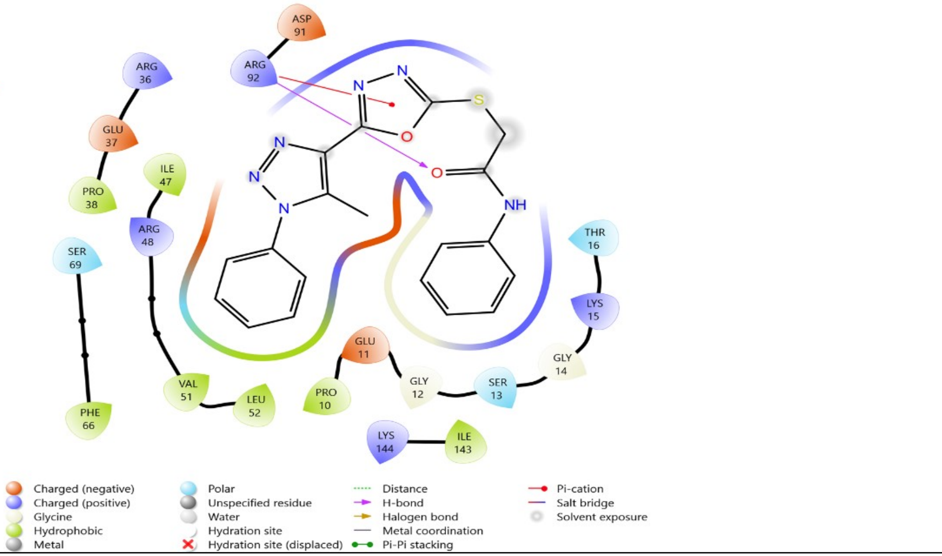

10b

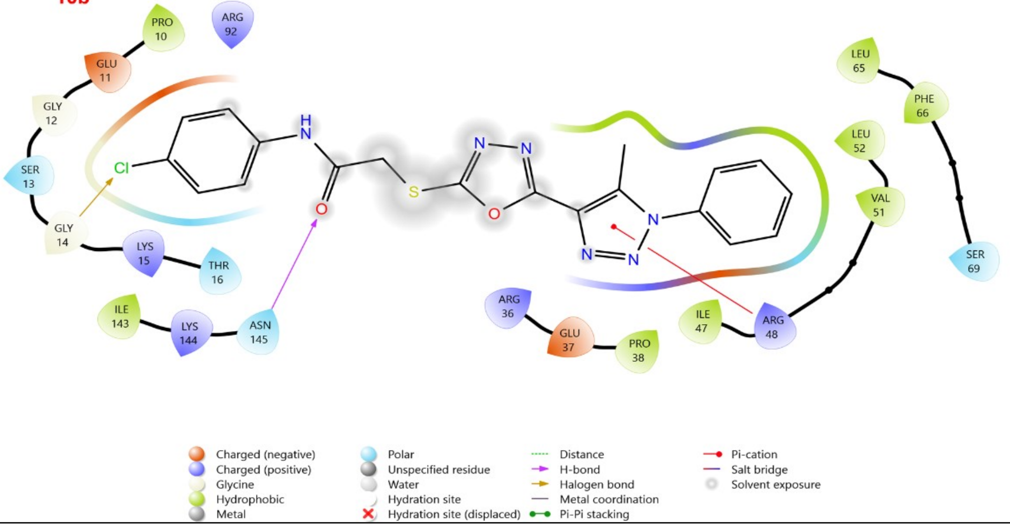

10c

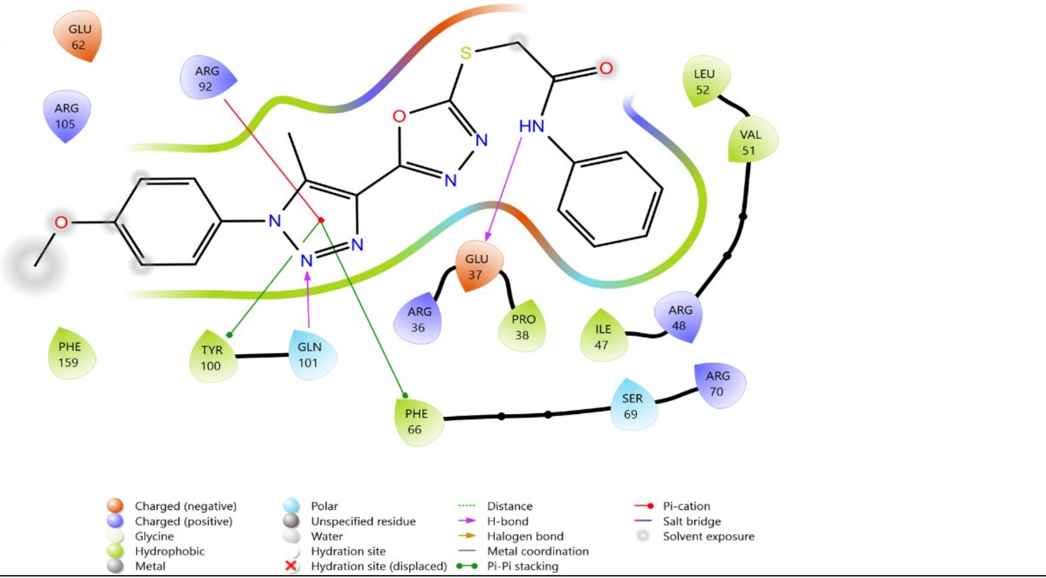

10d

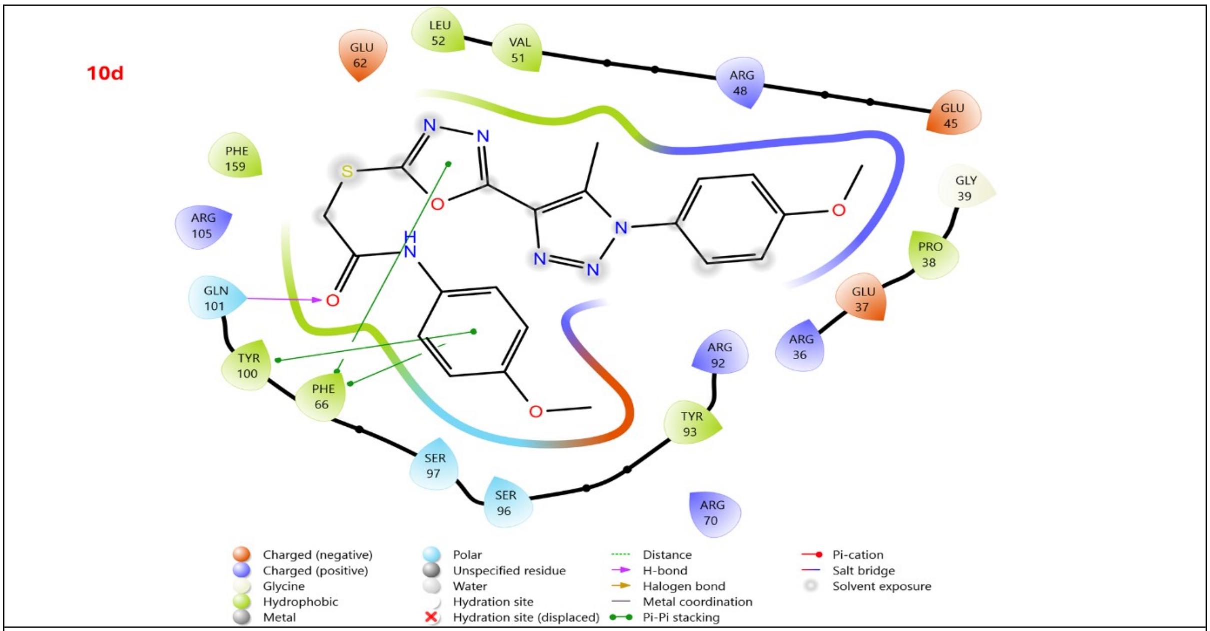

GLU 62
LEU 52
VAL 51
ARG 48
GLU 45
PHE 159
ARG 105
GLY 39
PRO 38
GLU 37
ARG 36
GLN 101
TYR 100
PHE 66
ARG 92
TYR 93
SER 97
SER 96
ARG 70
Charged (negative)
Charged (positive)
Glycine
Hydrophobic
Metal
Polar
Unspecified residue
Water
Hydration site
Hydration site (displaced)
Distance
H-bond
Halogen bond
Metal coordination
Pi-Pi stacking
Pi-cation
Salt bridge
Solvent exposure

10e

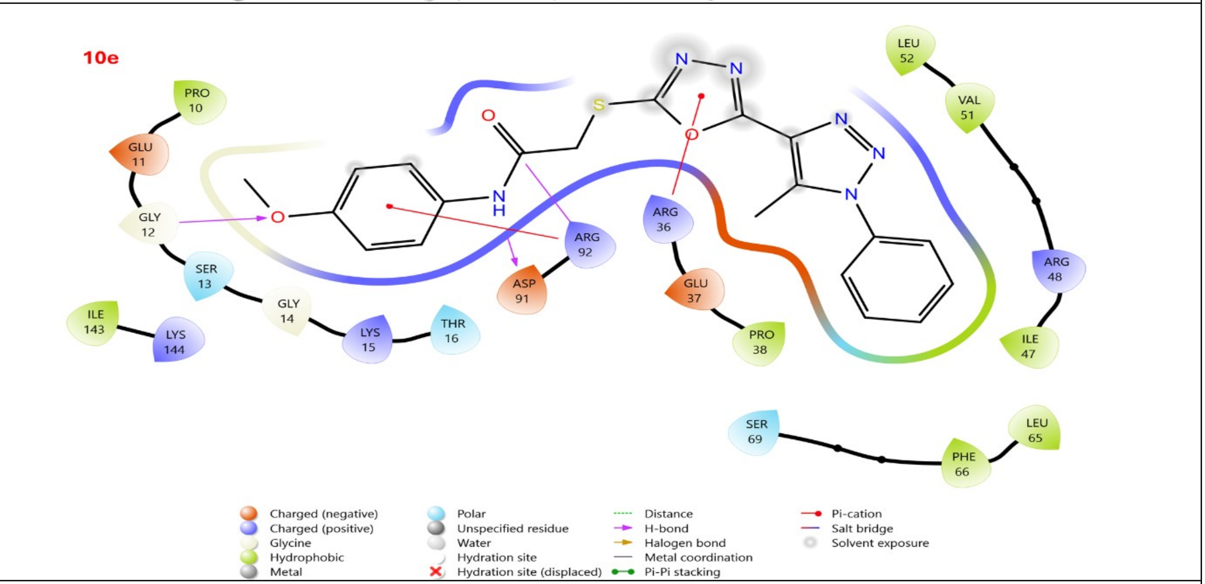

PRO 10
GLU 11
GLY 12
SER 13
ILE 143
LYS 144
GLY 14
LYS 15
THR 16
ASP 91
ARG 92
ARG 36
GLU 37
PRO 38
LEU 52
VAL 51
ARG 48
ILE 47
SER 69
PHE 66
LEU 65
Charged (negative)
Charged (positive)
Glycine
Hydrophobic
Metal
Polar
Unspecified residue
Water
Hydration site
Hydration site (displaced)
Distance
H-bond
Halogen bond
Metal coordination
Pi-Pi stacking
Pi-cation
Salt bridge
Solvent exposure

10f

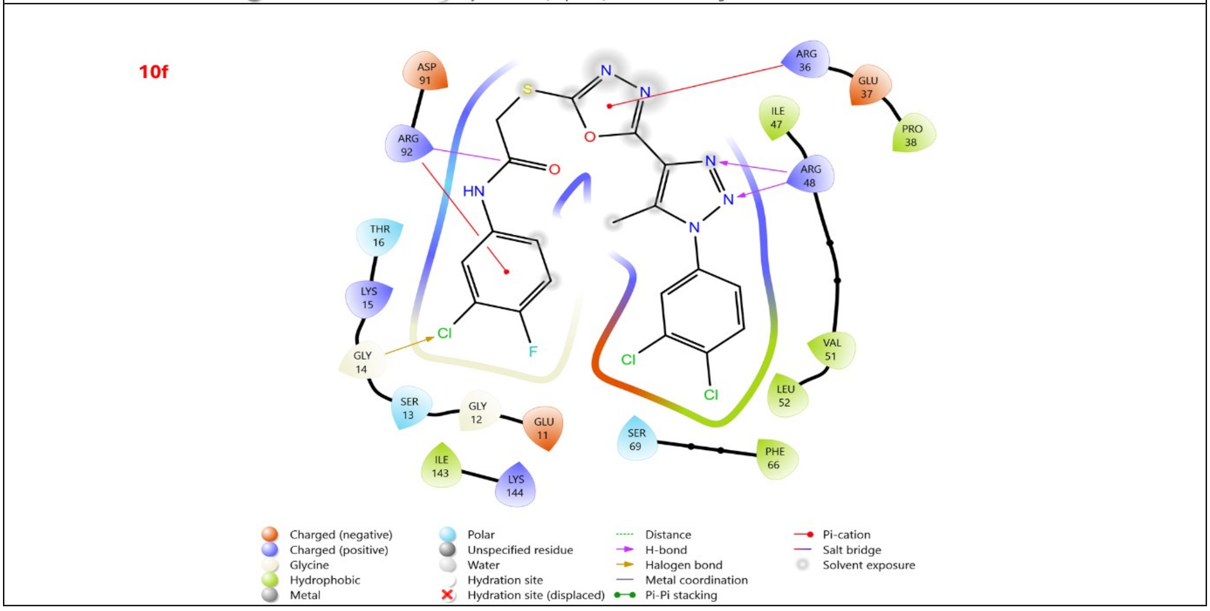

ASP 91
ARG 92
ARG 36
GLU 37
PRO 38
ILE 47
ARG 48
THR 16
LYS 15
GLY 14
SER 13
GLY 12
GLU 11
ILE 143
LYS 144
SER 69
PHE 66
VAL 51
LEU 52
Charged (negative)
Charged (positive)
Glycine
Hydrophobic
Metal
Polar
Unspecified residue
Water
Hydration site
Hydration site (displaced)
Distance
H-bond
Halogen bond
Metal coordination
Pi-Pi stacking
Pi-cation
Salt bridge
Solvent exposure

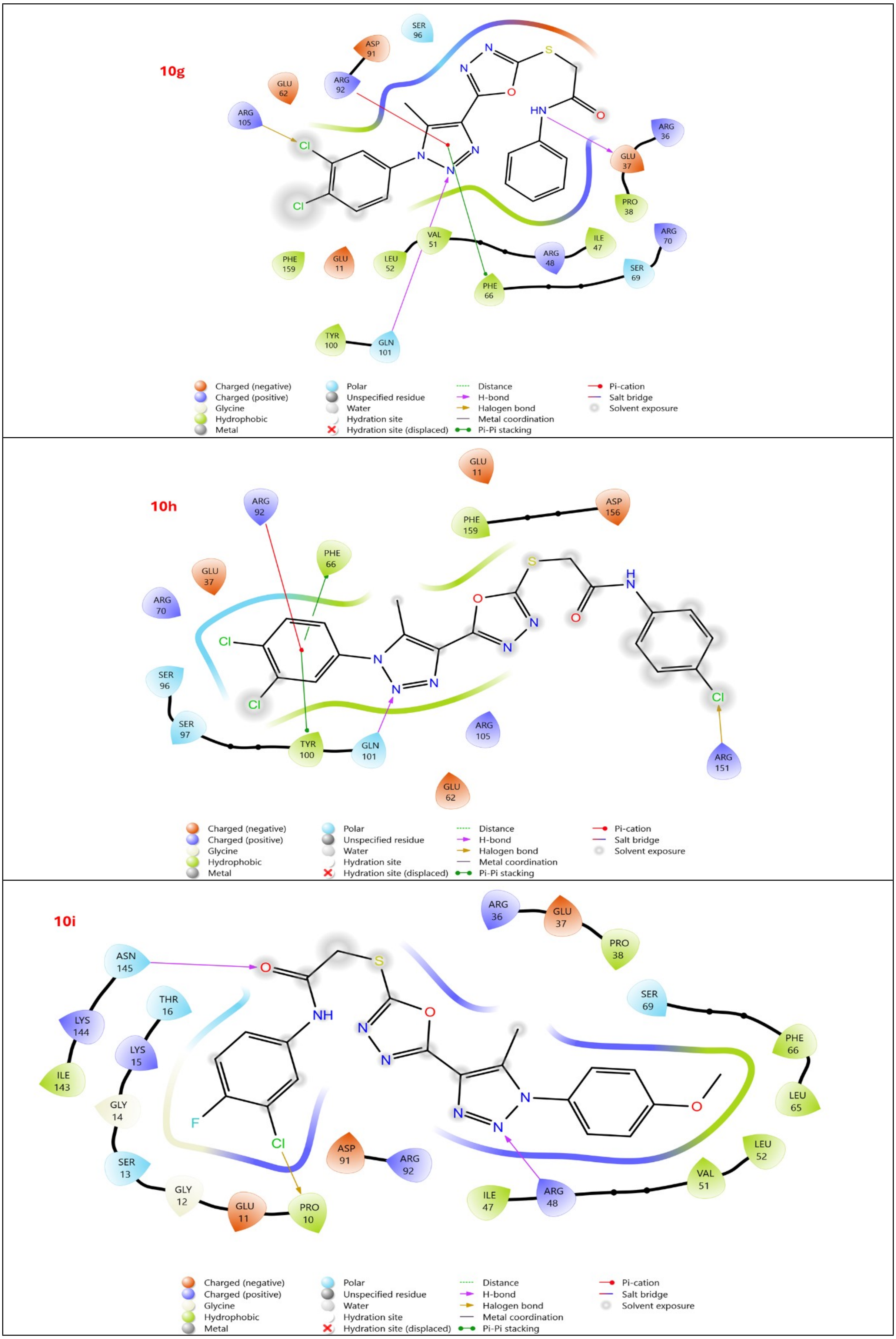
10g
SER 96
ASP 91
ARG 92
GLU 62
ARG 105
ARG 36
GLU 37
PRO 38
ARG 70
ILE 47
ARG 48
SER 69
VAL 51
LEU 52
GLU 11
PHE 159
PHE 66
TYR 100
GLN 101
10h
GLU 11
ASP 156
PHE 159
ARG 92
PHE 66
GLU 37
ARG 70
SER 96
SER 97
TYR 100
GLN 101
ARG 105
ARG 151
GLU 62
10i
ARG 36
GLU 37
PRO 38
ASN 145
THR 16
LYS 144
LYS 15
ILE 143
GLY 14
SER 13
GLY 12
GLU 11
PRO 10
ASP 91
ARG 92
SER 69
PHE 66
LEU 65
LEU 52
VAL 51
ARG 48
ILE 47
Charged (negative)
Charged (positive)
Glycine
Hydrophobic
Metal
Polar
Unspecified residue
Water
Hydration site
Hydration site (displaced)
Distance
H-bond
Halogen bond
Metal coordination
Pi-Pi stacking
Pi-cation
Salt bridge
Solvent exposure

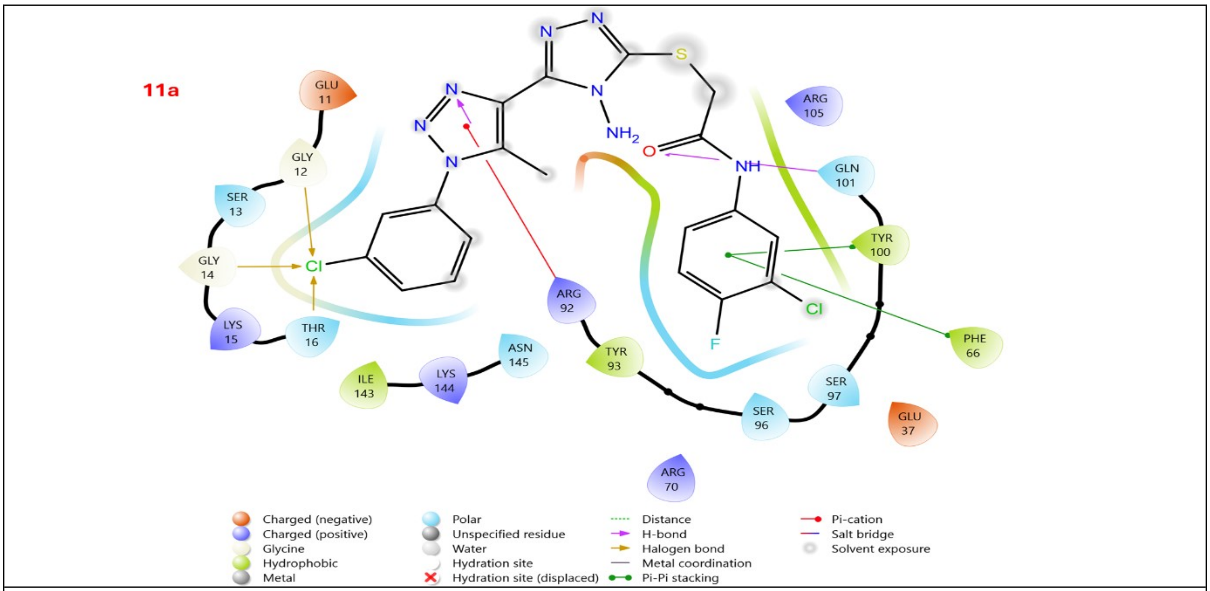

11a
GLU 11
GLY 12
SER 13
GLY 14
LYS 15
THR 16
ILE 143
LYS 144
ASN 145
ARG 92
TYR 93
SER 96
SER 97
GLU 37
ARG 70
PHE 66
TYR 100
GLN 101
ARG 105
N
N
N
N
N
N
NH2
S
O
NH
Cl
Cl
F
Charged (negative)
Charged (positive)
Glycine
Hydrophobic
Metal
Polar
Unspecified residue
Water
Hydration site
Hydration site (displaced)
Distance
H-bond
Halogen bond
Metal coordination
Pi-Pi stacking
Pi-cation
Salt bridge
Solvent exposure

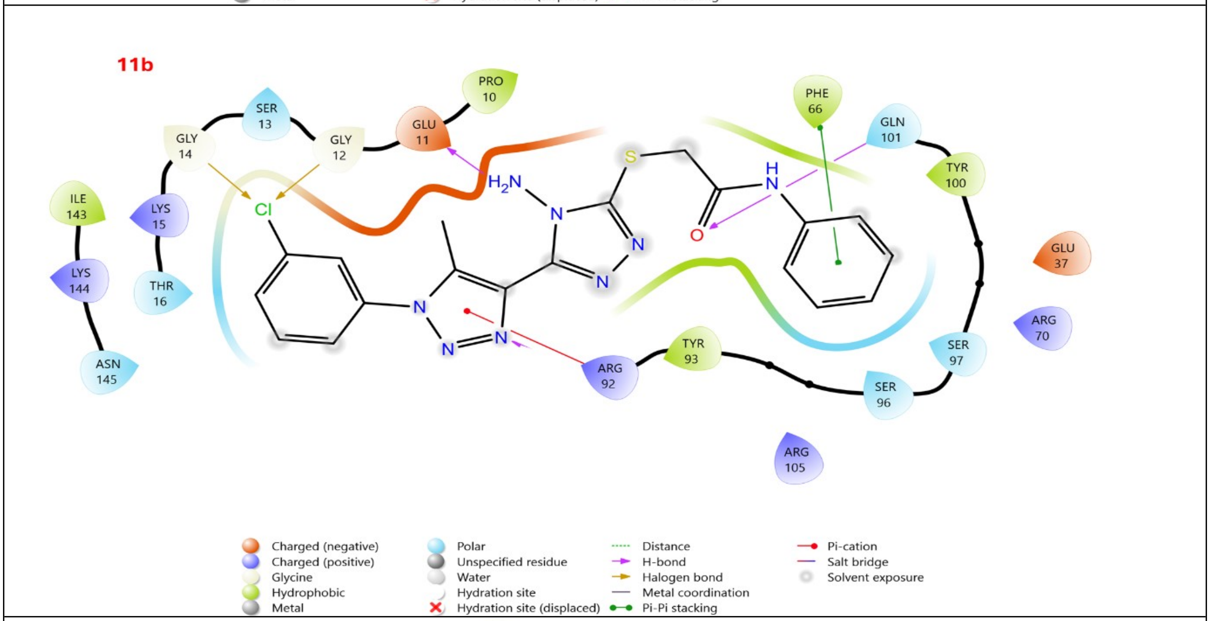

11b
PRO 10
GLU 11
GLY 12
SER 13
GLY 14
LYS 15
THR 16
ILE 143
LYS 144
ASN 145
PHE 66
GLN 101
TYR 100
GLU 37
ARG 70
SER 97
SER 96
TYR 93
ARG 92
ARG 105
H2N
S
H
N
O
N
N
N
N
N
N
Cl
Charged (negative)
Charged (positive)
Glycine
Hydrophobic
Metal
Polar
Unspecified residue
Water
Hydration site
Hydration site (displaced)
Distance
H-bond
Halogen bond
Metal coordination
Pi-Pi stacking
Pi-cation
Salt bridge
Solvent exposure

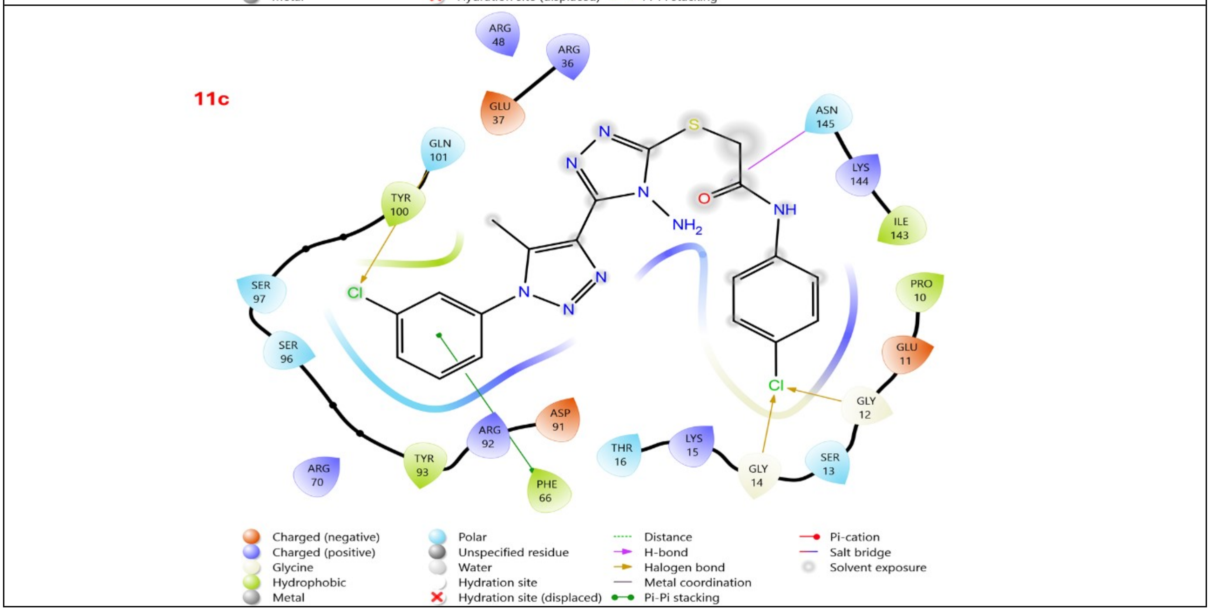

11c
ARG 48
ARG 36
GLU 37
GLN 101
TYR 100
SER 97
SER 96
TYR 93
ARG 92
ASP 91
PHE 66
ARG 70
THR 16
LYS 15
GLY 14
SER 13
GLY 12
GLU 11
PRO 10
ILE 143
LYS 144
ASN 145
S
N
N
N
O
NH2
NH
N
N
N
Cl
Cl
Charged (negative)
Charged (positive)
Glycine
Hydrophobic
Metal
Polar
Unspecified residue
Water
Hydration site
Hydration site (displaced)
Distance
H-bond
Halogen bond
Metal coordination
Pi-Pi stacking
Pi-cation
Salt bridge
Solvent exposure

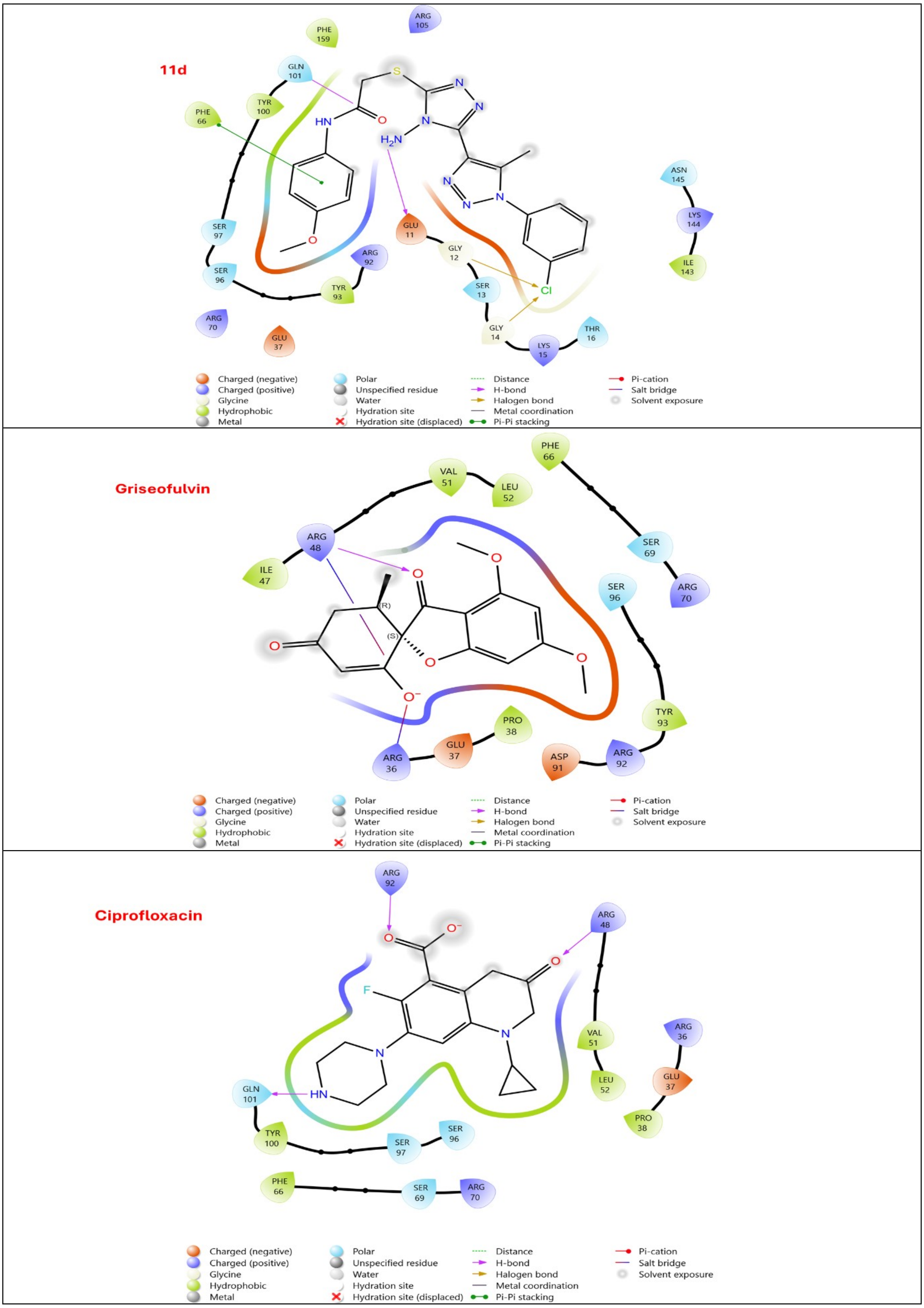

Figure S54. 2D and 3D Molecular docking diagram 4QGG

Table S5. Mulliken Atomic Charges of Selected Atoms in the Optimized Structure (Compound 10a)

Sr.no	Atom	Charge (Q/e)
1	C	-0.21063
2	C	-0.16062
3	C	-0.1248
4	C	-0.17213
5	C	-0.09089
6	C	0.30826
7	N	-0.78379
8	C	0.604418
9	O	-0.42618
10	C	-0.72479
11	C	-0.09236
12	C	-0.16636
13	C	-0.12243
14	C	-0.16853
15	C	-0.10656
16	C	0.268015
17	N	-0.79136
18	C	0.60372
19	C	-0.12251
20	N	-0.23884
21	N	0.059177
22	C	-0.56561
23	C	0.30224
24	O	-0.46553
25	C	0.209148
26	N	-0.24557
27	N	-0.19183
28	S	0.315321
29	H	0.170047
30	H	0.155923
31	H	0.156204
32	H	0.152452
33	H	0.216563
34	H	0.36762
35	H	0.276612
36	H	0.256826
37	H	0.190036
38	H	0.163951
39	H	0.162886
40	H	0.162039
41	H	0.190804
42	H	0.209528
43	H	0.253438
44	H	0.216053

Table S6. Mulliken Atomic Charges of Selected Atoms in the Optimized Structure (Compound 10b)

Sr.no	Atom	Charge (Q/e)
1	C	-0.16728
2	C	-0.10936
3	C	-0.22214
4	C	-0.1263
5	C	-0.09763
6	C	0.311985
7	N	-0.77287
8	C	0.546203
9	O	-0.44645
10	C	-0.56358
11	C	-0.08461
12	C	-0.14767
13	C	-0.10144
14	C	-0.14777
15	C	-0.10048
16	C	0.290597
17	N	-0.72125
18	C	0.504968
19	C	0.020598
20	N	-0.26028
21	N	0.037085
22	C	-0.44561
23	C	0.35157
24	O	-0.48747
25	C	0.204272
26	N	-0.3632
27	N	-0.21131
28	S	0.429492
29	Cl	0.055225
30	H	0.162974
31	H	0.160197
32	H	0.158683
33	H	0.192616
34	H	0.399637
35	H	0.222433
36	H	0.224287
37	H	0.17363
38	H	0.146555
39	H	0.142311
40	H	0.143156
41	H	0.148209
42	H	0.175032
43	H	0.180546
44	H	0.194394

Table S7. Mulliken Atomic Charges of Selected Atoms in the Optimized Structure (Compound 10c)

Sr.no	Atom	Charge (Q/e)	Sr.no	Atom	Charge (Q/e)
1	C	-0.20973	25	C	0.207438
2	C	-0.16075	26	N	-0.24551
3	C	-0.12505	27	N	-0.19261
4	C	-0.17218	28	S	0.314343
5	C	-0.09079	29	O	-0.51629
6	C	0.30777	30	C	-0.29518
7	N	-0.78386	31	H	0.170613
8	C	0.604696	32	H	0.155922
9	O	-0.42636	33	H	0.155851
10	C	-0.72557	34	H	0.151812
11	C	-0.07952	35	H	0.215681
12	C	-0.18012	36	H	0.368003
13	C	0.260531	37	H	0.277387
14	C	-0.17861	38	H	0.256375
15	C	-0.09965	39	H	0.186761
16	C	0.266729	40	H	0.18131
17	N	-0.79055	41	H	0.180025
18	C	0.60079	42	H	0.186387
19	C	-0.12014	43	H	0.209204
20	N	-0.24047	44	H	0.250505
21	N	0.057545	45	H	0.215402
22	C	-0.56601	46	H	0.207358
23	C	0.302208	47	H	0.188167
24	O	-0.46564	48	H	0.185784

Table S8. Mulliken Atomic Charges of Selected Atoms in the Optimized Structure (Compound 10d)

Sr.no	Atom	Charge (Q/e)	Sr.no	Atom	Charge (Q/e)
1	C	0.21087	27	N	-0.1775
2	C	-0.17347	28	S	0.370962
3	C	0.252531	29	O	-0.52425
4	C	-0.17967	30	O	-0.51494
5	C	-0.09279	31	C	-0.29568
6	C	0.305134	32	C	-0.29029
7	N	-0.79705	33	H	0.182083
8	C	0.591076	34	H	0.169146
9	O	-0.41702	35	H	0.167903
10	C	-0.73351	36	H	0.21458
11	C	-0.0808	37	H	0.407631
12	C	-0.17823	38	H	0.257157
13	C	0.260665	39	H	0.258129
14	C	-0.17864	40	H	0.189279
15	C	-0.096	41	H	0.183401
16	C	0.269666	42	H	0.180267
17	N	-0.78972	43	H	0.178423
18	C	0.617653	44	H	0.21833
19	C	-0.14028	45	H	0.219067
20	N	-0.24106	46	H	0.231063
21	N	0.063464	47	H	0.209081
22	C	-0.56359	48	H	0.188801
23	C	0.296702	49	H	0.186143
24	O	-0.46188	50	H	0.197462
25	C	0.240022	51	H	0.180894
26	N	-0.33074	52	H	0.181257

Table S9. Mulliken Atomic Charges of Selected Atoms in the Optimized Structure (Compound 10e)

Sr.no	Atom	Charge (Q/e)	Sr.no	Atom	Charge (Q/e)
1	C	-0.21102	25	C	0.239761
2	C	-0.17344	26	N	-0.33002
3	C	0.25252	27	N	-0.17675
4	C	-0.17953	28	S	0.372475
5	C	-0.09264	29	O	-0.5242
6	C	0.305333	30	C	-0.29035
7	N	-0.7972	31	H	0.181756
8	C	0.591058	32	H	0.169117
9	O	-0.41662	33	H	0.1681
10	C	-0.73354	34	H	0.214675
11	C	-0.0941	35	H	0.407461
12	C	-0.1643	36	H	0.257255
13	C	-0.12227	37	H	0.258581
14	C	-0.16825	38	H	0.192788
15	C	-0.10229	39	H	0.166264
16	C	0.270835	40	H	0.165143
17	N	-0.79043	41	H	0.162787
18	C	0.622286	42	H	0.182365
19	C	-0.14327	43	H	0.219562
20	N	-0.23937	44	H	0.221048
21	N	0.065596	45	H	0.232365
22	C	-0.56507	46	H	0.197569
23	C	0.297337	47	H	0.180966
24	O	-0.46176	48	H	0.181402

Table S10. Mulliken Atomic Charges of Selected Atoms in the Optimized Structure (Compound 10f)

Sr.no	Atom	Charge (Q/e)	Sr.no	Atom	Charge (Q/e)
1	C	-0.211	25	C	0.239245
2	C	-0.1732	26	N	-0.32718
3	C	0.253177	27	N	-0.17375
4	C	-0.17953	28	S	0.376732
5	C	-0.09234	29	Cl	0.05506
6	C	0.305294	30	Cl	0.057067
7	N	-0.7972	31	O	-0.52387
8	C	0.591634	32	C	-0.29058
9	O	-0.4153	33	H	0.180501
10	C	-0.73365	34	H	0.16966
11	C	-0.07503	35	H	0.168752
12	C	-0.06173	36	H	0.215258
13	C	-0.2019	37	H	0.406502
14	C	-0.24394	38	H	0.260012
15	C	0.001638	39	H	0.257095
16	C	0.299875	40	H	0.206939
17	N	-0.80049	41	H	0.203074
18	C	0.631012	42	H	0.219863
19	C	-0.14766	43	H	0.221325
20	N	-0.23364	44	H	0.227462
21	N	0.068973	45	H	0.238557
22	C	-0.57171	46	H	0.198192
23	C	0.299492	47	H	0.181643
24	O	-0.46201	48	H	0.181432

Table S11. Mulliken Atomic Charges of Selected Atoms in the Optimized Structure (Compound 10g)

Sr.no	Atom	Charge (Q/e)	Sr.no	Atom	Charge (Q/e)
1	C	-0.211	23	C	0.302763
2	C	-0.16051	24	O	-0.46509
3	C	-0.12422	25	C	0.211039
4	C	-0.17255	26	N	-0.24465
5	C	-0.09032	27	N	-0.18906
6	C	0.307539	28	S	0.320701
7	N	-0.78238	29	Cl	0.05237
8	C	0.603815	30	Cl	0.05292
9	O	-0.42676	31	H	0.169798
10	C	-0.72376	32	H	0.156689
11	C	-0.07301	33	H	0.157528
12	C	-0.06407	34	H	0.154723
13	C	-0.20055	35	H	0.216917
14	C	-0.24342	36	H	0.36668
15	C	-0.00411	37	H	0.275488
16	C	0.296382	38	H	0.258048
17	N	-0.80162	39	H	0.204775
18	C	0.608361	40	H	0.201361
19	C	-0.12382	41	H	0.229482
20	N	-0.23278	42	H	0.20932
21	N	0.061702	43	H	0.263181
22	C	-0.56813	44	H	0.219828

Table S12. Mulliken Atomic Charges of Selected Atoms in the Optimized Structure (Compound 10h)

Sr.no	Atom	Charge (Q/e)	Sr.no	Atom	Charge (Q/e)
1	C	-0.217	23	C	0.299083
2	C	-0.05874	24	O	-0.46044
3	C	-0.28666	25	C	0.241456
4	C	-0.06833	26	N	-0.33301
5	C	-0.08919	27	N	-0.17339
6	C	0.314619	28	S	0.38399
7	N	-0.80034	29	Cl	-0.02982
8	C	0.593751	30	Cl	0.055695
9	O	-0.40549	31	Cl	0.058649
10	C	-0.73565	32	H	0.191254
11	C	-0.07457	33	H	0.186539
12	C	-0.06142	34	H	0.184978
13	C	-0.20199	35	H	0.225272
14	C	-0.24381	36	H	0.410308
15	C	0.001055	37	H	0.260348
16	C	0.299953	38	H	0.263676
17	N	-0.80063	39	H	0.207653
18	C	0.631472	40	H	0.203861
19	C	-0.14749	41	H	0.219392
20	N	-0.23338	42	H	0.222827
21	N	0.070204	43	H	0.227376
22	C	-0.56977	44	H	0.237197

Table S13. Mulliken Atomic Charges of Selected Atoms in the Optimized Structure (Compound 10i)

Sr.no	Atom	Charge (Q/e)	Sr.no	Atom	Charge (Q/e)
1	C	-0.18209	25	C	0.242377
2	C	-0.1954	26	N	-0.34101
3	C	0.462048	27	N	-0.17659
4	C	-0.42553	28	S	0.382164
5	C	0.022963	29	O	-0.51436
6	C	0.307208	30	F	-0.31822
7	N	-0.80148	31	Cl	0.02974
8	C	0.594542	32	C	-0.29593
9	O	-0.40433	33	H	0.197857
10	C	-0.73642	34	H	0.187266
11	C	-0.07825	35	H	0.251729
12	C	-0.17871	36	H	0.414685
13	C	0.261586	37	H	0.260339
14	C	-0.17862	38	H	0.263882
15	C	-0.09462	39	H	0.18871
16	C	0.266226	40	H	0.184427
17	N	-0.78942	41	H	0.181114
18	C	0.620373	42	H	0.178134
19	C	-0.14271	43	H	0.220048
20	N	-0.24114	44	H	0.219856
21	N	0.065906	45	H	0.230448
22	C	-0.56331	46	H	0.209973
23	C	0.298765	47	H	0.189314
24	O	-0.46004	48	H	0.186495

Table S14. Mulliken Atomic Charges of Selected Atoms in the Optimized Structure (Compound 11a)

Sr.no	Atom	Charge (Q/e)	Sr.no	Atom	Charge (Q/e)
1	C	-0.18234	25	C	0.261939
2	C	-0.19592	26	N	-0.35623
3	C	0.459047	27	N	-0.27046
4	C	-0.42364	28	S	0.373402
5	C	0.019898	29	F	-0.32037
6	C	0.308335	30	Cl	0.027736
7	N	-0.8024	31	Cl	0.004681
8	C	0.591436	32	N	-0.48384
9	O	-0.4087	33	H	0.18915
10	C	-0.73597	34	H	0.180906
11	C	-0.08078	35	H	0.252463
12	C	-0.16357	36	H	0.41516
13	C	-0.01891	37	H	0.254987
14	C	-0.34293	38	H	0.255594
15	C	0.006926	39	H	0.198567
16	C	0.278581	40	H	0.176176
17	N	-0.79254	41	H	0.199506
18	C	0.628055	42	H	0.217265
19	C	-0.11861	43	H	0.218745
20	N	-0.32	44	H	0.218945
21	N	0.057196	45	H	0.248321
22	C	-0.57774	46	H	0.343778
23	C	0.475839	47	H	0.336975
24	N	-0.60466			

Table S15. Mulliken Atomic Charges of Selected Atoms in the Optimized Structure (Compound 11b)

Sr.no	Atom	Charge (Q/e)	Sr.no	Atom	Charge (Q/e)
1	C	-0.25547	25	C	0.312083
2	C	-0.19388	26	N	-0.45682
3	C	-0.2031	27	N	-0.40369
4	C	-0.20463	28	S	0.50825
5	C	-0.18466	29	N	-0.48877
6	C	0.374832	30	Cl	0.122486
7	N	-1.05989	31	H	0.218647
8	C	0.788325	32	H	0.195903
9	O	-0.60327	33	H	0.194933
10	C	-0.6583	34	H	0.199021
11	C	-0.13752	35	H	0.279934
12	C	-0.21248	36	H	0.487719
13	C	-0.13219	37	H	0.255463
14	C	-0.34369	38	H	0.259245
15	C	-0.10452	39	H	0.256266
16	C	0.348624	40	H	0.230779
17	N	-0.96311	41	H	0.249658
18	C	0.572867	42	H	0.264142
19	C	-0.02612	43	H	0.192384
20	N	-0.37782	44	H	0.200178
21	N	0.096162	45	H	0.252836
22	C	-0.43815	46	H	0.375118
23	C	0.698112	47	H	0.375043
24	N	-0.86094			

Table S16. Mulliken Atomic Charges of Selected Atoms in the Optimized Structure (Compound 11c)

Sr.no	Atom	Charge (Q/e)	Sr.no	Atom	Charge (Q/e)
1	C	-0.16737	25	C	0.226967
2	C	-0.10986	26	N	-0.38307
3	C	-0.22177	27	N	-0.31746
4	C	-0.12666	28	S	0.417142
5	C	-0.0988	29	Cl	0.049251
6	C	0.313946	30	N	-0.45248
7	N	-0.77518	31	Cl	0.09438
8	C	0.54313	32	H	0.154914
9	O	-0.45135	33	H	0.153086
10	C	-0.56181	34	H	0.157527
11	C	-0.07689	35	H	0.192791
12	C	-0.14711	36	H	0.401029
13	C	-0.07462	37	H	0.217177
14	C	-0.27238	38	H	0.216424
15	C	-0.06225	39	H	0.179584
16	C	0.300375	40	H	0.156879
17	N	-0.72834	41	H	0.171459
18	C	0.513292	42	H	0.177566
19	C	0.052906	43	H	0.173232
20	N	-0.34602	44	H	0.177033
21	N	0.036163	45	H	0.220621
22	C	-0.46227	46	H	0.347581
23	C	0.469969	47	H	0.329803
24	N	-0.60855			

Table S17. Mulliken Atomic Charges of Selected Atoms in the Optimized Structure (Compound 11d)

Sr.no	Atom	Charge (Q/e)	Sr.no	Atom	Charge (Q/e)
1	C	-0.212	27	N	-0.2716
2	C	-0.17404	28	S	0.362392
3	C	0.250368	29	O	-0.52647
4	C	-0.17836	30	N	-0.48502
5	C	-0.09526	31	Cl	0.002663
6	C	0.306488	32	C	-0.28987
7	N	-0.79856	33	H	0.173666
8	C	0.587832	34	H	0.16274
9	O	-0.42108	35	H	0.167143
10	C	-0.733	36	H	0.214952
11	C	-0.08159	37	H	0.408985
12	C	-0.16368	38	H	0.251981
13	C	-0.0197	39	H	0.249855
14	C	-0.34257	40	H	0.198352
15	C	0.006953	41	H	0.175124
16	C	0.279248	42	H	0.198434
17	N	-0.79269	43	H	0.21769
18	C	0.625779	44	H	0.216956
19	C	-0.11689	45	H	0.218652
20	N	-0.32022	46	H	0.250348
21	N	0.054874	47	H	0.342267
22	C	-0.57799	48	H	0.335808
23	C	0.474096	49	H	0.195929
24	N	-0.60426	50	H	0.180556
25	C	0.260454	51	H	0.181086
26	N	-0.34699			

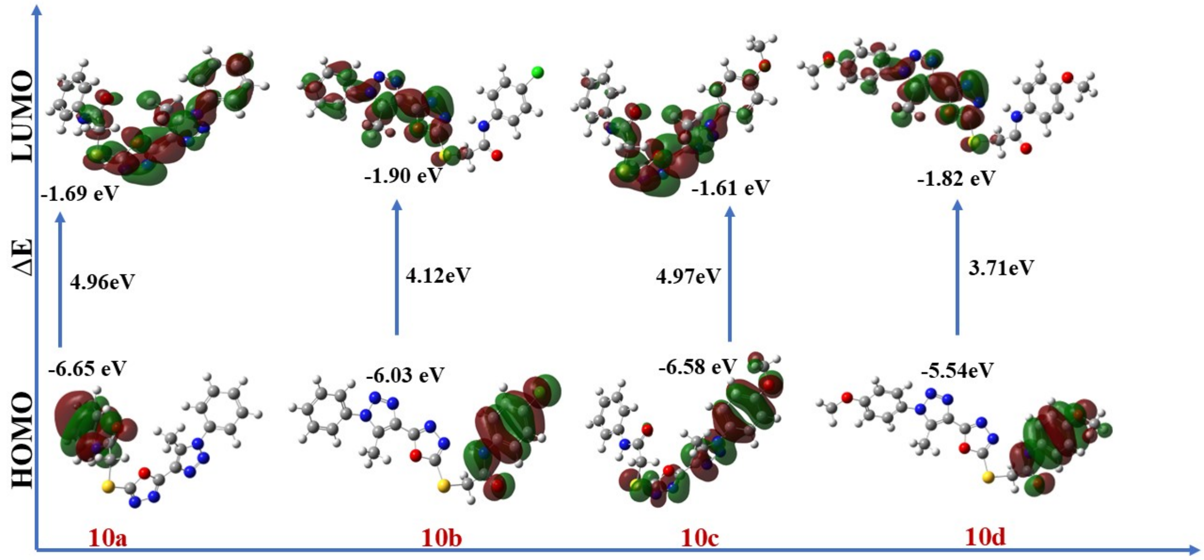

Figure S55. HOMO–LUMO energy level distribution and energy gap (ΔE) diagrams of compounds 10a–10d, showing electron density localization in frontier molecular orbitals. The visualized HOMO and LUMO surfaces reveal charge-transfer regions, highlighting the electronic transitions that govern molecular reactivity and stability.

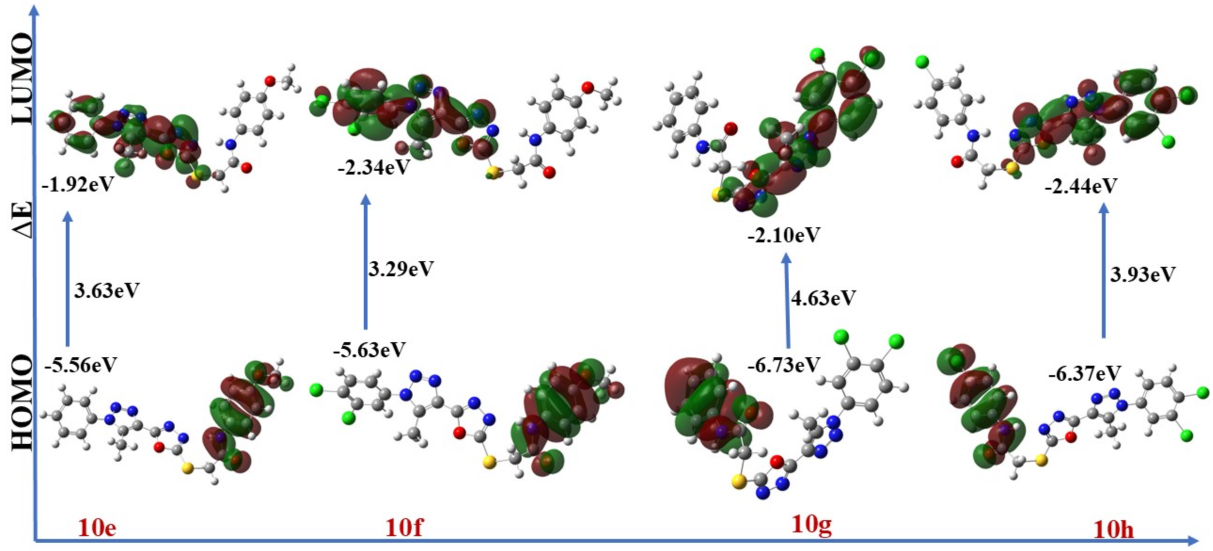

Figure S56. HOMO–LUMO energy level distribution and energy gap (ΔE) diagrams of compounds 10e–10h, illustrating the spatial electron density distribution in the frontier molecular orbitals. The visualization highlights intramolecular charge-transfer pathways that influence the electronic properties, reactivity, and stability of the compounds.

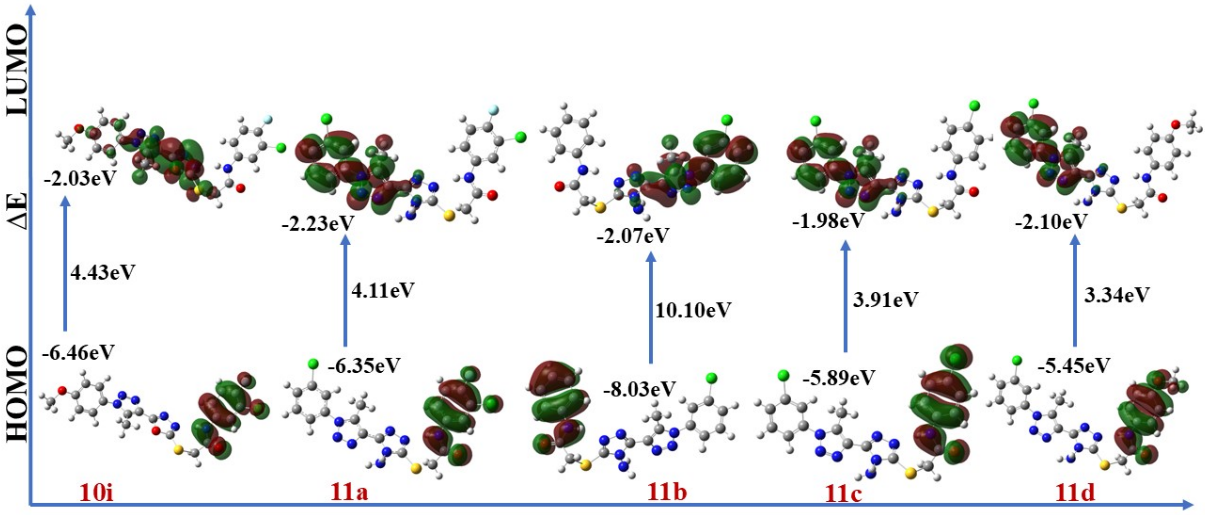

Figure S57: HOMO-LUMO electron density clouds and energy gaps (ΔE) for compounds 10i-11d, showing charge distribution and reactivity differences.

10a

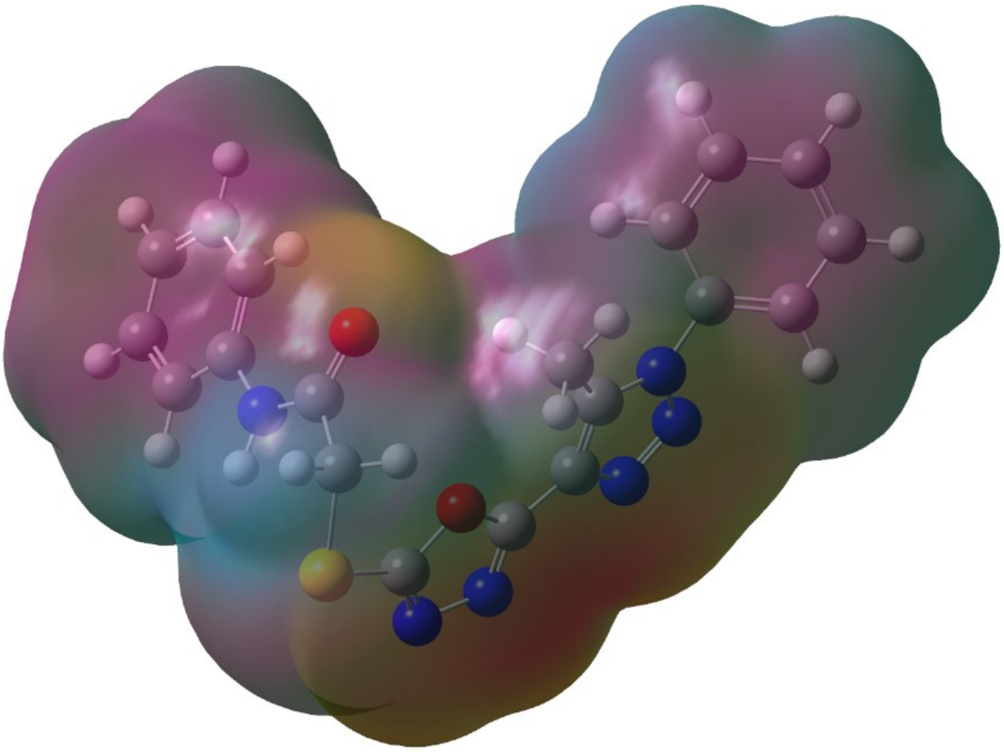

10b

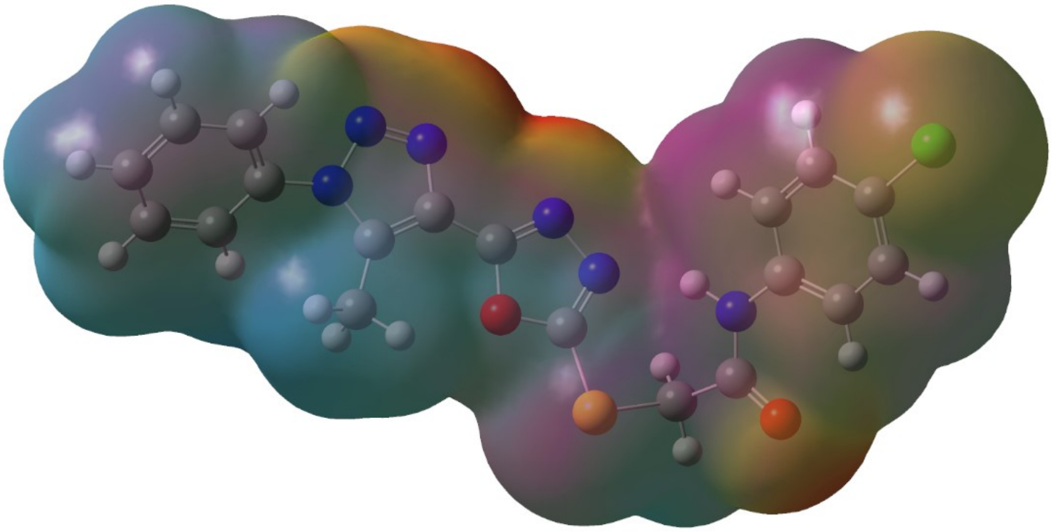

10c

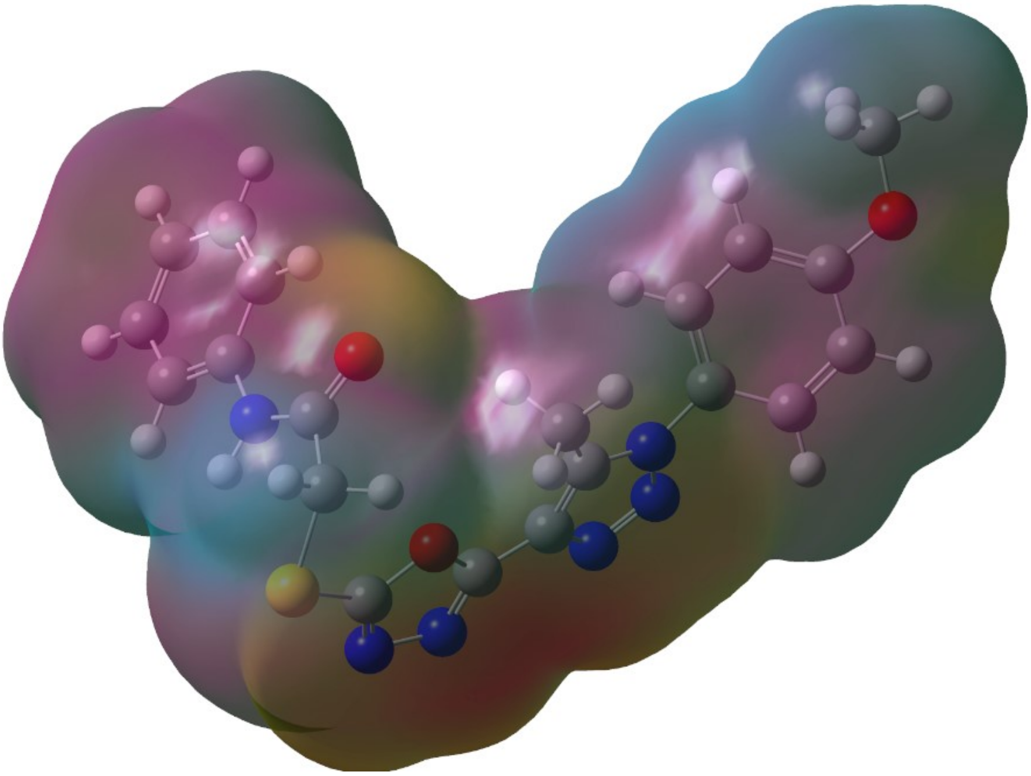

10d

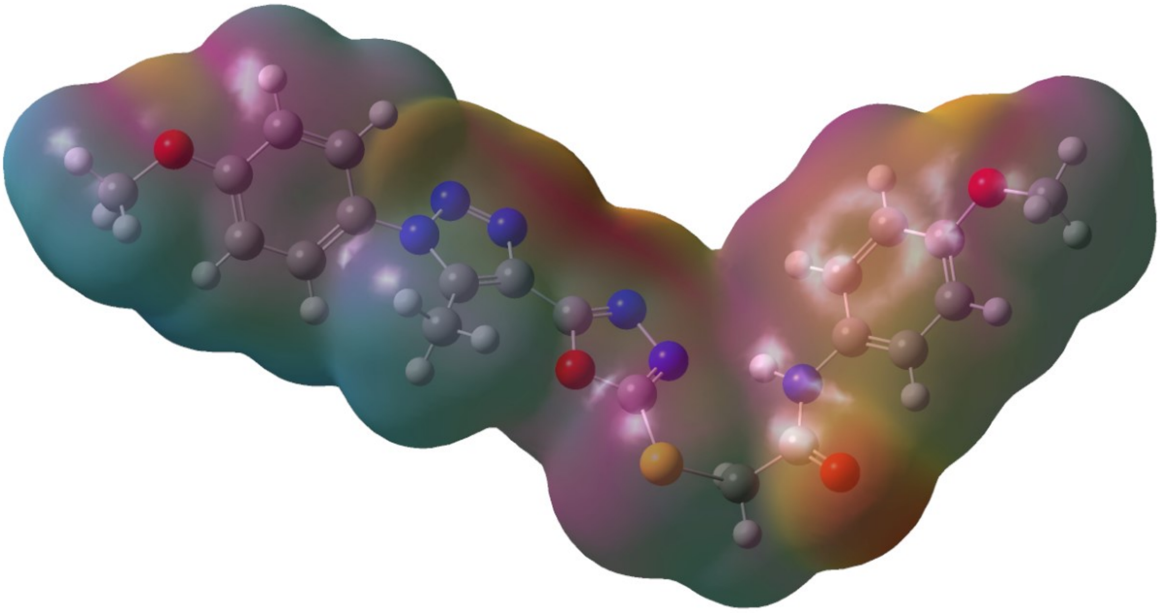

10e

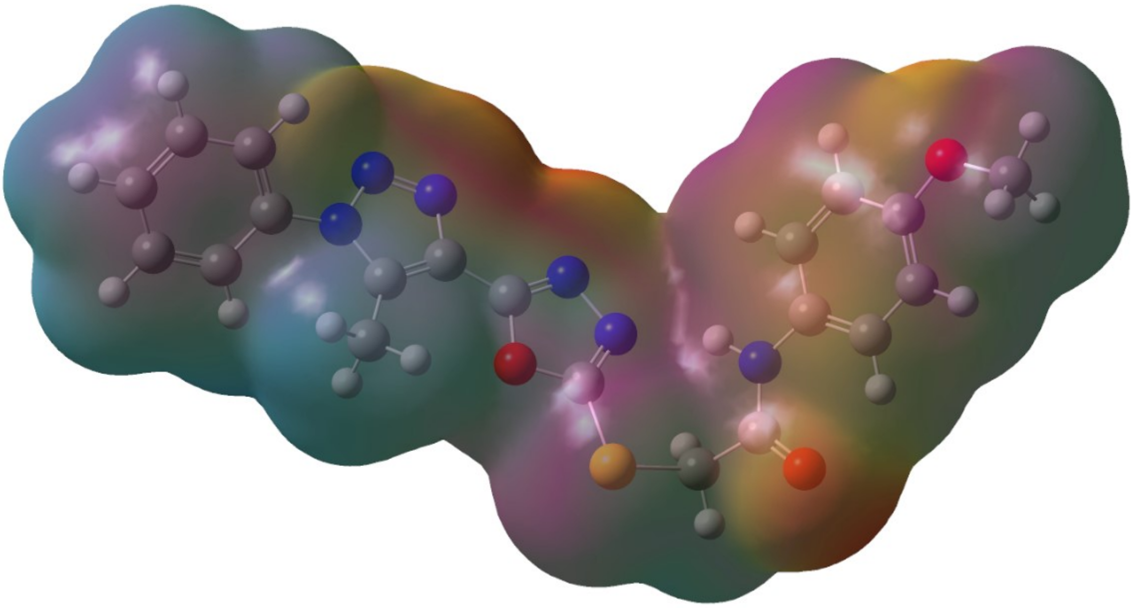

10f

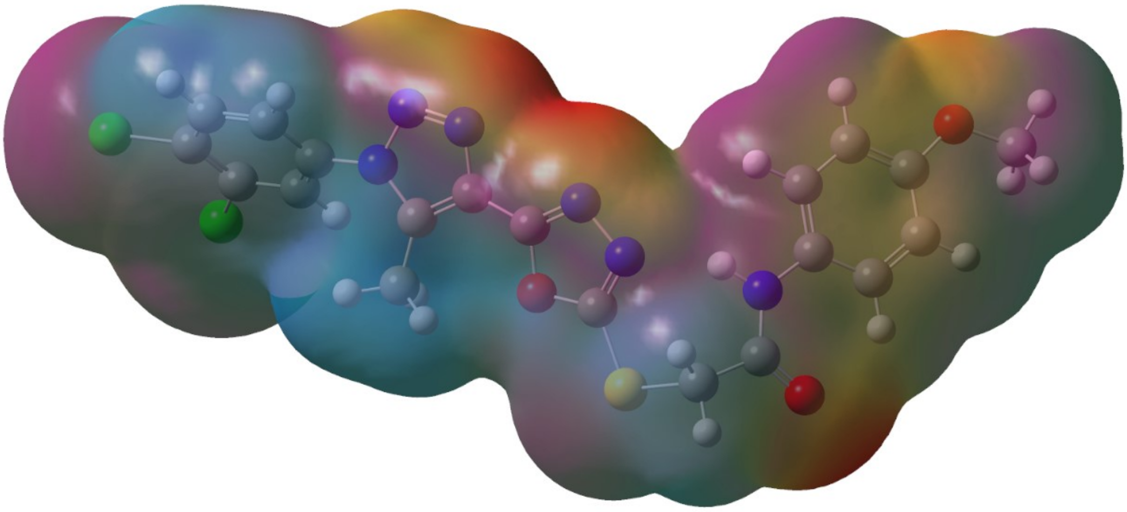

10g

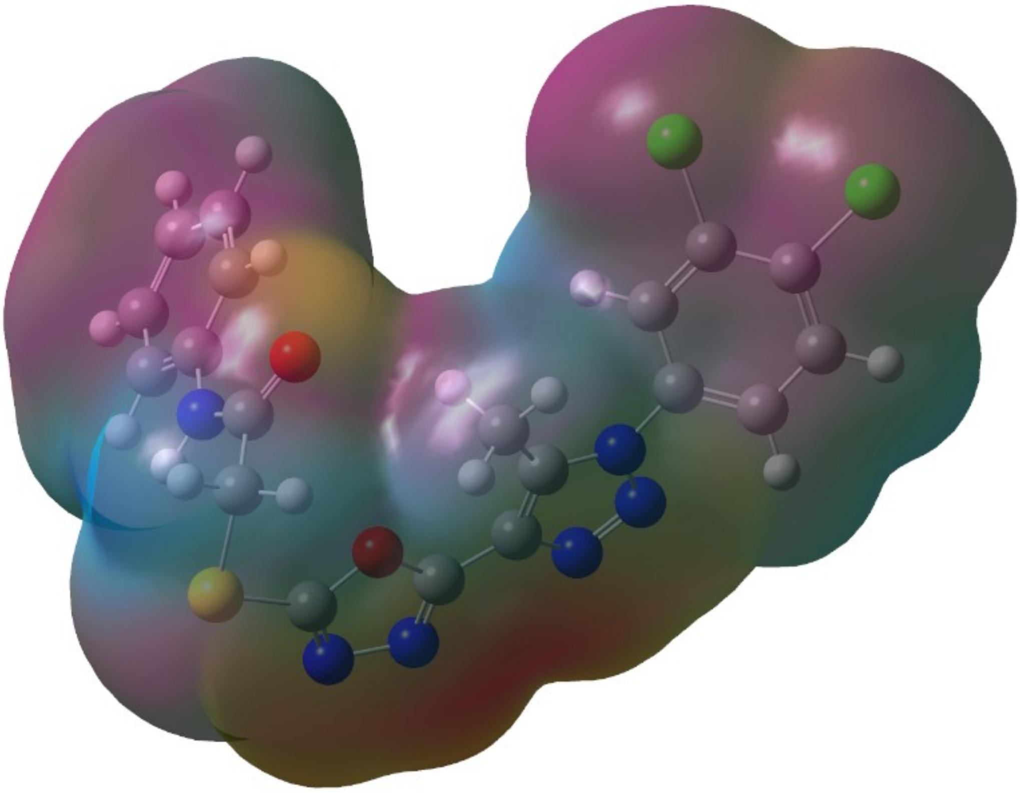

10h

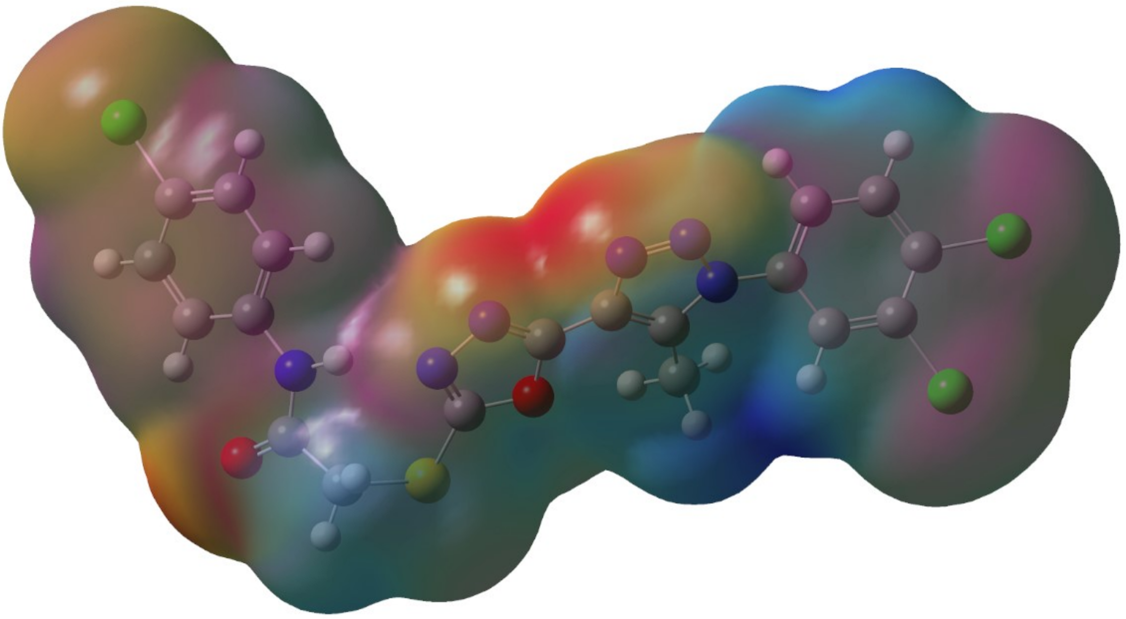

10i

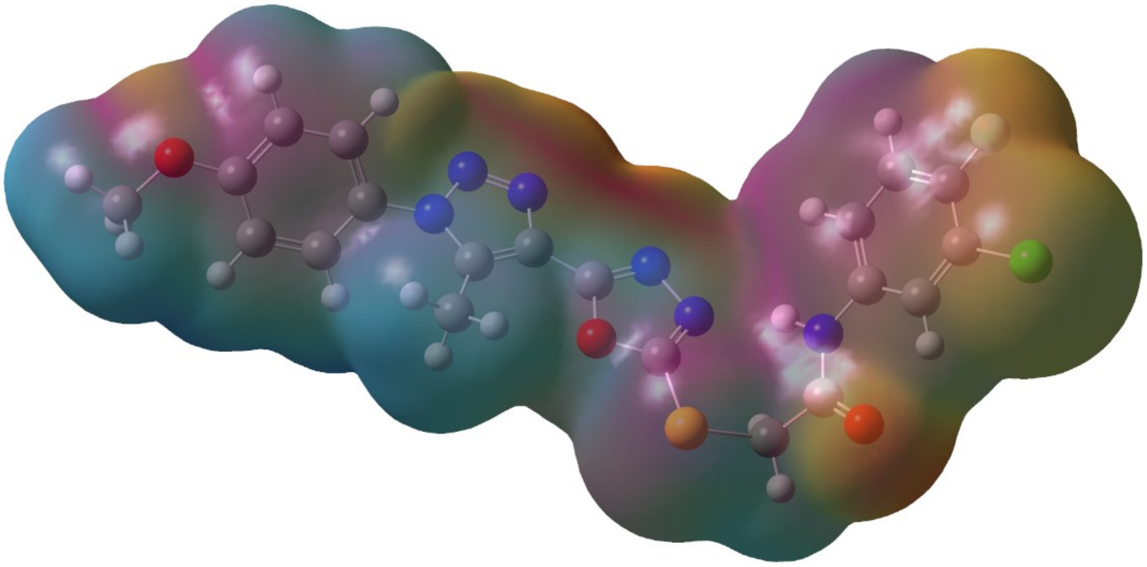

11a

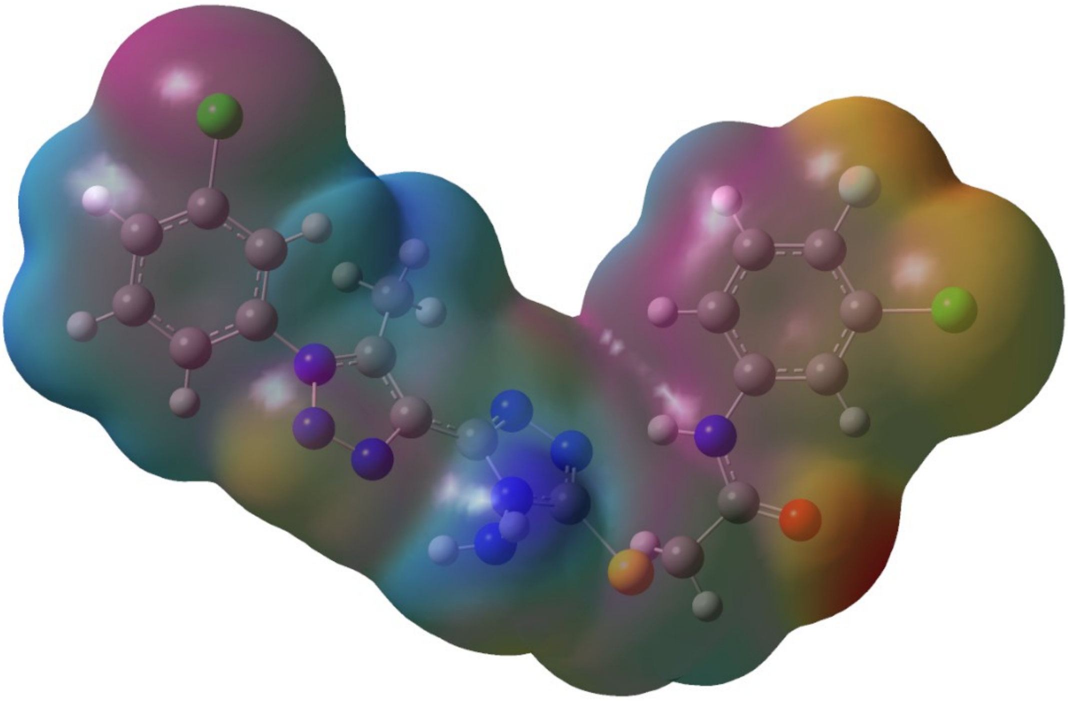

11b

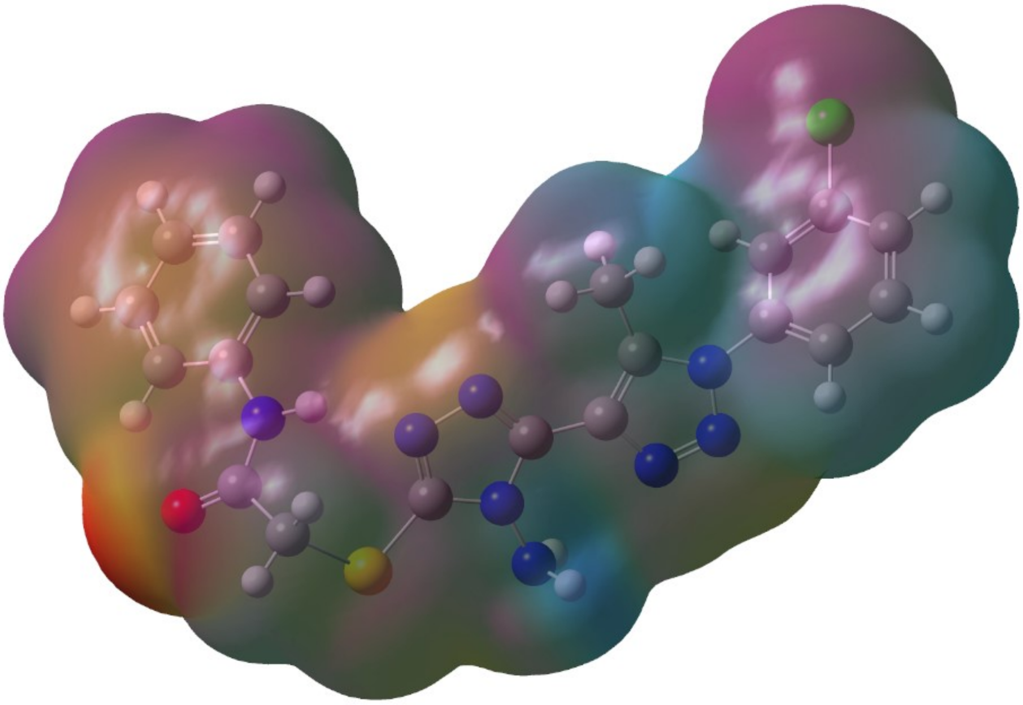

11c

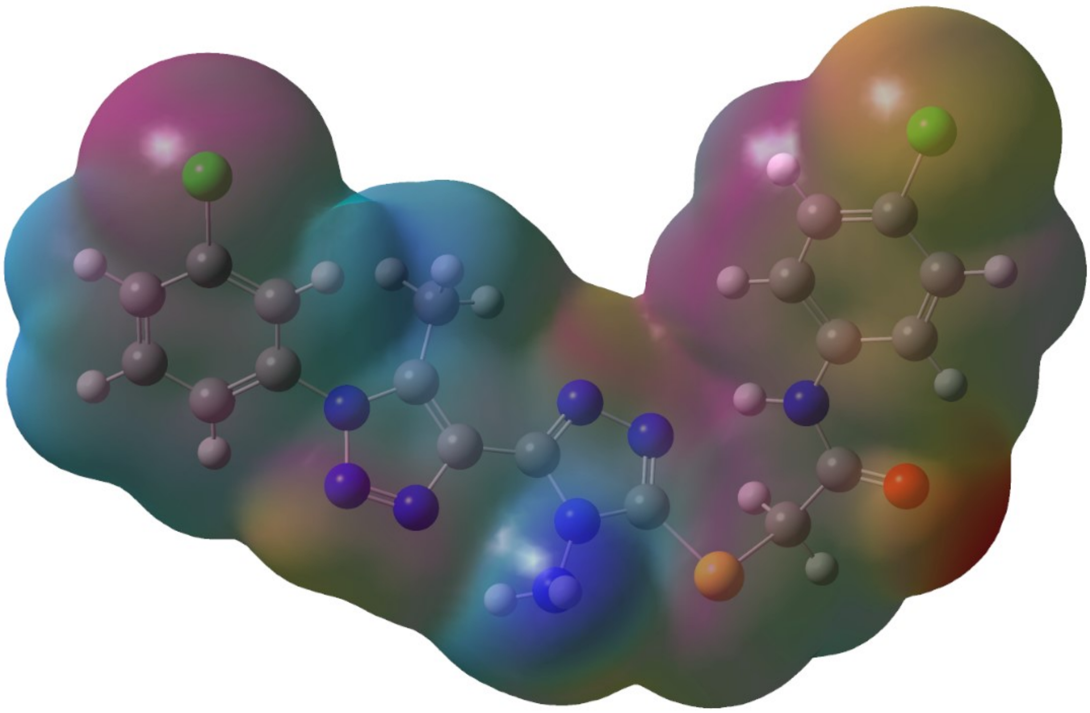

11d

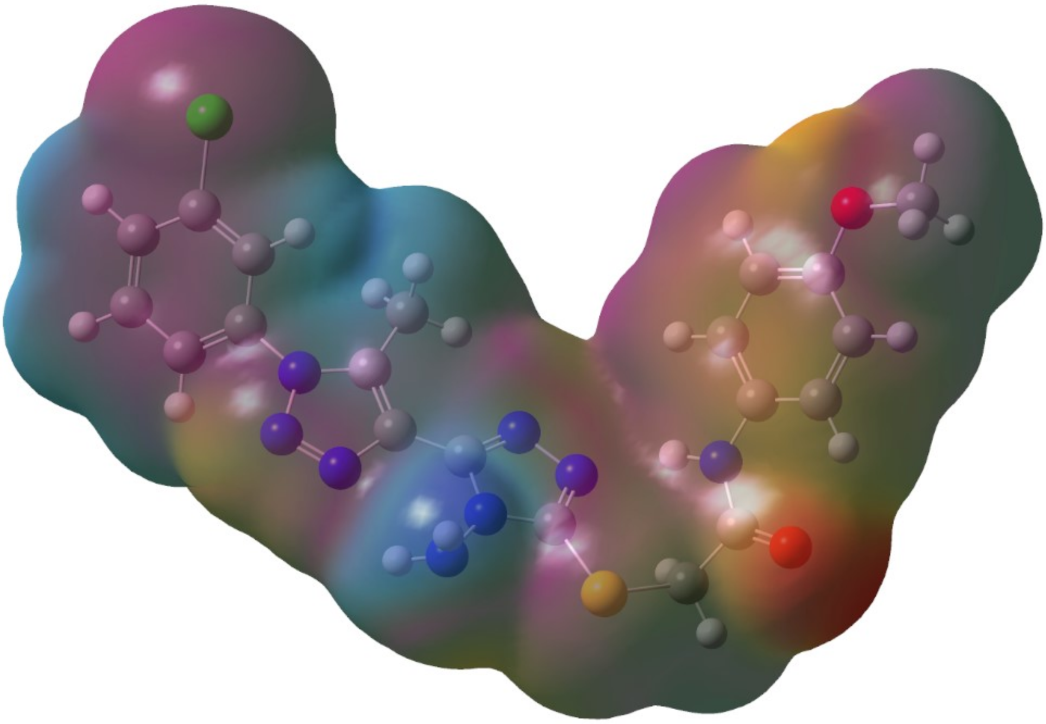

Figure S58 : 10a-l &11a-d ESP
